# Supplementary material for: HiFive: a tool suite for easy and efficient HiC and 5C data analysis
Source: Genome Biol. 2015 Oct 24;16:237. doi: 10.1186/s13059-015-0806-y (PMC5410870; doi:10.1186/s13059-015-0806-y)
Supplement: Supplementary file 1 — A detailed methods section, a table listing the sources of datasets used, and Supplemental figures. (PDF 2245 kb) [file 13059_2015_806_MOESM1_ESM.pdf]

Table of Contents

1 Supplemental Methods 3

1.1 Acquiring and mapping data 3

1.1.1 Mapping 5C data 3

1.1.2 Mapping HiC data 3

1.2 HiFive data normalization 3

1.2.1 5C filtering 4

1.2.2 5C distance dependence function estimation 4

1.2.3 5C normalization - Probability 5

1.2.4 5C normalization - Express 7

1.2.5 5C normalization - Binning 8

1.2.6 HiC filtering 9

1.2.7 HiC distance dependence function estimation 10

1.2.8 HiC normalization - Probability 11

1.2.9 HiC normalization - Express 13

1.2.10 HiC normalization - Binning 14

1.3 Other method data normalizations 16

1.3.1 HiCPipe HiC normalization 16

1.3.2 HiCNorm HiC normalization 17

1.3.3 HiCLib HiC normalization 17

1.3.4 Matrix-balancing HiC normalization 17

Data analysis 18

1.4 18

1.4.1 HiC probability model performance 18

1.4.2 5C-HiC data correlations 19

1.4.3 HiC dataset correlations 20

1.4.4 HiC normalization runtime comparison 20

1.4.5 HiC normalization memory usage comparison 21

2 Supplemental References 21

3 Supplemental Table and Figures 24

Supplemental Table 1 – Sources of 5C and HiC datasets. 24

Supplemental Figure 1 - Proximity-mediated ligation assays. 25

Supplemental Figure 2 – 5C filtering scheme. 26

Supplemental Figure 3 – HiC filtering scheme. 27

Supplemental Figure 4 – HiC read pairings. 28

Supplemental Figure 5 – 5C distance function. 29

Supplemental Figure 6 – HiC distance function. 30

Supplemental Figure 7 – HiC probability algorithm model comparison. 31

Supplemental Figure 8 – HiC algorithm distance cutoff comparison. 32

Supplemental Figure 9 – 5C algorithm distance cutoff comparison. 33

Supplemental Figure 10 – 5C-HiCPipe analysis performance. 34

Supplemental Figure 11 - Effects of pseudo-counts. 35

Supplemental Figure 12 - Effects of distance dependence on normalization. 36

Supplemental Figure 13 - Maximum RAM usage by HiC analysis methods. 37

1

1 Supplemental Methods

1.1 Acquiring and mapping data

All datasets described in this paper were obtained from public sources and can be found in Supplemental Table 1.

1.1.1 Mapping 5C data

Data was downloaded from the Gene Expression Omnibus (GEO) website [1] and split into paired-end fastq files using Fastq-Dump v2.1.18 from the SRA toolkit. Read ends were mapping independently to probe sequences, also obtained from GEO, using Bowtie v0.12.7 [2] and the mapping settings "--phred33-quals --tryhard -m1 -5 3 -3 2 -v 2". For each dataset, replicates were combined after mapping.

1.1.2 Mapping HiC data

HiC data were obtained from GEO and split using Fastq-Dump v2.3.5. Read ends were mapped independently to either the mouse genome, build 9, or the human genome, build 19, using Bowtie v0.12.7 and the mapping settings "--tryhard --phred33-quals -m 1 -v 2". Reads were initially trimmed from the 3' end to 50 base pairs (bp). After each round of mapping, reads that failed to align were trimmed again from the 3' end, either 4 or 5 bp depending on if the read's initial length was less than 40 bp or not, respectively. Alignment was repeated until all reads aligned or were shorter than 21 bp. For each dataset, replicates were combined after mapping.

1.2 HiFive data normalization

All data processing and normalization using HiFive was performed using version 1.1.3.

1.2.1 5C filtering

5C read data were imported directly from the BAM alignment files and reads that either had a single mapped end or mapped to same-orientation probes were discarded. For each dataset, HiFive's iterative filtering was performed using a cutoff of 20 interactions per fragment and a minimum interaction size of 50 kilobases (Kb). The iterative filtering was accomplished as follows. For each valid (not removed from analysis) fragment, the number of non-zero interaction pairings longer than the size cutoff was found. Fragments with fewer interactions than the cutoff were removed. This was repeated until all fragment met the minimum interaction criterion.

1.2.2 5C distance dependence function estimation

The distance dependence estimation function for each 5C dataset was found using a power-law relationship [3], with parameter values derived from a linear regression between the log-transformed interaction sizes and the log-transformed observed reads for each valid (unfiltered) non-zero fragment pairing (Supplemental Fig. 5). So for an interaction between forward fragment *i* and reverse fragment *j* in region *n*, the expected distance dependent signal is defined as:

$$D(i,j) = \gamma \ln(d_{ij}) + \mu_{global} + \mu_n \qquad (1)$$

with the global and regional mean log-transformed interaction signals μ_{global} and μ_n, respectively, and the slope parameter from the linear regression γ. The value μ_{global} is the mean value of all valid log-transformed counts across the set of all regions N (2).

$$\mu_{global} = \frac{\sum_{n \in N} \sum_{i \in n} \sum_{j \in n}^{c_{ij}>0} \ln(c_{ij})}{\sum_{n \in N} \sum_{i \in n} \sum_{j \in n}^{c_{ij}>0} 1} \tag{2}$$

The value μ_n is used to rescale correction parameters to have a mean correction of zero (3).

$$\mu_n = \frac{\sum_{i \in n} \sum_{j \in n} (f_i + f_j)}{\sum_{i \in n} \sum_{j \in n} 1} \tag{3}$$

This parameter has a value of zero until normalization is performed using either the Express or Probability algorithms, at which time it is calculated and the fragment correction parameters are adjusted (4).

$$f_i^{'} = f_i - \frac{\mu_n}{2} \tag{4}$$

1.2.3 5C normalization - Probability

5C data normalized using HiFive's Probability algorithm were modeled using a lognormal distribution. Each region's correction values were learned independently. Prior to learning, correction values were initialized as the square root of the mean difference of log-transformed non-zero interaction values and distance-dependence signals (5).

$$f_i = \frac{\sum_{i \in n} \sum_{j \in n}^{c_{ij}>0} \left[\ln(c_{ij}) - D(i,j) \right]}{\sum_{i \in n} \sum_{j \in n}^{c_{ij}>0} 2} \tag{5}$$

Correction values were found using backtracking line gradient descent. Learning continued for a maximum of 1000 iterations and terminated early if the maximum correction parameter gradient fell below 5e-4. The expected value for each log-transformed interaction was calculated as the sum of its predicted distance-dependent signal, the correction value for the first fragment, and the correction value for the second fragment (6).

$$E_{ij} = D(i,j) + f_i + f_j \tag{6}$$

Because counts were discrete, the cost *C* function was not strictly calculated as originating from a lognormal distribution but instead was found as follows:

$$C = -\sum_{ij}^{c_{ij}>0} \left(\begin{array}{l} [1 - I(c_{ij})] \ln\left[\phi\left(\frac{\ln(c_{ij}) - E_{ij}}{\sigma} \right) \right] + \\ I(c_{ij}) \ln\left[\Phi\left(\frac{\ln(c_{ij} + 0.5) - E_{ij}}{\sigma} \right) - \Phi\left(\frac{\ln(c_{ij} - 0.5) - E_{ij}}{\sigma} \right) \right] \end{array} \right) \tag{7}$$

where Φ is the cumulative probability function for the standard normal distribution, ϕ is the probability density function for the standard normal distribution, and σ is the standard deviation prior to normalization (8).

$$\sigma = \sqrt{\frac{\sum_{n \in N} \sum_{i \in n} \sum_{j \in n}^{c_{ij}>0} \left[\ln(c_{ij}) - \gamma \ln(d_{ij}) - \mu_{global} \right]^2}{\left(\sum_{n \in N} \sum_{i \in n} \sum_{j \in n}^{c_{ij}>0} 1 \right) - 1}} \tag{8}$$

I is an indicator function (9).

$$I(c_{ij}) = \begin{cases} 1 & \left[\Phi\left(\frac{\ln(c_{ij}+0.5)-E_{ij}}{\sigma}\right) - \Phi\left(\frac{\ln(c_{ij}-0.5)-E_{ij}}{\sigma}\right)\right] \geq 2.3E-308 \\ 0 & otherwise \end{cases} \quad (9)$$

This indicator function is necessary to deal with the limited precision of floating-point values and the inability to resolve small differences in the standard normal CDF function for large values of *c*. For each iteration *t*, the learning rate *r* was set to 0.01 and then decreased by 50% until the Armijo value (10) fell below zero, indicating a sufficiently advantageous update of the correction parameters or twenty updates were performed on the learning rate.

$$Armijo = C_t - C_{t-1} + r\sum_{n\in N}\left(\sum_{i\in n}(\nabla f_i)^2 + \sum_{j\in n}(\nabla f_j)^2\right) \quad (10)$$

1.2.4 5C normalization - Express

5C data normalized using HiFive's Express and ExpressKR algorithms were corrected using a matrix balancing approach. Each region was handled independently (intra-regional interactions only). For ExpressKR learning, estimated distance dependent signals were calculated for all non-zero interactions. For the standard Express algorithm, corrections were iteratively calculated as follows for fragment *i* with valid non-zero interactions A_i (11). Learning was run for 1000 iterations.

$$f_i^{'} = f_i + \frac{\sum_{i,j}^{c_{ij}\in A_i}\left[\ln(c_{ij}) - E_{ij}\right]}{\sum_{i,j}^{c_{ij}\in A_i} 2} \quad (11)$$

The ExpressKR algorithm used the matrix balancing algorithm described by Knight and Ruiz [4]. All non-zero counts were log-transformed prior to learning and the estimated distance dependent signal was subtracted from each. In order to achieve convergence, a pseudo-count of one was added to the matrix diagonal after log-transforming values. Learning was run until the residual fell below 1e-12.

Subsequent to learning correction values using either algorithm, values were adjusted so that the mean correction adjustment across all valid forward-reverse combinations for a region was zero (4).

1.2.5 5C normalization - Binning

5C data normalized using HiFive's Binning algorithm were corrected using an adaptation of Yaffe and Tanay's approach [5]. Each region was handled independently (intra-regional interactions only). The model used two features, fragment length and GC content. GC content was calculated as the percentage of guanine and cytosine in sequence-specific probe and spacer sequences of each primer. Both model features were partitioned into five intervals such that each interval contained an equal number of fragments regardless of probe orientation. Log-transformed reads were modeled as arising from a normal distribution with a mean for each interaction equal to the sum of the bin corrections *P* corresponding the fragment pair with the forward and reverse fragments falling in feature intervals *a* and *b*, respectively, for each feature *k* across the total set of features *K* and the estimated distance dependent signal (12).

$$E_{ij} = D(i,j) + \sum_{k \in K} P_{kab} \qquad (12)$$

Seed values for feature corrections were calculated as the mean of the log-transformed reads minus distance-dependence predictions for each feature bin divided by the number of observations in the bin (13).

$$P_{kab} = \frac{\sum_{i \in a} \sum_{j \in b}^{c_{ij}>0} \left[\ln(c_{ij}) - D(i,j) \right]}{\sum_{i \in a} \sum_{j \in b}^{c_{ij}>0} 1} \tag{13}$$

Learning was accomplished iteratively with each feature correction values being optimized independently for each iteration. Learning continued for a maximum of 1000 iterations and was stopped early if the log-likelihood changed by less than 1.0 for a given iteration. Optimization of correction values was done using the Broyden-Fletcher-Goldfarb-Shanno algorithm.

1.2.6 HiC filtering

Reads from aligned HiC data were loaded directly from BAM files, discarding reads that had a single mapped end. Reads were then assigned to fragment ends (fends) based on mapping within a fragment's boundaries and the orientation of alignment. Reads that mapped outside the first or last restriction enzyme (RE) cut site were discarded. Read end pairs that had a total distance sum between alignment coordinates and their respective downstream RE cut site (insert size) that was less than 500 bp were discarded. For cases with multiple reads mapping to the same pairs of coordinates, only one read was kept. In addition, reads that originated from the same fragment or that originated from

adjacent fragments and had opposite orientations were also discarded (Supplemental Fig. 4).

For each dataset, HiC fends were filtered using HiFive's iterative filtering using a cutoff of 10 interactions per fragment and an interaction size minimum of 500 Kb. For each valid (not removed from analysis) fend, the number of non-zero interactions greater than 500 Kb in size with other valid fends was counted. Fends with fewer interactions than the cutoff were removed. This was repeated until all fends met the minimum interaction criterion. The one exception was data for the human GM12878 MboI dataset normalized with HiFive's probability algorithm had an upper range limit of 10 megabases (Mb) on interaction sizes in addition to the lower limit for fend filtering. This was done to ensure all fends would have sufficient numbers of interactions for normalization since the same size limit was also employed then.

1.2.7 HiC distance dependence function estimation

HiC distance dependence estimation functions were calculated for each dataset using a piecewise linear approximation (Supplemental Fig. 6). Each genome's range was partitioned into 100 bins with the smallest bin covering interactions ranging from 0 to 1000 bp. The 99 remaining bins covered the range from 1001 bp to the largest possible interaction size (last fend midpoint minus the first fend midpoint of chromosome 1). This range was partitioned such that the log-transformed distance intervals each bin covered was equal with upper and lower limits for bin n denoted by U_n and L_n, respectively. For every possible valid

fend combination between fends i and j, defined as set A, the log-transformation of the distance d_{ij}, and the observed count c_{ij} (set to one if greater than zero for the binary version of the function). The estimated distance dependence signal is calculated as falling on the line intersecting the nearest two bins as determined by interaction distance (14-16).

$$X_n = \frac{\sum_{i,j}^{L_n \le d_{ij} < U_n} \ln(d_{ij})}{\sum_{i,j}^{L_n \le d_{ij} < U_n} 1} \tag{14}$$

$$Y_n = \ln\left(\frac{\sum_{i,j}^{L_n \le d_{ij} < U_n} c_{ij}}{\sum_{i,j}^{L_n \le d_{ij} < U_n} 1}\right) \tag{15}$$

$$D(i,j) = \frac{Y_{n+1} - Y_n}{X_{n+1} - X_n} \ln(d_{ij}) + Y_n - X_n \frac{Y_{n+1} - Y_n}{X_{n+1} - X_n} + \ln(\mu_m) \tag{16}$$

where μ_m is the chromosome mean correction adjustment which is used to give each intra-chromosomal Express and Probability algorithm correction pairing (excluding self-interactions) a mean value of 1 for chromosome m and correction parameter f_i (17).

$$\mu_m = \frac{\left(\sum_{i \in m} f_i\right)^2 - \sum_{i \in m} (f_i)^2}{\sum_{i \in m} 1 - \left(\sum_{i \in m} (f_i) - 1\right)} \tag{17}$$

The Binning algorithm corrections do not require this adjustment because they are calculated globally so μ_m is given a value of one.

1.2.8 HiC normalization - Probability

HiC data normalized using HiFive's Probability algorithm were modeled using a binomial distribution. The Poisson distribution is also available within HiFive as a distribution model but was not used due to poorer performance (see Supplemental Methods: HiC probability model performance). Each chromosome's correction values were learned independently and only interactions with an interaction distance greater than 500 Kb were used in calculations. This value was selected to eliminate the majority of domain structures while retaining enough reads for accurate normalization. For the human GM12878 MboI data, an upper distance limit of 10 Mb was used in order to fit within memory requirements (1 terabyte), since every interaction within the distance boundaries is used throughout model learning. Prior to normalization the estimated binary distance-dependent signal for each valid interaction (neither fend had been filtered out) was calculated and used as a prior for the interaction's probability of observation. The expected value for each interaction was calculated as the product of the exponent of the estimated distance dependence signal and both fend correction values (18).

$$E_{ij} = e^{D(i,j)} f_i f_j \quad (18)$$

Correction values were found using backtracking line gradient descent. Learning was continued for a maximum of 1000 iterations and terminated early if the maximum absolute correction parameter gradient fell below 5e-4. For each iteration *t*, the learning rate *r* was set to 1.0 and the Armijo value was calculated (19).

$$Armijo = C_t - C_{t-1} + r\sum_{i \in A}(\nabla f_i)^2 \quad (19)$$

If the Armijo value was greater than zero, the learning rate was in half and the new cost and Armijo value was calculated.

After learning correction values for a chromosome, μ_m was calculated and the square root of this mean was divided from the correction values, centering them such that the mean correction equaled one (20).

$$f_i' = \frac{f_i}{\sqrt{\mu_m}} \quad (20)$$

1.2.9 HiC normalization - Express

HiC data normalized using HiFive's Express and ExpressKR algorithms were corrected using a matrix-balancing approach. Each chromosome was handled independently (intra-chromosomal interactions only) and only interactions with an interaction distance greater than 500 Kb were used for calculations. Prior to learning correction values, estimated distance dependent signals were calculated for all non-zero interactions except for the data marked "ExpressKR" in Figure 4 and Supplemental Figure 13, which were given the estimated signal of one. For the standard Express algorithm, corrections were iteratively updated (21) over 1000 iterations and terminated early if the maximum absolute adjustment value fell below 5e-6.

$$f_i' = f_i\sqrt{\frac{\sum_{j \in A_i}\frac{c_{ij}}{E_{ij}}}{\sum_{j \in A_i}1}} \quad (21)$$

The ExpressKR algorithm used the matrix balancing algorithm described by Knight and Ruiz [4]. Because this approach requires a complete and symmetric matrix, values falling below the distance cutoff were given the value of zero. Learning was run until the residual fell below 1e-12. For the sample labeled “ExpressKR w/distance” in Figure 4 and Supplemental Figure 13, the estimated distance dependence signal was divided from the counts prior to learning.

For both Express approaches, after learning correction values for a chromosome μ_m was calculated and the square root of this mean was divided from the correction values (20).

1.2.10 HiC normalization - Binning

HiC data normalized using HiFive’s Binning algorithm were corrected using an adaptation of Yaffe and Tanay’s approach [5]. Each chromosome was handled independently (intra-chromosomal interactions only) and only interactions with an interaction distance greater than 500 Kb were used for calculations. Interactions were counted as binary values (one for a non-zero count, zero otherwise) rather than counts. The model used three features: fend length, GC content, and mappability. GC content was calculated as the percentage of guanine and cytosine in the 200 bp adjacent to the RE cut site and overlapping the fend. Mappability was defined as the percentage of uniquely mapping 30 bp fragments, starting every 10 bp, contained in the 500 bp adjacent to the RE cut site and overlapping the fend. Fend length and GC content were partitioned into 20 non-overlapping intervals such that each interval contained an

equal number of fends. Mappability was partitioned into 10 non-overlapping intervals spanning equal mappability ranges. The prior probability P_{prior} was calculated as the mean number of observations across all bins (22).

$$P_{prior} = \frac{\sum_{i,j \in A}^{c_{ij}>0} 1}{\sum_{i,j \in A} 1} \tag{22}$$

Seed values for each feature *correction P* for feature *k*, bins *a* and *b* were calculated as the mean number of observations in that bin combination divided by the prior probability (23).

$$P_{kab} = \frac{\sum_{i \in a} \sum_{j \in b}^{c_{ij} \in A} c_{ij}}{p_{prior} \sum_{i \in a} \sum_{j \in b}^{c_{ij} \in A} 1} \tag{23}$$

Mappability corrections were not optimized after seed values were calculated. Reads were modeled as arising from a binomial distribution with an expected value for an observation defined as the product of the prior probability and each feature correction of the total set of features *K* with bins *a* and *b* corresponding to the observation fends (24).

$$E_{ij} = P_{prior} \prod_{k \in K} P_{kab} \tag{24}$$

Learning was accomplished iteratively with each feature's correction values being optimized independently for each iteration. Learning was carried out for a maximum of 1000 iterations and was stopped early if the log-likelihood changed

by less than 1.0 for a given iteration. Optimization of correction values was performed using the Broyden-Fletcher-Goldfarb-Shanno algorithm.

For samples normalized for the pseudo-counts analysis, the specified number of counts was added to each bin, observed and possible, for each combination of ranges corresponding to that bin. For example, the bin for fend length interval one by fend length interval two had two times the pseudo count added, one for interactions in which the first fend fell in interval one and the second fend fell in interval two, and the second pseudo-count for interactions in which the first fend fell in interval two and the send fend fell in interval one.

1.3 Other method data normalizations

1.3.1 HiCPipe HiC normalization

Normalization of HiC data using HiCPipe v0.9 was carried out as described in Yaffe and Tanay [5]. To match HiFive's range, fends were only included within the range of each chromosome's first and last RE cut site. Read data were exported from HiFive for each dataset so all filtering based on read mapping and valid fend combinations were applied but fend coverage filtering was not performed on these data for HiCPipe normalization. Fends were instead filtered by mappability, marking fends with less than 50% mappability as invalid. Normalization was performed using a three-feature model, fragment length, GC content, and mappability. GC content and mappability were defined as described in *1.2.10 HiC normalization - binning*. The model used 20 bin ranges for fragment length and GC content, each partitioned to include equal numbers of valid

interactions. Mappability was partitioned into five bin ranges, each spanning a mappability range of 10%, from 50% to 100%. Fragment length and GC content corrections were optimized while mappability corrections were held constant.

1.3.2 HiCNorm HiC normalization

HiC normalization was performed using HiCNorm as described by Hu et al. [6] with the following changes. Code was adapted to python and implemented using the GLM generalized linear model function from the package 'statsmodels' instead of R. This was done for speed and memory considerations and was confirmed to give identical results. Data and features were as described in *1.3.1 HiCPipe HiC normalization*.

HiCNorm normalization for speed and memory usage used the original HiCNorm code rather than our adapted code. The only exception was for the binning of data, as there are no provided scripts for performing these operations. This was done in R to alleviate the need to load data from text files.

1.3.3 HiCLib HiC normalization

HiC data were normalized using the latest available version of HiCLib (obtained from the development repository on 04-20-15 [7]). Data were loaded directly from BAM mapped read files and filtered for PCR duplicates, a 500 bp insert size, and removing the lowest 0.5% of fends ranked by numbers of interactions. Data were normalized for 20 iterations.

1.3.4 Matrix-balancing HiC normalization

HiC data was normalized using a matrix balancing approach similar to that described by Rao et al. [8]. Data were loaded from HiFive, so all filtering described under *1.2.6 HiC filtering* was applied prior to normalization. Normalization was done on a per chromosome basis at fend level resolution using a python adaptation of the algorithm described by Knight and Ruiz [4]. Data were processed as binary observations rather than counts.

1.4 Data analysis

1.4.1 HiC probability model performance

In order to determine which probability model gave better results, we performed analysis on the mouse ESC datasets using a Poisson distribution as the underlying probability model in addition to the binomial model described above. Other than the cost and gradient equations, all other aspects of the analysis were identical to that described for the binomial probability normalization. Model performance was assessed using inter-dataset correlations (see Supplemental Methods: *HiC dataset correlations*).

Supplemental Figure 7 shows the dataset correlation differences between models, demonstrating an advantage of the binomial model across most interaction size ranges and bin sizes. The only cases where the Poisson distribution showed better correlation between datasets were mid-range interaction sizes for the 250 Kb and 1 Mb binned data and for the overall inter-chromosomal correlation for 1 Mb binned data. The gains in these cases were small compared to the improvements seen using the binomial model across all

other cases. Of particularly strong contrast was the effect that model choice had on data binned in smaller bins.

These results were particularly surprising to us, as the binomial model requires binary data rather than integers. Because HiC data are integer counts of observed reads, the Poisson distribution seems a good fit, but the noise or substructure appear to confound finding appropriate normalization corrections. In addition, counts data appear to converge with the binary representation of the data at longer ranges (Supplemental Fig. 6).

1.4.2 5C-HiC data correlations

The Pearson correlation between 5C data and HiC data for each 5C dataset was obtained across all regions for all log-transformed, non-zero, fragment-corrected 5C counts. The 5C data were compared to HiC data that was binned across both datasets (HindIII and NcoI) using the fragment partitions in the 5C datasets and dynamically binned using unbinned data combined from both datasets for bin expansion. Dynamic binning is a feature of HiFive whereby a minimum number of observed reads is required to consider a bin valid. Each bin is expanded in all directions, stopping each time the boundary encompasses new data from a set of expansion data (usually unbinned or binned at a finer scale). Each time an expansion bin is encountered the expanding bin is updated with the expansion bin's observation and expected values and checked to see if it meets the minimum count criterion. If so, expansion is halted. This allows bins in read-rich areas to retain higher resolution while bins in lower density regions of

reads are less subject to stochastic noise because more data points are contributing to their enrichment value.

1.4.3 HiC dataset correlations

Pearson correlations were found between normalized HiC datasets cut with different restriction enzymes. Data were binned at four resolutions, 10 Kb, 50 Kb, 250 Kb, and 1 Mb, for cis interactions and two resolutions, 250 Kb and 1 Mb, for trans interactions. For each binning size, overall correlations were found for log-transformed non-zero count normalized bin enrichments. Cis interactions were found for chromosomes 1-19 and X for mouse data and chromosomes 1-22 and X for human data. Trans interaction correlations were found only for inter-chromosomal interactions. In addition, correlations were calculated for interaction size ranges. The first range always spanned from 0 to five times the size of the resolution. The remaining nine bin ranges were partitioned into non-overlapping spans evenly covering log-distances up to 197.2 Mb for mouse and 249.3 Mb for human.

1.4.4 HiC normalization runtime comparison

An abbreviated dataset consisting of only interactions for which one end mapped to chromosome 1 from the mouse NcoI dataset was created, along with a corresponding RE cut site bed file and fend feature file. Each stage of analysis for each method was run separately and timing was accomplished using the ‘time’ Linux command to obtain wall-clock runtimes. Each run was repeated five

times and the median value was used for the analysis. All analyses were run on a single 2.294 GHz Quad-Core AMD Opteron Processor running Debian v3.2.4-1.

1.4.5 HiC normalization memory usage comparison

Memory usage was tested exactly as described under *1.4.3 HiC normalization runtime comparison* except using the maximum resident set size value (Supplemental Fig. 13). This analysis should be viewed with some skepticism. It is unclear how things such as memory garbage collection and transfer to the swap disk affect the reported RAM usage, so while these numbers may be relatively proportional, it is unclear how accurate some or all of the values are. Thus we urge caution in interpreting the memory usage data. That being said, it appears that HiCPipe is the most memory efficient, followed by HiFive (all but the probability algorithm) and then HiCLib. HiFive's probability algorithm appeared to use about an order of magnitude more RAM than other methods, although this is unsurprising given the modeling of all interactions. The most memory-intensive was HiCNorm, using twice as much RAM at its peak than HiFive probability.

2 Supplemental References

1. Barrett T, Troup DB, Wilhite SE, Ledoux P, Rudnev D, Evangelista C, Kim IF, Soboleva A, Tomashevsky M, Marshall KA, et al: **NCBI GEO: archive for high-throughput functional genomic data.** *Nucleic Acids Res* 2009, **37:**D885-890.

2. Langmead B, Trapnell C, Pop M, Salzberg SL: **Ultrafast and memory-efficient alignment of short DNA sequences to the human genome.** *Genome Biol* 2009, **10:**R25.

3. Lieberman-Aiden E, van Berkum NL, Williams L, Imakaev M, Ragoczy T, Telling A, Amit I, Lajoie BR, Sabo PJ, Dorschner MO, et al: **Comprehensive mapping of long-range interactions reveals folding principles of the human genome.** *Science* 2009, **326:**289-293.

4. Knight PA, Ruiz D: **A fast algorithm for matrix balancing.** *IMA Journal of Numerical Analysis* 2012**:**drs019.

5. Yaffe E, Tanay A: **Probabilistic modeling of Hi-C contact maps eliminates systematic biases to characterize global chromosomal architecture.** *Nat Genet* 2011, **43:**1059-1065.

6. Hu M, Deng K, Selvaraj S, Qin Z, Ren B, Liu JS: **HiCNorm: removing biases in Hi-C data via Poisson regression.** *Bioinformatics* 2012, **28:**3131-3133.

7. Imakaev M, Fudenberg G, McCord RP, Naumova N, Goloborodko A, Lajoie BR, Dekker J, Mirny LA: **Iterative correction of Hi-C data reveals hallmarks of chromosome organization.** *Nat Methods* 2012, **9:**999-1003.

8. Rao SS, Huntley MH, Durand NC, Stamenova EK, Bochkov ID, Robinson JT, Sanborn AL, Machol I, Omer AD, Lander ES, Aiden EL: **A 3D Map of**

the Human Genome at Kilobase Resolution Reveals Principles of Chromatin Looping. *Cell* 2014, **159:**1665-1680.

9. Nora EP, Lajoie BR, Schulz EG, Giorgetti L, Okamoto I, Servant N, Piolot T, van Berkum NL, Meisig J, Sedat J, et al: **Spatial partitioning of the regulatory landscape of the X-inactivation centre.** *Nature* 2012, **485:**381-385.
10. Phillips-Cremins JE, Sauria ME, Sanyal A, Gerasimova TI, Lajoie BR, Bell JS, Ong CT, Hookway TA, Guo C, Sun Y, et al: **Architectural protein subclasses shape 3D organization of genomes during lineage commitment.** *Cell* 2013, **153:**1281-1295.
11. Dixon JR, Selvaraj S, Yue F, Kim A, Li Y, Shen Y, Hu M, Liu JS, Ren B: **Topological domains in mammalian genomes identified by analysis of chromatin interactions.** *Nature* 2012, **485:**376-380.
12. Selvaraj S, J RD, Bansal V, Ren B: **Whole-genome haplotype reconstruction using proximity-ligation and shotgun sequencing.** *Nat Biotechnol* 2013, **31:**1111-1118.

3 Supplemental Table and Figures

Supplemental Table 1 – Sources of 5C and HiC datasets.

Sample	Rep	Species	Cell Type	Data Type	Reference	GEO ID
male ES E14	1	Mouse	male mES	5C	Nora et al. [9]	GSM873934
male ES E14	2	Mouse	male mES	5C	Nora et al. [9]	GSM873935
ES	1	Mouse	V6.5 mES	5C	Phillips-Cremins et al. [10]	GSM883649
ES	2	Mouse	V6.5 mES	5C	Phillips-Cremins et al. [10]	GSM883650
ES HindIII	1	Mouse	J1 mES	HiC	Dixon et al. [11]	GSM862720
ES HindIII	2	Mouse	J1 mES	HiC	Dixon et al. [11]	GSM862721
ES NcoI	1	Mouse	J1 mES	HiC	Dixon et al. [11]	GSM862722
HindIII	1	Human	GM12878	HiC	Selvaraj et al. [12]	GSM1181867
HindIII	2	Human	GM12878	HiC	Selvaraj et al. [12]	GSM1181868
MboI	1	Human	GM12878	HiC	Rao et al. [8]	GSM1551550 – GSM1551567
MboI	2	Human	GM12878	HiC	Rao et al. [8]	GSM1551568 – GSM1551578

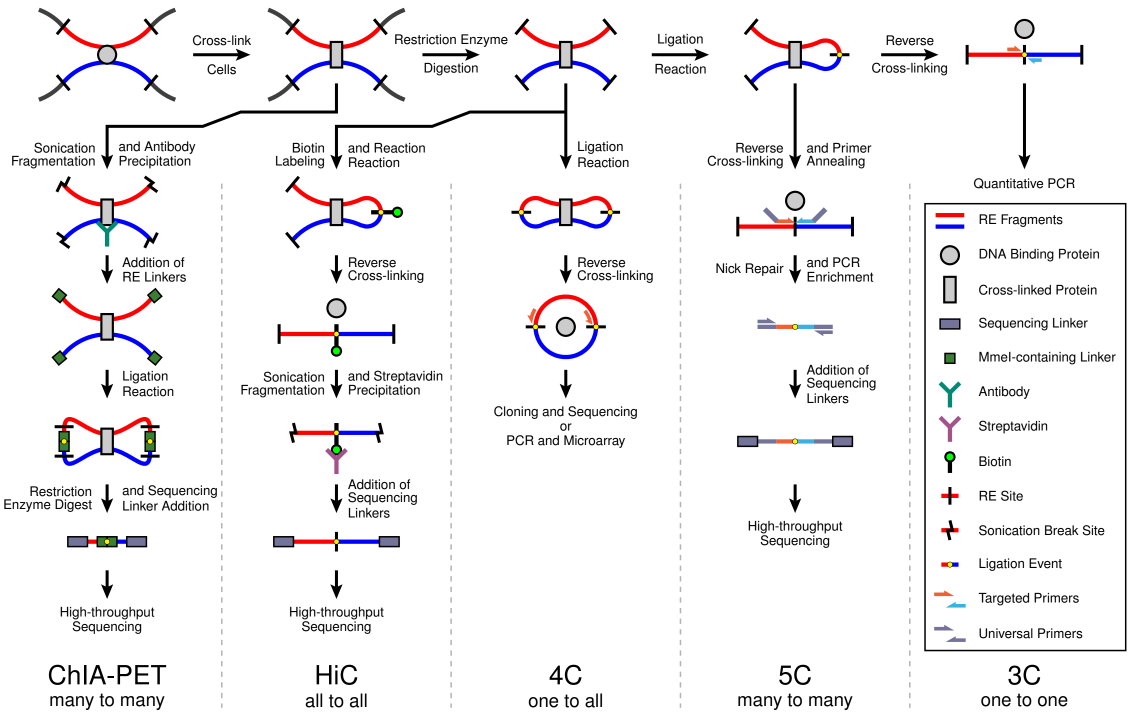

Supplemental Figure 1 - Proximity-mediated ligation assays.

A survey of strategies for assaying chromatin interactions.

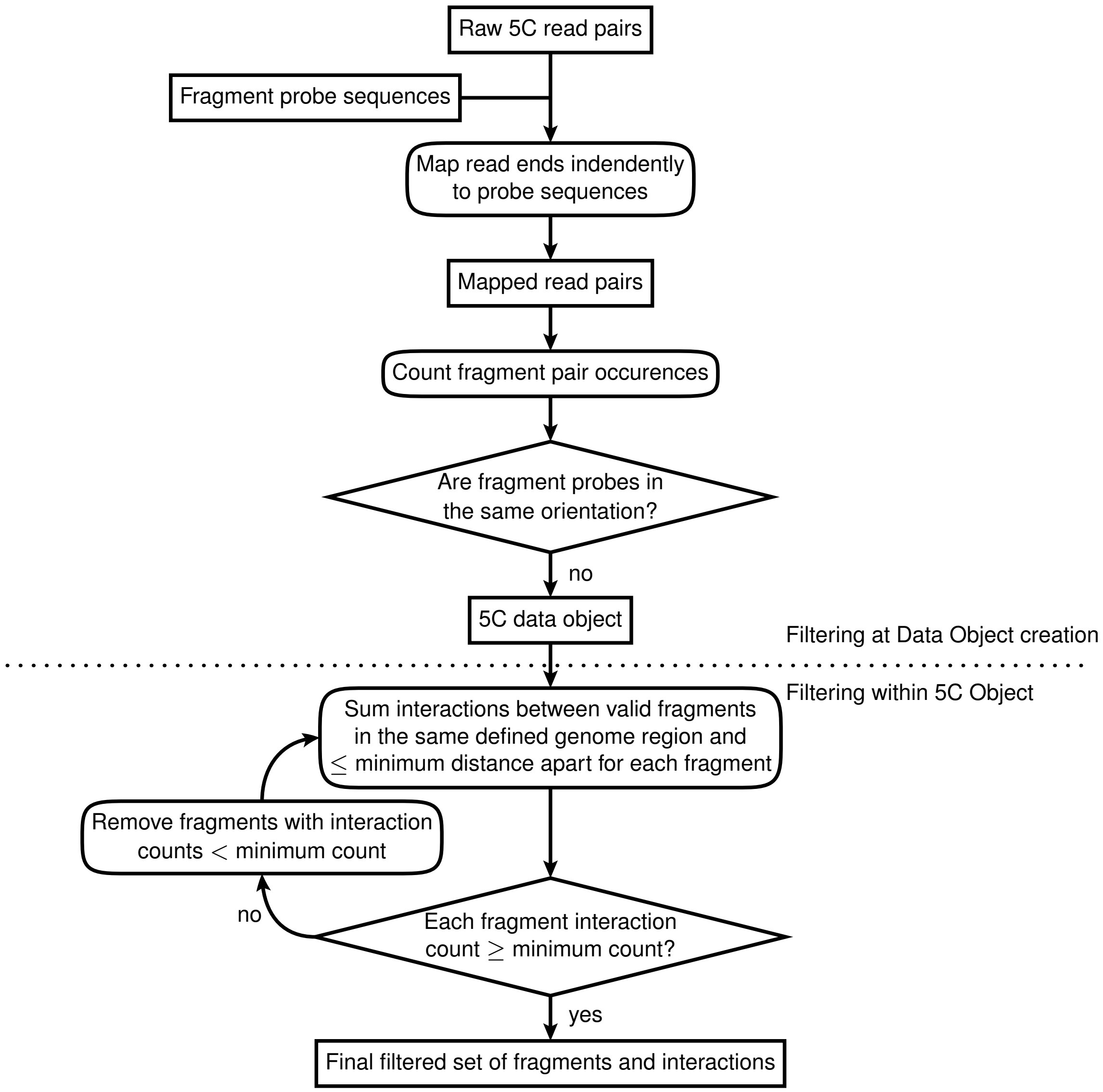

Supplemental Figure 2 – 5C filtering scheme.

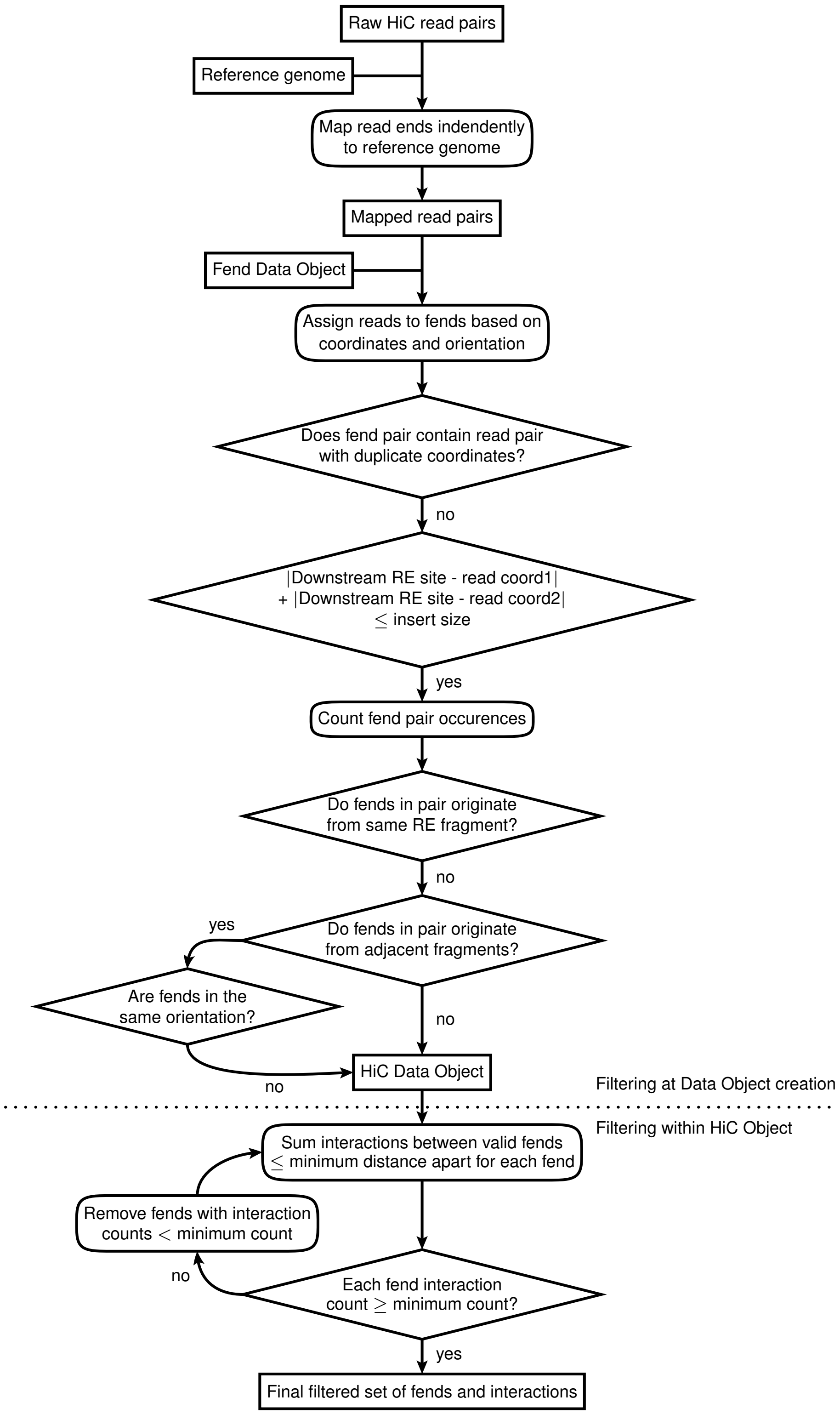

Supplemental Figure 3 – HiC filtering scheme.

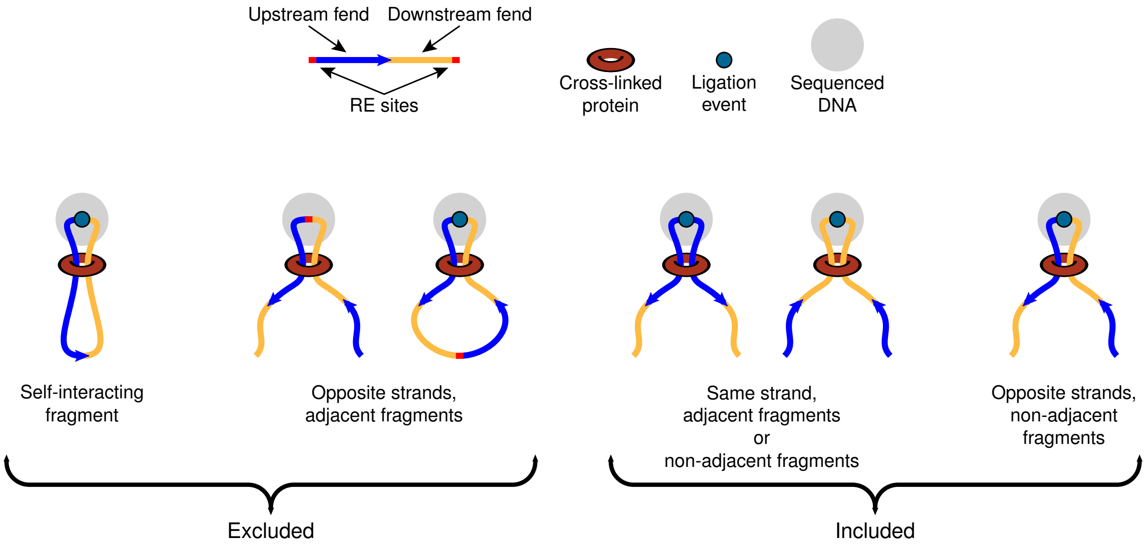

Supplemental Figure 4 – HiC read pairings.

Schematic illustrating the arrangements leading to HiC read pairs and their inclusion or exclusion from HiFive analysis.

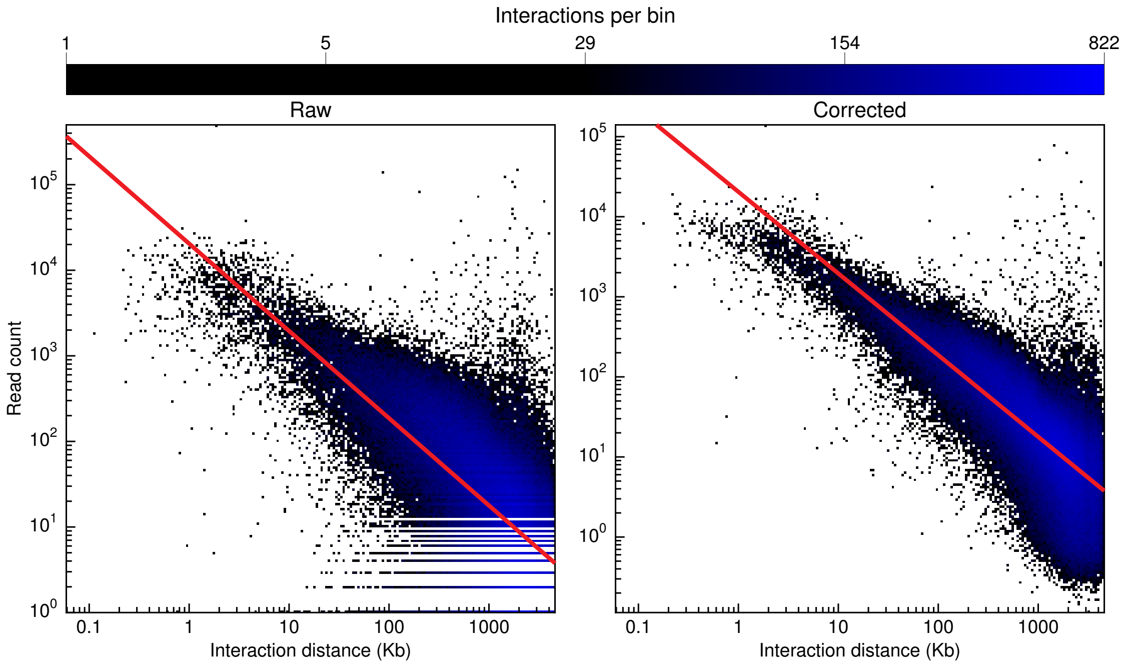

Supplemental Figure 5 – 5C distance function.

All non-zero interaction counts for mouse ES cells, replicate one before and after fragment corrections are applied. Interactions were binned in a 200 by 200 grid for display. The red line shows the best-fit linear regression of interaction log-transformed counts as a function of inter-fragment log-transformed distances.

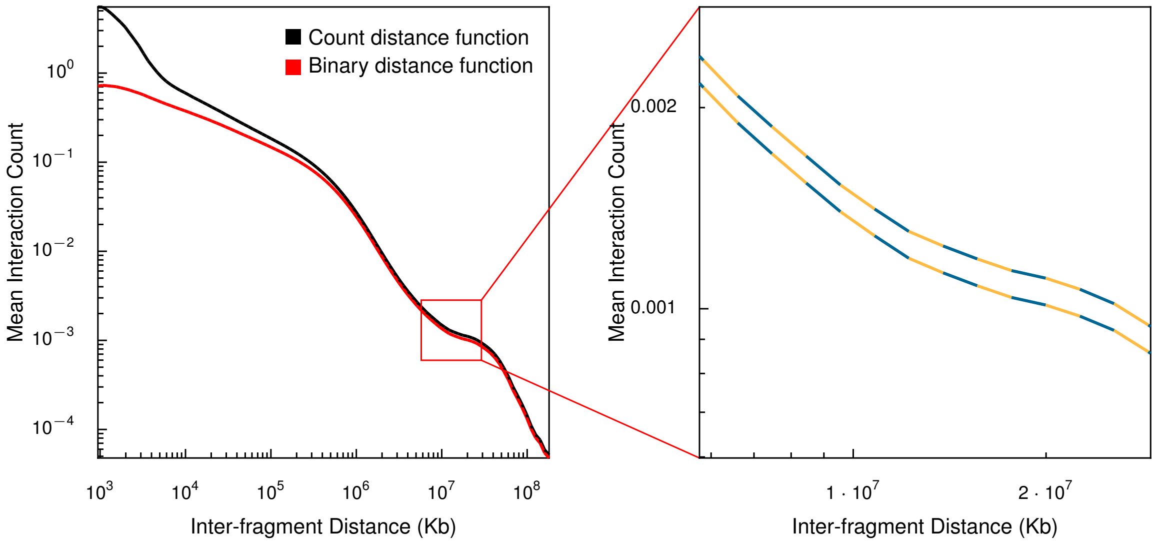

Supplemental Figure 6 – HiC distance function.

The piecewise linear approximation of the distance dependence relationship between HiC counts and interaction distances. The function calculated with numbers of reads shown in black while the function calculated using a binary indicator of observed/unobserved is shown in red. The graph to the right shows individual line segment approximations in alternating colors corresponding to the red box.

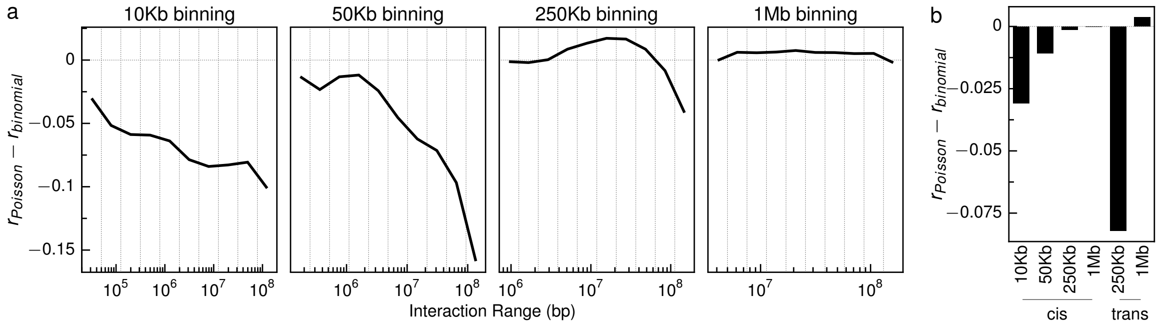

Supplemental Figure 7 – HiC probability algorithm model comparison.

Differences in inter-dataset correlations between data normalized using a Poisson distribution for modeling noise and data normalized using a binomial distribution for modeling noise. a) Correlations across mutually-exclusive interaction size ranges for data binned at four different resolutions. b) Correlation differences for entire set of intra- (cis) or inter-chromosomal (trans) interactions.

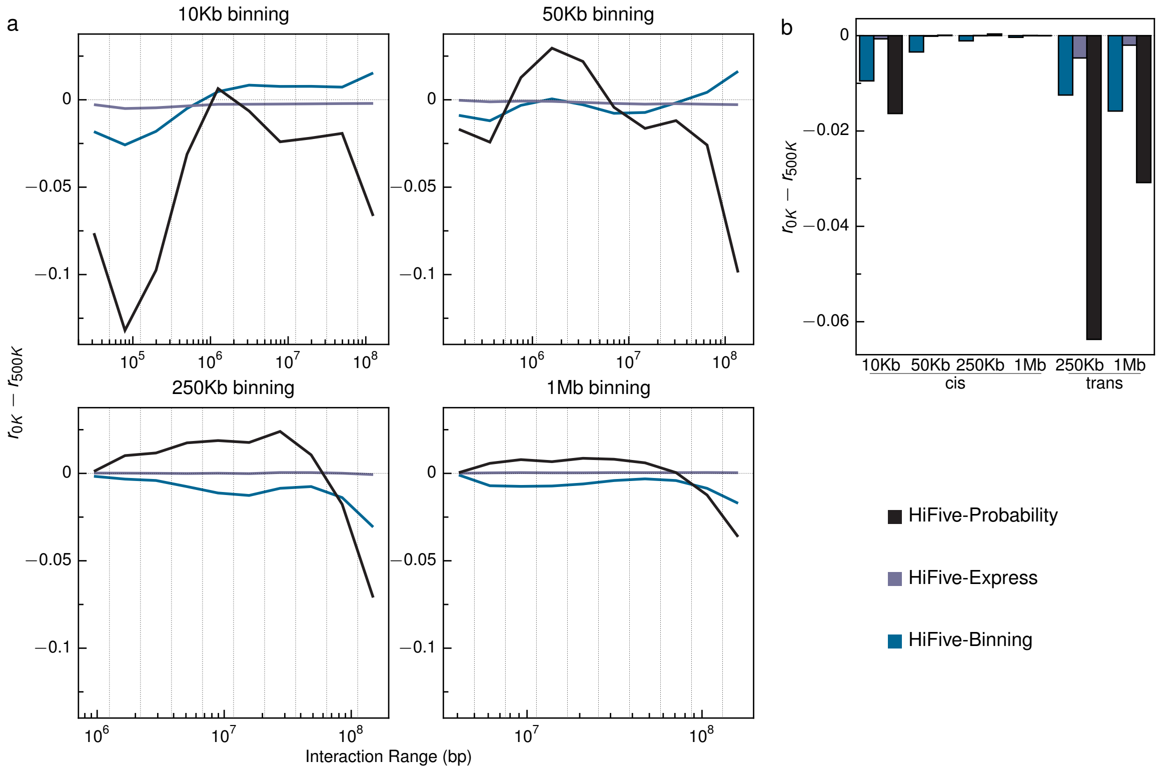

Supplemental Figure 8 – HiC algorithm distance cutoff comparison.

Differences in inter-dataset correlations between data normalized using all interaction size ranges and data normalized using only interactions larger than 500 Kb. a) Correlations across mutually-exclusive interaction size ranges for data binned at four different resolutions. b) Correlation differences for entire set of intra- (cis) or inter-chromosomal (trans) interactions.

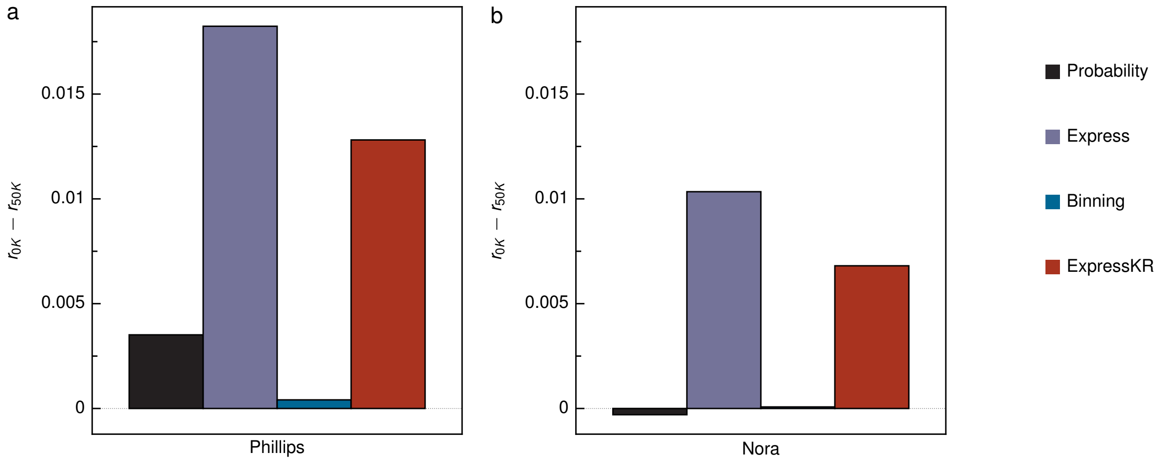

Supplemental Figure 9 – 5C algorithm distance cutoff comparison.

Differences in correlation of 5C-HiC data between 5C data normalized using all interaction sizes and data normalized using only interactions greater than 50 Kb.

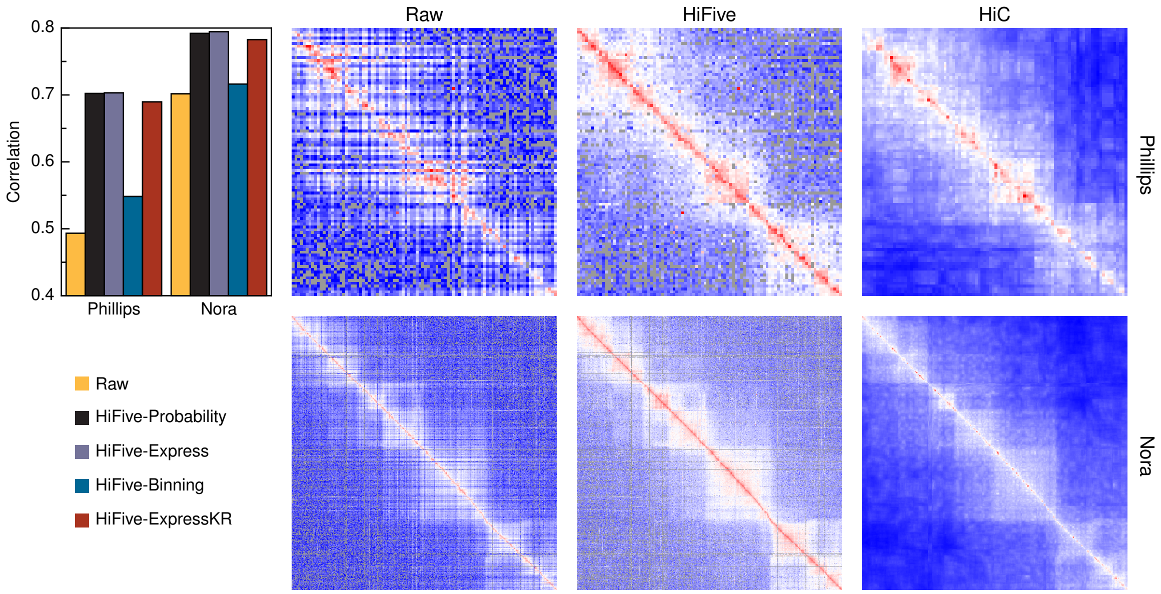

Supplemental Figure 10 – 5C-HiCPipe analysis performance.

HiFive normalization of 5C data and its correlation to corresponding HiC data normalized using HiCPipe. a) Correlation of 5C data (intra-regional only) with the same cell type and bin-coordinates in HiC data for two different datasets and using each of HiFive's algorithms. b) Heatmaps for a select region from each dataset, un-normalized, normalized using HiFive's probability algorithm, and the corresponding HiC data, normalized using HiCPipe and dynamically binned.

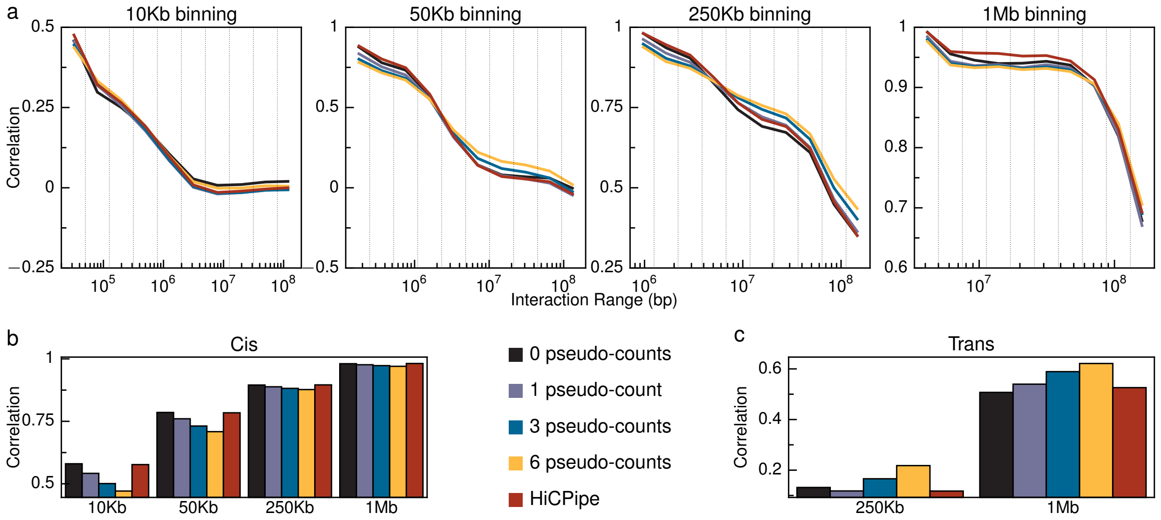

Supplemental Figure 11 - Effects of pseudo-counts.

Mouse data were normalized using HiFive-Express with 0, 1, 3, or 6 pseudo-counts added to each fend-feature bin in the binning model prior to normalization. Results for HiCPipe are also shown for comparison. a) Interactions were broken down into ten groups of non-overlapping cis interaction ranges for four resolution levels. b) Overall cis interaction correlations for each pseudo-count level are shown across all bin size ranges. c) Overall trans interaction correlations for the larger two bin sizes. Trans interactions were not considered for bins smaller than 250 Kb.

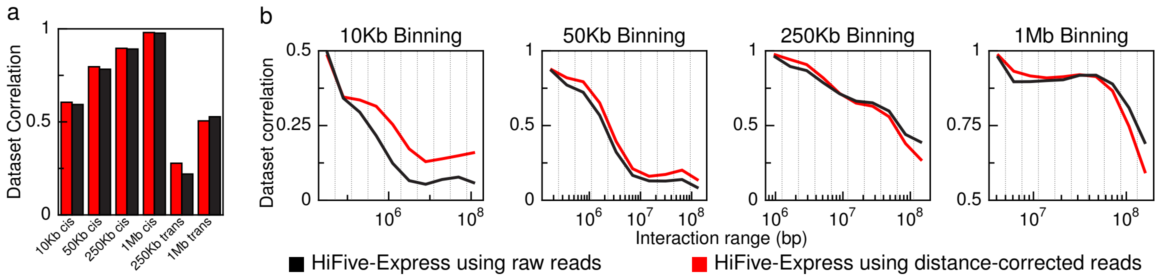

Supplemental Figure 12 - Effects of distance dependence on normalization. Data analyzed using HiFive-express either with or without the estimated distance-dependence signal removed prior to normalization. Interactions normalized using raw counts are shown in black while interactions that were adjusted for the predicted distance-dependent signal prior to normalization are shown in red. a) Correlation of datasets across all interaction ranges for different binning resolutions including interactions from intra (cis) or inter-chromosomal (trans) interactions. b) Correlations between mouse HiC datasets produced using two different restriction enzyme, binned at four resolutions and subdivided into a series of ten interaction size ranges.

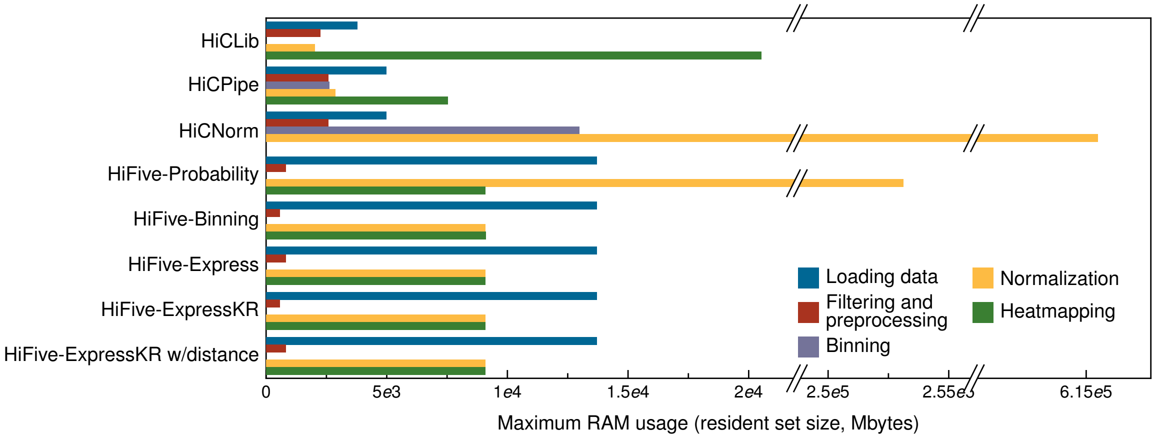

Supplemental Figure 13 - Maximum RAM usage by HiC analysis methods.

Each stage of processing, from loading data to creating a final heatmap, was profiled to determine peak RAM usage. Values may not reflect actual utilization but rather cumulative allocation. Note that because of two extreme values, the graph includes multiple splits.
