# Supplementary material for: HiFive: a tool suite for easy and efficient HiC and 5C data analysis
Source: Genome Biol. 2015 Oct 24;16:237. doi: 10.1186/s13059-015-0806-y (PMC5410870; doi:10.1186/s13059-015-0806-y)
Supplement: Supplementary file 2 — A tar archive containing the HiFive software library. (BZ2 578 kb) [file 13059_2015_806_MOESM2_ESM.bz2 › hifive-1.1.3/doc/_templates/layout.html]

{% extends "!layout.html" %}
{% block rootrellink %}- home|
- search|
{% endblock %}
{% block relbar1 %}{{ super() }}
{% endblock %}
{# put the sidebar before the body #}
{% block sidebar1 %}{{ sidebar() }}{% endblock %}
{% block sidebar2 %}{% endblock %}
