# Supplementary figures and images for: HiFive: a tool suite for easy and efficient HiC and 5C data analysis
Source: Genome Biol. 2015 Oct 24;16:237. doi: 10.1186/s13059-015-0806-y (PMC5410870; doi:10.1186/s13059-015-0806-y)

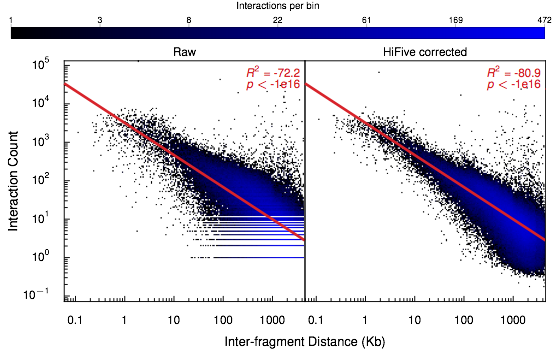

Supplement: Supplementary file 2 — A tar archive containing the HiFive software library. (BZ2 578 kb) [file 13059_2015_806_MOESM2_ESM.bz2 › hifive-1.1.3/doc/_static/5c_distance.png]

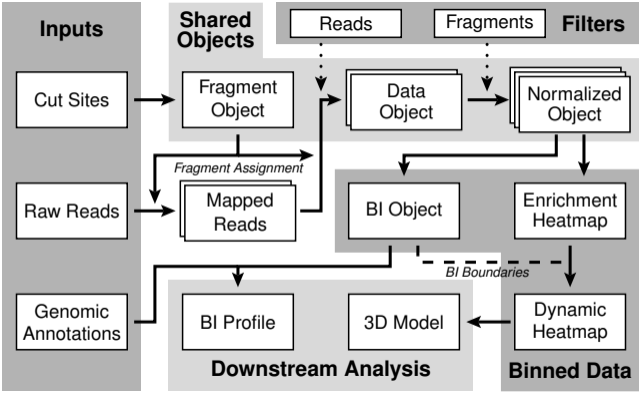

Supplement: Supplementary file 2 — A tar archive containing the HiFive software library. (BZ2 578 kb) [file 13059_2015_806_MOESM2_ESM.bz2 › hifive-1.1.3/doc/_static/flowchart.pdf]

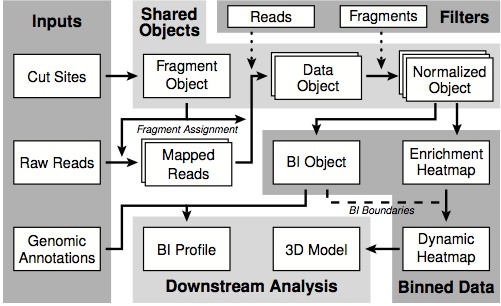

Supplement: Supplementary file 2 — A tar archive containing the HiFive software library. (BZ2 578 kb) [file 13059_2015_806_MOESM2_ESM.bz2 › hifive-1.1.3/doc/_static/flowchart.png]

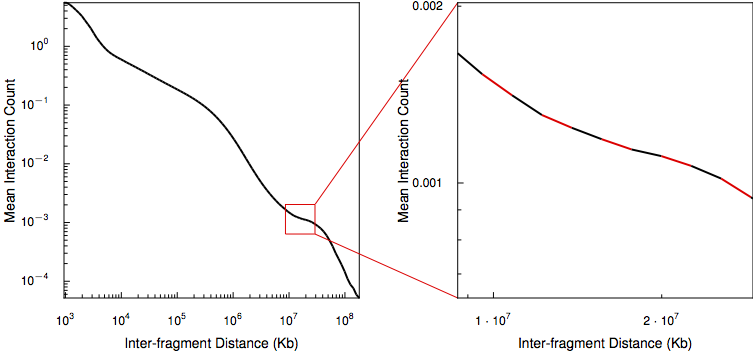

Supplement: Supplementary file 2 — A tar archive containing the HiFive software library. (BZ2 578 kb) [file 13059_2015_806_MOESM2_ESM.bz2 › hifive-1.1.3/doc/_static/hic_distance.png]

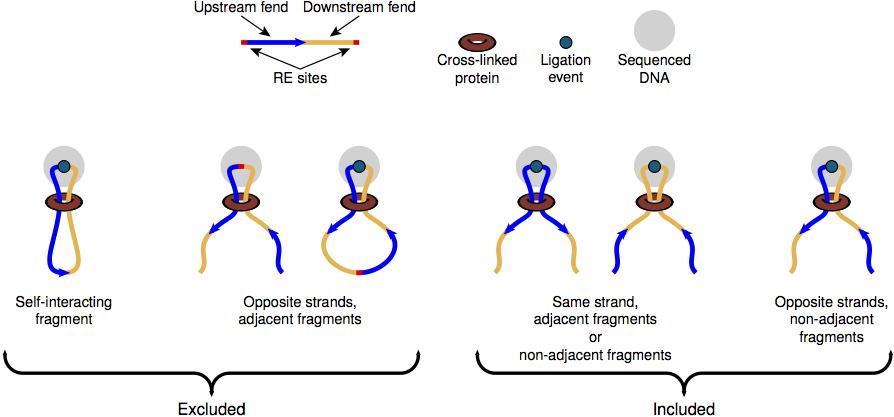

Supplement: Supplementary file 2 — A tar archive containing the HiFive software library. (BZ2 578 kb) [file 13059_2015_806_MOESM2_ESM.bz2 › hifive-1.1.3/doc/_static/hic_filter.png]
