# Supplementary material for: HiFive: a tool suite for easy and efficient HiC and 5C data analysis
Source: Genome Biol. 2015 Oct 24;16:237. doi: 10.1186/s13059-015-0806-y (PMC5410870; doi:10.1186/s13059-015-0806-y)
Supplement: Supplementary file 4 — The software documentation. (PDF 530 kb) [file 13059_2015_806_MOESM4_ESM.pdf]

HiFive Documentation

Release 1.1.3

Michael Sauria

September 11, 2015

1 Introduction 3
1.1 HiFive Overview 3
1.2 Organization of the HiFive package 4
1.3 Release History 5

2 Getting started 7
2.1 Installing HiFive 7
2.2 Installing Documentation 8

3 Restriction Fragment Handling 9
3.1 Loading 5C Fragments 9
3.2 Loading HiC Fends 9

4 Loading Data 11
4.1 Loading 5C data 11
4.2 Loading HiC Data 11

5 Filtering Data 13
5.1 5C Filtering 13
5.2 HiC Filtering 13

6 Distance-Dependence Estimation 15
6.1 5C Distance-Dependence Estimation 15
6.2 HiC Distance-Dependence Estimation 16

7 Normalizing Data 19
7.1 Probability Algorithm 19
7.2 Express Algorithm 19
7.3 Binning Algorithm 20
7.4 Chaining Normalization Approaches 20

8 Binning Strategies 21
8.1 The ‘upper’ format 21
8.2 The ‘full’ format 21
8.3 The HiC ‘compact’ format 21
8.4 The 5C ‘compact’ format 21
8.5 Dynamic Binning 22

9 Multi-Resolution Heatmap 23

9.1 MRH Approach 23
9.2 Standalone MRH program 23
9.3 File specs 24

10 Using HiFive **27**
10.1 5C Subcommands 28
10.2 5C Options 30
10.3 HiC Subcommands 33
10.4 HiC Options 36

11 Tutorial for 5C Classes **43**
11.1 Creating a `Fragment` object 43
11.2 Creating a `FiveCData` object 44
11.3 Creating a `FiveC` project object 44
11.4 Filter 5C fragments 45
11.5 Find 5C distance function 45
11.6 Learn 5C normalization parameters 45
11.7 Generating a heatmap 47
11.8 Accessing heatmap data 47
11.9 Plotting a heatmap 48

12 Tutorial for HiC Classes **49**
12.1 Creating a `Fend` object 49
12.2 Creating a `HiCData` object 49
12.3 Creating a `HiC` project object 50
12.4 Filter HiC fends 51
12.5 Find HiC distance function 51
12.6 Learn HiC normalization parameters 52
12.7 Generating a heatmap 54
12.8 Accessing heatmap data 55
12.9 Plotting a heatmap 55
12.10 Creating a multi-resolution heatmap 55

13 HiFive's API **57**
13.1 The Fragment class 57
13.2 The Fend class 58
13.3 The FiveCData class 60
13.4 The HiCData class 62
13.5 The FiveC class 66
13.6 The HiC class 75
13.7 The fivec_binning module 87
13.8 The hic_binning module 93
13.9 The plotting module 100

14 Release History **107**
14.1 1.1 (2015-08-26) 107
14.2 1.0.3 (2015-04-23) 107
14.3 1.0.2 (2015-04-16) 107

15 Indices and tables **109**

Python Module Index **111**

Contents:

CHAPTER 1

Introduction

HiFive is a Python package for normalization and analysis of chromatin structural data produced using either the 5C of HiC assay. The aim of HiFive is to create an efficient framework for handling the large amounts of data associated chromatin interaction studies and provide an easy interface for working with and normalizing these data. There are two ways of utilizing HiFive, either through its binary executable or by using its set of classes and functions for more flexibility in analysis. In order to use HiFive, only two types of information are required. First, you need a record of restriction enzyme fragments in an appropriate format (see *hifive.fragment.Fragment* or *hifive.fend.Fend*) for either the subset of targeted fragments or the whole genome for 5C and HiC, respectively. Second, you need 5C or HiC chromatin interaction data, either in a mapped BAM format or in an appropriate post-processed format (see *hifive.fragment.Fragment* or *hifive.fend.Fend*). HiFive will handle everything else. In addition, because of the large memory and time requirements, HiFive uses modularity to avoid repeating processes such as loading data. This allows easy exploration of analysis parameters at a faster rate.

1.1 HiFive Overview

```
hifive [-h] [--version]
            {fragments,5c-data,5c-project,5c-normalize,5c-complete,
            5c-heatmap,5c-interval,combine-5c-replicates,
            fends,hic-data,hic-project,hic-normalize,hic-complete,
            hic-heatmap,hic-interval,combine-hic-replicates}
```

There are eight major functions for both 5C and HiC data types available as subcommands in HiFive.

1.1.1 5C

fragments Create a fragment file from a BED file containing targeted RE fragment data.

5c-data Create a data file from mapped BAM or fragment-pair counts files.

5c-project Create a project file, filter fragments, and estimate distance-dependence.

5c-normalize Find correction parameter values using one of the available algorithms (see normalization).

5c-complete Perform all of the steps of the subcommands fragments, 5c-data, 5c-project, and 5c-normalization in one command.

5c-heatmap Using an already created 5C project, generate an HDF5-formatted heatmap file and optional image.

5c-interval Using an already created 5C project, generate a tabular genomic-interval file for a specified region and optional image.

5c-combine-replicates Combine multiple 5C data files into a single file without needing to reload the data.

1.1.2 HiC

fends Create a fend file from either a BED or HiCPipe-style fend file containing RE fragment data.

hic-data Create a data file from mapped BAM, MAT, or paired coordinate text files.

hic-project Create a project file, filter fends, and estimate distance-dependence.

hic-normalize Find correction parameter values using one of the available algorithms (see normalization).

hic-complete Perform all of the steps of the subcommands fends, hic-data, hic-project, and hic-normalization in one command.

hic-heatmap Using an already created HiC project, generate an HDF5-formatted heatmap file and optional image.

hic-interval Using an already created HiC project, generate a tabular genomic-interval file for a specified region and optional image.

hic-combine-replicates Combine multiple HiC data files into a single file without needing to reload the data.

A more detailed explanation of the subcommands and all of the command options is here.

1.2 Organization of the HiFive package

The `HiFive` package is split into several modules, each serving a specific purpose.

Functionality	Module
Restriction enzyme information	*Fragment* / *Fend*
Read counts and orientations	*FiveCData* / *HiCData*
Model parameters and filtering	*FiveC* / *HiC*
Plotting functions	*plotting*

The classes *Fragment*, *Fend*, *FiveCData*, *HiCData*, *FiveC*, and *HiC* are all available from the top level of the `HiFive` namespace and can be imported using:

```
from hifive import *
```

at the beginning of the Python program. However, in order to prevent namespace pollution, you may also simply use:

```
import hifive
```

HiFive is organized into a hierarchy of data structures. Each structure represents a set of data that may be shared with any number of structures higher in the hierarchy, thus eliminating redundancy of information. For example, a *Fragment* object which contains information about the fragments being interrogated in a 5C experiment can be used for all replicates and conditions that use the same primer scheme. Likewise, a *HiCData* object which contains all of the mapped read information for a specific HiC experiment can be used for multiple analyses with different parameter values. This helps reduce the space these data occupy as well as reduce the time to run multiple analyses since each object need only be created once.

1.3 Release History

- *1.1 (2015-08-26)*
- *1.0.3 (2015-04-23)*
- *1.0.2 (2015-04-16)*

CHAPTER 2

Getting started

The `HiFive` binary requires Python 2.7. However, the library can be used by Python 2.6 and 2.7. To check your version, run:

```
> python -V
```

2.1 Installing HiFive

The easiest way to get `HiFive` is using pip:

```
> pip install hifive
```

`HiFive` can also be obtained from HiFive using the following command:

```
> git clone https://github.com/bxlab/hifive.git
```

or alternatively, download a snapshot of the repository using the following commands:

```
> wget https://github.com/bxlab/hifive/tarball/1.0
> tar -xf hifive_v1.0.tar
```

`HiFive` depends on a few packages and has several others that extend its functionality.

2.1.1 Required Packages

- Scipy
- Numpy
- h5py

2.1.2 Recommended Packages

- Sphinx for generating local documentation
- Pysam for reading BAM files
- Pyx for generating PDF images
- PIL for generating bitmap images
- mpi4py for utilizing MPI capabilities of several HiC functions

- mlpy for modeling 3D structure

For a more traditional Python installation simply setup a virtualevn for `HiFive` (this example creates one in .venv in your home directory) and then install the required packages and HiFive.

```
> virtualenv ~/.venv; source ~/.venv/bin/activate
```

To install `HiFive`, simply enter the directory that the repository was cloned or downloaded to and use the following command:

```
> python setup.py install
```

If you wish to install `HiFive` in a location other than the default, you can use the prefix option:

```
> python setup.py install --prefix=/your/desired/path
```

2.2 Installing Documentation

In order to build HiFive's documentation locally, you need to execute the following command:

```
> cd doc
> make html
```

This will create the documentation suitable for viewing with a web browser. In order to create a pdf version of the documentation, simply run the following:

```
> cd doc
> make latexpdf
```

Restriction Fragment Handling

HiFive handles all data at the level of the restriction fragment via the *Fragment* and *Fend* classes. In the case of 5C data, this means the set of fragments that are specifically targeted by primers, whereas for HiC data, each restriction fragment is treated as two separate units, one for each half of the fragment. The reason for this is that each fragment end (fend) is associated with its own characteristics such as GC content and mappability. Further, because there is an inverse relationship between fragment length and overall interaction signal strength, successful ligation and sequencing is biased towards interaction sites (and therefore cross-linking sites) that occur closer to the end being ligated. Effectively this creates two different populations of interactions, those associated with each half of a restriction fragment.

All normalization and binning are accomplished from the fragment or fend level for 5C and HiC data, respectively. This means that once normalization is complete, it can be used for any level of resolution.

3.1 Loading 5C Fragments

Fragments associated with 5C data are loaded from a BED file that contains the chromosome, start and ending coordinates, name, and strand for each primer-targeted restriction fragment from the 5C experiment. Additionally, a FASTA file containing the primer sequences can also be provided to associate the GC content of each primer with its genome position data (used in HiFive's *Binning Algorithm*). To reduce the storage space and processing time, only fragment data associated with primer targets are used in HiFive's 5C Fragment objects, meaning that a different Fragment object is needed for each experimental design, but experiments sharing the same targeted fragments may share a Fragment object file.

3.2 Loading HiC Fends

Fends associated with HiC data can be loaded from either a BED file or a HiCPipe-compatible tabular fend file. If using a BED file, the file should contain either the chromosome, start, and stop position of each restriction fragment or the chromosome, start, and stop position of the restriction enzyme recognition sites for the target genome. HiFive will infer which type of BED file is given based on the coordinate intervals. If a HiCPipe-compatible fend file is given, it should contain a header line, fend, fragment, chromosome, coordinate and fragment length information for each fend in the target genome.

```
fend    frag    chr    coord      valid    frag_len
1       1       1      3002506    1        3372
2       1       1      3005877    1        3372
3       2       1      3005878    1        389
4       2       1      3006266    1        389
```

Note: HiCPipe-style fend files are 1-indexed, meaning that the first fragment and first fend both are labeled with a 1. This convention is used in HiFive only for these files to maintain compatibility with HiCPipe files.

The header line should contain the exact labels as seen above since HiFive uses them to determine which columns contain what information. In addition to the above characteristics, the tabular fend file may also contain the columns 'frag_gc' and 'map_score'. These fend characteristic values are used in HiFive's *Binning Algorithm*, although are not needed for either probability or express normalization.

CHAPTER 4

Loading Data

HiFive data is handled using the *FiveCData* and HiCData classes.

4.1 Loading 5C data

HiFive can load 5C data from one of two source file types.

4.1.1 BAM Files

When loading 5C data from BAM files, they should always come in pairs, one for each end of the paired-end reads. HiFive can load any number of pairs of BAM files, such as when multiple sequencing lanes have been run for a single replicate. These files do not need to be indexed or sorted. All sequence names that these files were mapped against should exactly match the primer names in the BED file used to construct the Fragment object.

4.1.2 Count Files

Counts files are tabular text files containing pairs of primer names and a count of the number of observed occurrences of that pairing.

```
5c_for_primer1   5c_rev_primer2    10
5c_for_primer1   5c_rev_primer4    3
5c_for_primer3   5c_rev_primer4    18
```

4.2 Loading HiC Data

HiFive can load HiC data from three different types of source files.

4.2.1 BAM Files

When loading HiC data from BAM files, they should always come in pairs, one for each end of the paired-end reads. HiFive can load any number of pairs of BAM files, such as when multiple sequencing lanes have been run for a single replicate. These files do not need to be indexed or sorted. For faster loading, especially with very large numbers of reads, it is helpful to parse out single-mapped reads to reduce the number of reads that HiFive needs to traverse in reading the BAM files.

4.2.2 RAW Files

RAW files are tabular text files containing pairs of read coordinates from mapped reads containing the chromosome, coordinate, and strand for each read end. HiFive can load any number of RAW files into a single HiC Data object.

```
chr1    30002023    +    chr3    4020235     -
chr5    9326220     -    chr1    3576222     +
chr8    1295363     +    chr6    11040321    +
```

4.2.3 MAT Files

MAT files are in a tabular text format previously defined for HiCPipe. This format consists of a pair of fend indices and a count of observed occurrences of that pairing. These indices must match those associated with the Fend object used when loading the data. Thus it is wise when using this format to also create the Fend object from a HiCPipe-style fend file to ensure accurate fend-count association.

```
fend1    fend2    count
1        4        10
1        10       5
1        13       1
```

Note: In order to maintain compatibility with HiCPipe, both tabular fend files and MAT files are 1-indexed, rather than the standard 0-indexed used everywhere else with HiFive.

CHAPTER 5

Filtering Data

5.1 5C Filtering

HiFive filters reads from 5C experiments in two stages.

1. When a data object is created and data are loaded, only valid reads are read in. In 5C, this means only reads that represent an interaction between a forward-probed restriction fragment and a reverse-probed restriction fragment.
2. HiFive calls a function (*filter_fragments*) after a project is created and loaded with data that iteratively removes reads to produce a set of fragments that meet a minimum threshold of interactions with other valid fragments within the same region. This filtering is done using the number of interacting fragments rather than reads to account for the fact that fragment biases can produce wildly different numbers of interactions between fragment pairs. This filtering can also be limited to interactions falling within some range limitation of interaction distance. This can be used to ensure that fragments have sufficient reads for learning correction parameters when distance range constraints are used for normalization.

5.2 HiC Filtering

HiFive filters reads from HiC experiments in two stages.

1. When a data object is created and data are loaded, only valid reads are read in. In HiC, this means that reads with identical mapping coordinates at both ends are assumed to be PCR duplicates and only one read is counted. Reads are also filtered by the insert size as determined by the sum of the distances from the mapped positions of both ends to the nearest downstream target restriction site for each. In addition, because of the possibility of incomplete restriction enzyme digestion and fragment circularization, reads with ends mapping to the same fragment and reads with ends mapping to adjacent fragments on opposite strands are also excluded from the data object.

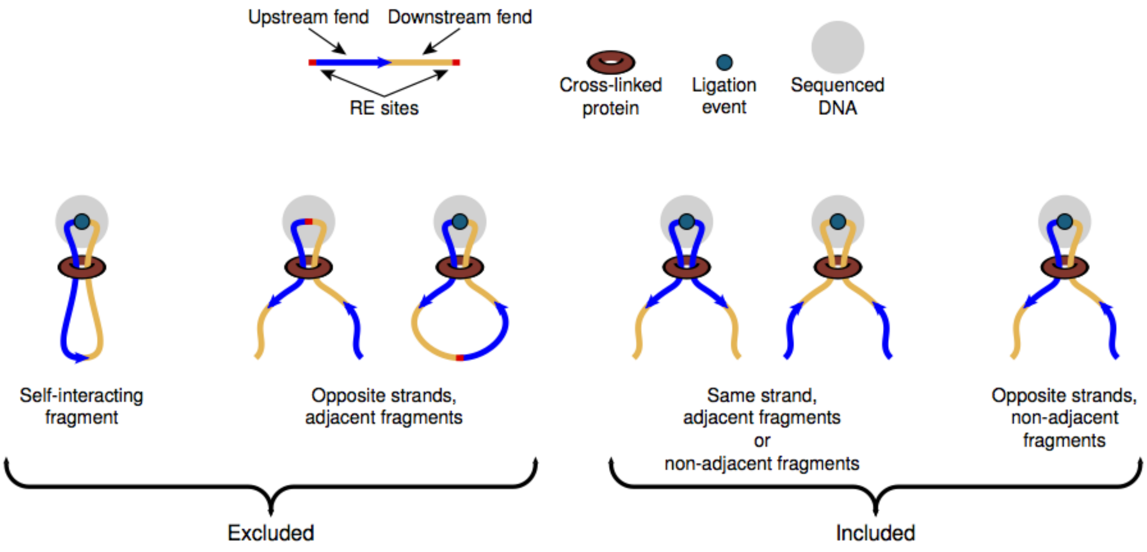

2. HiFive calls a function (*filter_fragments*) after a project is created and loaded with data that iteratively removes reads to produce a set of fends that meet a minimum threshold of interactions with other valid fends on the same chromosome. This filtering is done using the number of interacting fends rather than reads to account for the fact that fend biases can produce wildly different numbers of interactions between fend pairs. This filtering can also be limited to interactions falling within some range limitation of interaction distance. This can be used to ensure that fends have sufficient reads for learning correction parameters when distance range constraints are used for normalization.

CHAPTER 6

Distance-Dependence Estimation

Restriction sites throughout the genome are unevenly distributed. This results in greatly varying sets of distances between fragments and their neighbors. Because interaction signal is strongly inversely-related to inter-fragment distance, this unequal distribution means that fragments with lots of shorter adjacent fragments have a nearby neighborhood of higher interaction values than fragments surrounded by longer restriction fragments simply due to cutsite variation. In order to avoid biases that may result due to restriction site distribution characteristics or the influence of the distance/signal relationship, as well as for estimating enrichment signal, HiFive estimates the distance-dependence relationship from the data prior to normalization using the functions *find_distance_parameters* and *find_distance_parameters* for 5C and HiC, respectively.

6.1 5C Distance-Dependence Estimation

5C data are typically limited to shorter interaction distance ranges (< 10Mb). In addition, the limited number of interactions targeted in a 5C experiment make it feasible to directly estimate the distance-dependence relationship from the data without binning. The ranges that these data span show a fairly good adherence to an inverse power-law relationship, such that the log-interaction signal is inversely linearly related to the log of the inter-fragment distance. Because log-counts are being used, the distance-dependence relationship is found only for non-zero counts.

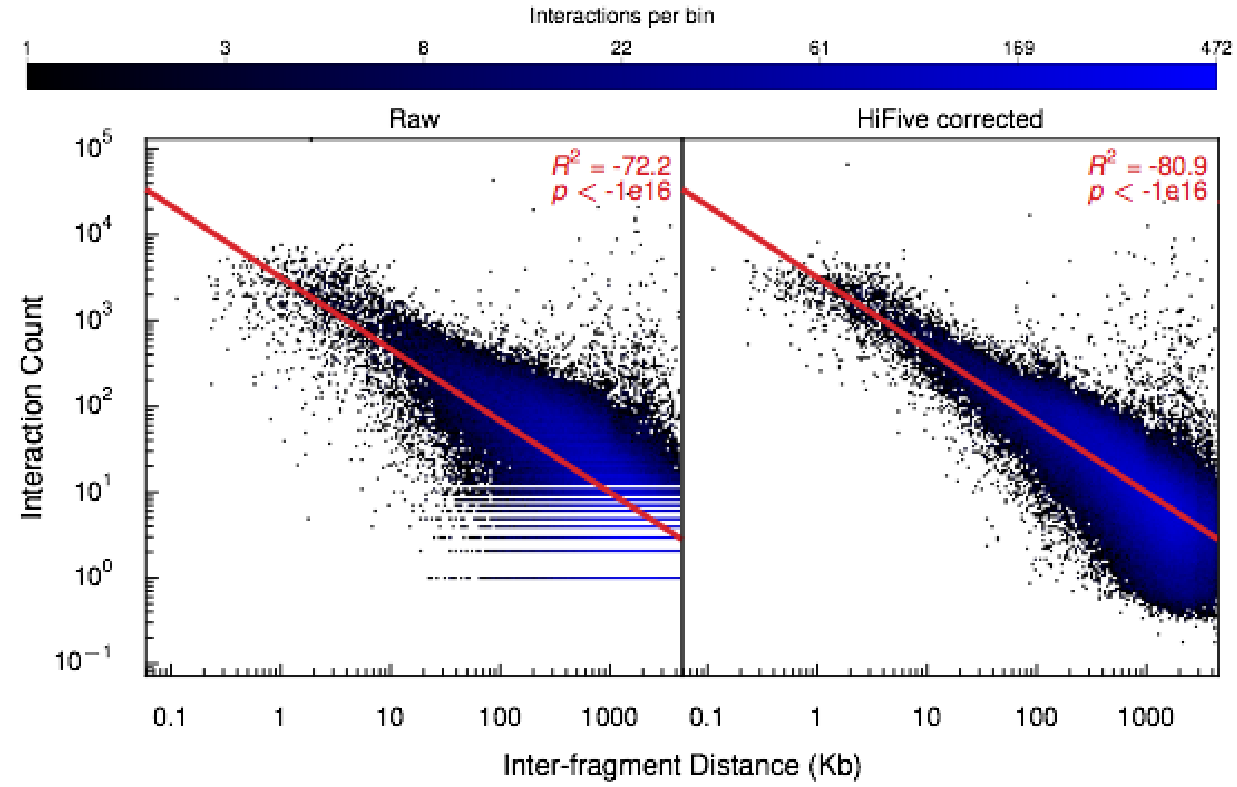

6.2 HiC Distance-Dependence Estimation

HiC data cover a wide range of interaction distances and while portions of the distance curve appear to follow the power-law for some but not all regions. Coupled with the low coverage, especially in long-range cis interactions, HiFive approximates the HiC distance-dependence relationship using a piece-wise linear reconstruction of the relationship between the log-counts and log-distances.

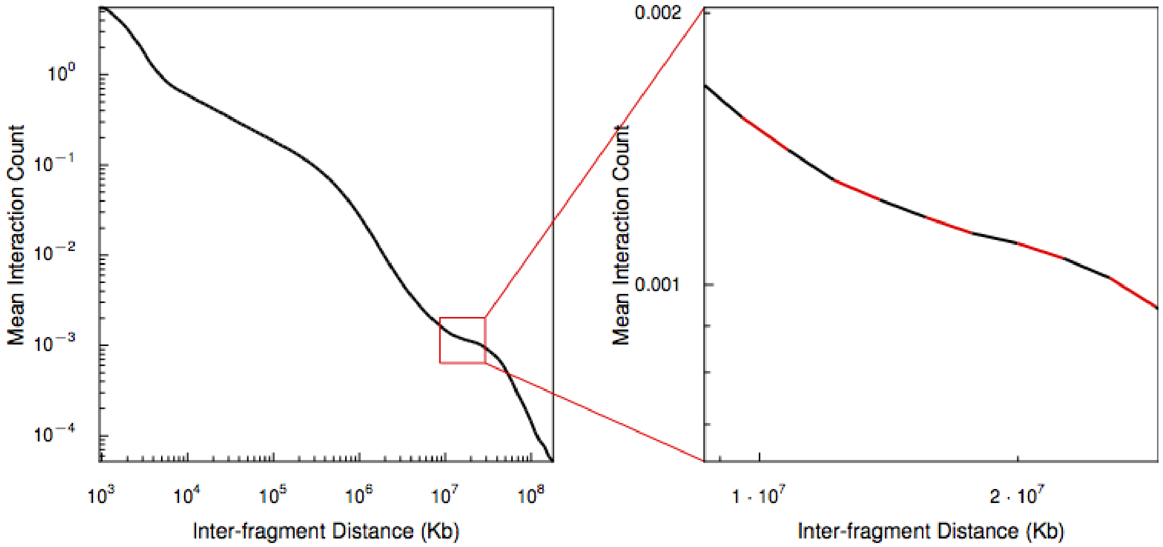

The log-distance range covered by interactions is divided into equal-sized bins and the mean signal and mean log-distance are calculated for each bin. Finally, a series of line segments connecting adjacent log-mean signal and mean

log-distance points are calculated. When estimating the expected distance-dependent signal for a given log-distance, HiFive uses the line segment spanning the query log-distance value. If the value falls before the first mean log-distance or after the last mean log-distance, the nearest line segment equation is used to infer the expected signal.

The same strategy is used to simultaneously estimate a distance-dependence relationship between log-distance and a binary indicator of observed/unobserved instead of counts.

CHAPTER 7

Normalizing Data

HiFive offers several different approaches to normalizing 5C and HiC data. These include three different algorithmic approaches that can be used individually or chained together.

7.1 Probability Algorithm

HiFive's probability-based algorithm models counts as arising from a probability distribution (Binomial or Poisson and Log-Normal for HiC and 5C, respectively) with a mean equal to the product of the contributions from the distance-dependent signal (as determined by the distance-dependence estimation function) and the bias contributions from the fragment or fend at each end of the interaction. In order to learn the fragment or fend bias terms, HiFive pre-calculates the distance-dependence contribution for each cis interaction (intra-chromosomal or intra-regional for HiC and 5C, respectively). Each bias correction can be pre-estimated as the sum of log-counts for a fragment divided by the sum of estimated distance-dependent signal for all interactions involving that fragment for 5C or the sum of interactions (binary, not counts) divided by the sum of the estimated binary distance-dependence signal for all of a fend's interactions.

Parameter optimization is done via a backtracking line gradient descent. This runs until either a maximum number of iterations is reached or all of the gradient absolute values fall below a cutoff threshold.

This algorithm can be limited to learning with a subset of data based on upper and lower distance cutoffs. This is useful for avoiding highly significant structures such as those occurring at short ranges. In the case of HiC data, this also allows a sub-sampling of data to fit within memory limitations for higher-resolution data. This is important as the HiC probability algorithm uses all interactions (both observed and unobserved) to learn correction parameters meaning that memory requirements expand very quickly.

7.2 Express Algorithm

HiFive's Express algorithm is actually two different but very similar algorithms. The first is a modified version of matrix balancing that weights correction updates by the number of valid interactions the associated fragment or fend is involved in (this can be different between fragments, especially when using distance range cutoffs). The other express algorithm is true matrix balancing and uses the Knight and Ruiz algorithm for learning corrections. Both versions allow the use of the matrix-balancing approach with a sparse matrix, making it memory efficient and fast. In addition, both allow correcting for distance dependence prior to learning for better results. In HiC data, values can be considered as true counts or as binary indicators of observed/unobserved. For 5C data, the data are far less sparse so the binary approach does not yield suitable results. Instead, counts may be learned either as is or as log-counts. In addition, for the Knight and Ruiz algorithm it is necessary to incorporate psuedo-counts along the diagonal in order achieve convergence.

7.3 Binning Algorithm

HiFive's binning algorithm is based directly on the multivariate binning modeling approach used by HiCPipe. A set of fragment characteristics (GC content, length, mappability - HiC only, and distance - HiC only) are broken into bins correction values for all bin combinations for each parameter are learned using the Broyden–Fletcher–Goldfarb–Shanno algorithm from the Scipy package. In HiC, distance can be used as an additional parameter, although this is broken into a one-dimensional set of bins. Distance bin parameters are used only during the algorithm learning stage to remove bias and are not kept for later normalization, which is instead taken care of by the distance-dependence estimation function.

For HiC data, this approach uses a binary readout of the data (observed / unobserved) rather than counts and observation probability is assumed to be binomially distributed. Learning can be accomplished using cis, trans, or all interactions. In the case of cis interactions, lower and upper interaction distance cutoffs can be used to limit the set of interactions included in the learning process. If both cis and trans data are used and distance bins are part of the model, the last distance bin is reserved for trans interactions and cis interactions are assigned to the n - 1 remaining bins. You also have the option to add pseudo-counts to the bins. This is done for each bin combination, meaning that for each model parameter, bin1 x bin2 will have twice as many counts added as bin1 x bin1 (to account for both possible combinations). This should be used with caution, as pseudo-counts tend to negatively impact the quality of normalization for cis interactions and HiC datasets typically have enough observations to alleviate the need for pseudo-counts.

For 5C data, only nonzero counts are used and are assumed to be log-normally distributed. For each read, the prior is taken as the predicted distance-dependence signal. Learning can be accomplished using cis, trans, or all interactions. In the case of cis interactions, lower and upper interaction distance cutoffs can be used to limit the set of interactions included in the learning process.

7.4 Chaining Normalization Approaches

Because the corrections learned by the binning algorithm are fundamentally different from those found by the probability and express approaches, they can be combined to account for different aspects of systematic bias within the data. This is accomplished using a two step learning process. Initially the binning algorithm is performed, followed by either the probability or express algorithm using predicted values adjusted based on the binning corrections. This allows both approaches (combinatorial and multiplicative) to be used to best advantage.

CHAPTER 8

Binning Strategies

HiFive uses several different binning formats, depending on the nature of the data being handled. This is both for ease of manipulation and for size issues.

8.1 The 'upper' format

The 'upper' format is used when the data can be represented in a symmetric matrix (bin i,j is equal to j,i). In these cases, HiFive flattens the matrix into a 1D array, using only the upper triangle of the matrix, not including the diagonal. This results in an N * (N - 1) / 2 sized matrix from an N x N square matrix. This is the format used for all cis genomic regions that are binned and have no distance bound on the length of included interactions.

8.2 The 'full' format

The 'full' format is a 2D array that can represent either symmetric or non-symmetric data. This is used to represent trans interaction data and can be requested as the return format of cis interactions. In the case of unbinned 5C data, the 'full' format contains one row and column for every fragment. However, because only forward-reverse combinations are valid the resulting matrix is fairly sparse, akin to a checkerboard pattern.

8.3 The HiC 'compact' format

Because HiC data is often worked with over short ranges, HiFive has a 'compact' format that has an upper bound on the length of interactions included in the array. The array size is equal to the number of bins (or fends in unbinned data) by the maximum number of bins apart that interactions are included for minus one. This means that if we have ten 10 Kb bins and include interactions up to 30 Kb apart, we would need a 10 x 3 array. Position i,j would give us interactions from bin i interacting with bin i + j + 1. This format allows a more efficient way of holding data up to the point that the maximum interaction distance is half of the span of the region or greater, in which case it becomes more efficient to use the 'upper' format. This is only used internally by HiFive or through the API.

8.4 The 5C 'compact' format

Because of the orientation associated with 5C fragment probes, HiFive uses a 'compact' format for unbinned 5C data that has forward fragments along the first axis and reverse fragments along the second axis of the data array. For trans interactions, a pair of regions is represented by two 'compact' arrays, one for each region's set of forward primers by the other region's reverse primers.

8.5 Dynamic Binning

HiFive offers a unique feature to address the variety in read density across 5C and HiC experiments in the form of dynamic binning. Rather than selecting an arbitrary bin size and contending with bins that don't contain enough or any observations, HiFive has an option for expanding each bin to meet a user-defined threshold of observations. This expansion can draw from unbinned data or binned data and allows each bin in a heatmap to contain sufficient information to be reasonably reliable. For each bin, the bounds expand in all directions equally, incorporating new observations based on expansion bin or fragment midpoints. After each incorporation, the observation threshold is checked and if satisfied, the expansion is ceased. A maximum search distance is also used to prevent excessive running time. If binned data is used for the expansion, the bin will encounter new observations in all directions simultaneously. If unbinned data are used, each expansion will include the nearest fragment or fend and its associated observed and expected values.

Multi-Resolution Heatmap

HiFive has a unique filetype called a 'multi-resolution heatmap' or MRH, which is a binary file holding information necessary for plotting any part of the genome across a wide range of resolutions using intelligent binning based on observation density. Files are indexed in a recursive fashion making retrieval incredibly fast. This makes exploring HiC data incredibly easy, either through the included plotting script or when combined with the interactive HiC browser for multi-resolution heatmaps support in Galaxy.

9.1 MRH Approach

The idea behind the multi-resolution heatmap is to have a file that is as small as possible while containing data in the highest resolution reasonable given the density of information present. Each chromosome (or chromosome pair for inter-chromosomal interactions) is binned at a low resolution. Each bin is designated as valid or invalid based on an observation cutoff (the number of reads that must be seen in a given bin). Each valid bin is then subdivided into four equal-sized bins. This respresents the next level of resolution. These new bins are also subjected to the same read cutoff. Lower resolution bins also have associated with them an index specifying where their subdivided bins are located in the file and a shape indicator specifying how many and which sub-bins are valid. This has two advantages. First, there are no invalid bins included in the file past the lowest level of resolution, and second, retrieving data from any pair of coordinate ranges is easy. This is because to locate any 2D position in the heatmap, one simple identifies the correct low-resolution bin and then recursively selects the appropriate subdivision. The final advantage is that for positions that have insufficient data at a higher level of resolution, the value of a lower resolution bin containing the target position can be returned. This makes visual exploration much easier as there are effectively no missing values.

9.2 Standalone MRH program

HiFive includes a command line program, 'fetch_mrh_data', that allows data to be retrieved from an MRH file and either plotted as an image or written to a genomic interval text file.

```
> fetch_mrh_data [-h] -c CHROM [-C CHROM2] [-s START] [-S START2] [-e END]
                 [-E END2] [-R MAXRES] [-r MINRES] [-t] heatmap output
```

Arguments:

project The HiFive MRH file to create pull data from.

output A filename to write the image or interval file to.

Options:

-h, --help Display the help message, options, and arguments and exit.

-c, --chrom str The first region chromosome to pull data from.

-C, --chrom2 str The second region chromosome to pull data from. If no value is passed, this will be set to the same value as 'chrom'.

-s, --start int The first coordinate of the chromosome to pull data from. If no value is passed, this will be set to the first bin position in the MRH file for 'chrom'. [None]

-S, --start2 int The first coordinate of the chromosome to pull data from. If no value is passed, this will be set to the first bin position in the MRH file for 'chrom2'. [None]

-e, --end int The last coordinate + 1 of the chromosome to pull data from. If no value is passed, this will be set to the last bin position in the MRH file for 'chrom'. [None]

-E, --end2 int The last coordinate + 1 of the chromosome to pull data from. If no value is passed, this will be set to the last bin position in the MRH file for 'chrom2'. [None]

-R, --max-resolution The maximum resolution bound for returned data. If no value is passed, this will be set to the highest resolution available in the heatmap for the chromosome(s). [default: None]

-r, --min-resolution The minimum resolution bound for returned data. If no value is passed, this will be set to the lowest resolution available in the heatmap for the chromosome(s). [default: None]

-t, --text Write a genomic interval text file instead of an image.

9.3 File specs

The MRH file is composed of a header and series of inline 1D arrays of data. The header contains information about what chromosomes are present, if inter-chromosomal interactions are present, and coordinate and score information for each chromosome/chromosome-pair. For each chromosome and chromosome-pair, there are three arrays of information. the First contains the enrichment data, the second contains bin index values, and the third contains information about the validity of bins for each resolution level. All values in the file are big-endian.

9.3.1 The File Header

The header contains the following information:

magic number A filetype identifier of 4 bytes, '0x42054205'

interchromosomal flag A 32-bit integer of 1 or 0, indicating if inter-chromosomal interactions are included or not.

number of chromosomes A 32-bit integer specifying how many chromosomes are in the files index.

chromosome name sizes An N * 32-bit integer array indicating the length in characters of each of N chromosome names.

chromosome names A sum(name sizes) * 1-byte character array containing each chromosome's name.

chromosome bit index An index of the starting bit for each chromosome/chromosome-pair. If inter-chromosomal data is not included, this is an N * 32-bit integer array. Otherwise this is a N * (N - 1) / 2 * 32-bit integer array with chromosomes ordered 1x1, 1x2... 1xN, 2x2, 2x3... NxN. All subsequent arrays for chromosomes/chromosome-pairs are ordered the same way.

chromosome partitions An N * 32-bit integer array specifying the number of genomic paritions (bins along 1 axis) each chromosome is partitioned into.

trans chromosome partitions If trans interactions are included, this is an N * 32-bit integer array specifying the number of genomic paritions (bins along 1 axis) each chromosome is partitioned into for inter-chromosomal interactions.

data array sizes A # chroms/pairs * 32-bit integer array specifying how many 32-bit floating point values each chromosome/pair's data array contains.

index array sizes A # chroms/pairs * 32-bit integer array specifying how many 32-bit integer values each chromosome/pair's index array contains.

chromosome starting coordinates An N * 32-bit integer array specifying the first coordinate of each chromosome's smallest intra-chromosomal bin.

trans chromosome starting coordinates If trans interactions are included, an N * 32-bit integer array specifying the first coordinate of each chromosome's smallest inter-chromosomal bin.

chromosome stopping coordinates An N * 32-bit integer array specifying the last coordinate of each chromosome's largest intra-chromosomal bin.

trans chromosome stopping coordinates If trans interactions are included, an N * 32-bit integer array specifying the last coordinate of each chromosome's largest inter-chromosomal bin.

smallest enrichment scores A # chroms/pairs * 32-bit floating point array of values with the smallest enrichment value for each chromosome/pair.

largest enrichment scores A # chroms/pairs * 32-bit floating point array of values with the largest enrichment value for each chromosome/pair.

maximum bin size A 32-bit integer specifying the largest bin size (lowest resolution) for intra-chromosomal interactions.

maximum trans bin size If trans interactions are included, a 32-bit integer specifying the largest bin size (lowest resolution) for inter-chromosomal interactions.

minimum bin size A 32-bit integer specifying the smallest bin size (highest resolution) for intra-chromosomal interactions.

minimum trans bin size If trans interactions are included, a 32-bit integer specifying the smallest bin size (highest resolution) for inter-chromosomal interactions.

minimum observation cutoff A 32-bit integer specifying the minimum number of observations needed for a bin to be included in the MRH file.

9.3.2 Data Arrays

Data for each chromosome and chromosome-pair consists of three arrays, the interaction, index, and shape arrays. The first contains all of the actual enrichment values. The interaction array contains data as 32-bit floating point values grouped by resolution level, going from lowest (largest bin size) to highest. The lowest resolution data contains all possible bins within the coordinate bounds, either a flattened rectangular array (row-major) for inter-chromosomal data or a flattened upper-triangle array for intra-chromosomal data. Subsequent resolution data is presented in groups of 4 or fewer bins.

For each interaction bin, excepting the lowest resolution bins, there is a corresponding index bin and shape bin in the same order. The index bin specifies the index in the data array containing the next level of resolution up for data within the current bin. If no valid higher resolution bins are contained within the current bin's boundaries, then the index bin has a value of -1. The shape bin contains a 16-bit integer that uses the first 4 bits to indicate which of the four possible bins contain valid data in a row-major order. For example a value of 9 would indicate the top left and bottom right corners of the 2 by 2 grid would have valid values. This is also used to indicate how many bins at that higgher resolution correspond to the current bin.

CHAPTER 10

Using HiFive

HiFive contains a variety of subcommands for handling different aspects of 5C and HiC analysis. To learn about a specific subcommand, you can use the following command:

```
> hifive <subcommand> -h
```

The available subcommands are:

fragments Create a fragment file from a BED file containing targeted RE fragment data.

5c-data Create a data file from mapped BAM or fragment-pair counts files.

5c-project Create a project file, filter fragments, and estimate distance-dependence.

5c-normalize Find correction parameter values using one of the available algorithms.

5c-complete Perform all of the steps of the subcommands fragments, 5c-data, 5c-project, and 5c-normalization in one command.

5c-heatmap Using an already created 5C project, generate an HDF5-formatted heatmap file and optional image.

5c-interval Using an already created 5C project, generate a tabular genomic-interval file for a specified region and optional image.

5c-combine-replicates Combine multiple 5C data files into a single file without needing to reload the data.

fends Create a fend file from either a BED or HiCPipe-style fend file containing RE fragment data.

hic-data Create a data file from mapped BAM, MAT, or paired coordinate text files.

hic-project Create a project file, filter fends, and estimate distance-dependence.

hic-normalize Find correction parameter values using one of the available algorithms.

hic-complete Perform all of the steps of the subcommands fends, hic-data, hic-project, and hic-normalization in one command.

hic-heatmap Using an already created HiC project, generate an HDF5-formatted heatmap file and optional image.

hic-interval Using an already created HiC project, generate a tabular genomic-interval file for a specified region and optional image.

hic-combine-replicates Combine multiple HiC data files into a single file without needing to reload the data.

hic-mrheatmap Create a multi-resolution heatmap file from a HiFive HiC project file.

10.1 5C Subcommands

Each subcommand uses its own set of options although there is some overlap of options between subcommands.

10.1.1 fragments

```
> hifive fragments [-h] [-r RE] [-g GENOME] [-q] bed output
```

Arguments:

bed A BED file with targeted restriction enzyme fragment coordinates and the associated name of the primer used to target each fragment. Additional fields can be included with RE fragment characterstics (such as GC content) in columns after the strand column. Including additional features requires a header with the name of each feature.

output A filename to write the HiFive Fragment file to.

Options:

-h/–help, -r/–re, -g/–genome, -q/–quiet

10.1.2 5c-data

```
> hifive 5c-data [-h] (-B BAM BAM | -C COUNT) [-q] fragment output
```

Arguments:

fragment The HiFive Fragment file to associate with this dataset.

output A filename to write the HiFive 5C dataset to.

Options:

-h/–help, -B/–bam, -C/–count, -q/–quiet

10.1.3 5c-project

```
> hifive 5c-project [-h] [-f MININT] [-m MINDIST] [-x MAXDIST] [-q]
      data output
```

Arguments:

data The HiFive 5C dataset file to associate with this project.

output A filename to write the HiFive 5C project to.

Options:

-h/–help, -f/–min-interactions, -m/–min-distance, -x/–max-distance, -q/–quiet

10.1.4 5c-normalize

```
> hifive 5c-normalize <SUBCOMMAND> [-h] [-m MINDIST] [-x MAXDIST]
      [-r REGIONS] [-o OUTPUT] [-q] [normalization options] project
```

HiFive's 5C normalization subcommand allows the user to select the normalization approach to use. Each approach has its own set of options.

Arguments:

project The HiFive 5C project to find fragment corrections for.

Options:

-h/–help, -m/–min-distance, -x/–max-distance, -r/–regions, -o/–output, -q/–quiet

Subcommands:

probability, express, binning, probability-binning, express-binning, binning-probability, binning-express

10.1.5 5c-complete

```
> hifive 5c-complete <SUBCOMMAND> [-h] [-r RE] [-g GENOME]
      (-B BAM BAM | -C COUNT) [-f MININT] [-m MINDIST] [-x MAXDIST]
      [-r REGIONS] (-o OUTPUT OUTPUT OUTPUT | -P PREFIX) [-q]
      [normalization options] bed
```

HiFive's complete 5C analysis subcommand allows the user to select the normalization approach to use. Each approach has its own set of options.

Arguments:

bed A BED file with targeted restriction enzyme fragment coordinates and the associated name of the primer used to target each fragment.

Options:

-h/–help, -r/–re, -g/–genome, -B/–bam, -C/–count, -f/–min-interactions, -m/–min-distance, -x/–max-distance, -r/–regions, -o/–output, -P/–prefix -q/–quiet

Subcommands:

probability, express, binning, probability-binning, express-binning, binning-probability, binning-express

10.1.6 5c-heatmap

```
> hifive 5c-heatmap [-h] [-b BINSIZE] [-t] [-r REGIONS]
      [-d {raw,fragment,distance,enrichment,expected}]
      [-a {compact,full}] [-y] [-x EXPBINSIZE] [-f MINOBS]
      [-g SEARCH] [-v] [-i IMAGE] [-p] [-l] [-n]
      [-k KEYWORDS] [-q] project output
```

Arguments:

project The HiFive 5C project to create a heatmap for.

output The filename to write the HiFive 5C heatmap to.

Options:

-h/–help, -b/–binsize, -t/–trans, -r/–regions, -d/–datatype, -a/arraytype, -y/–dynamically-bin, -x/–expansion-binsize, -f/–minobservations, -g/–search-distance, -v/–remove-failed, -i/–image, -p/–pdf, -l/–legend, -n/–names, -k/–keyword, -q/–quiet

10.1.7 5c-interval

```
> hifive 5c-interval [-h] -c REGION [-s START] [-e STOP] [--region2 REGION2]
      [--start2 START2] [--stop2 STOP2] [-b BINSIZE]
      [-d {raw,fragment,distance,enrichment,expected}] [-y] [-x EXPBINSIZE]
      [-f MINOBS] [-g SEARCH] [-v] [-i IMAGE] [-p] [-r] [-t] [-l]
      [-k KEYWORDS] [-q] project output
```

Arguments:

project The HiFive 5C project to create a heatmap for

output The filename to write the HiFive 5C genomic interval file to.

Options:

-h/–help, -c/–region, -s/–start, -e/–stop, -b/–binsize, -d/–datatype, -y/–dynamically-bin, -x/–expansion-binsize, -f/–minobservations, -g/–search-distance, -v/–remove-failed, -i/–image, -p/–pdf, -r/–rotate, -t/–ticks, -l/–legend, -k/–keyword, -q/–quiet

10.1.8 5c-combine-replicates

```
> hifive 5c-combine-replicates [-h] [-q] output replicate
      [replicate ...]
```

Arguments:

output The filename to write the new HiFive 5C dataset file to.

replicate A HiFive 5C dataset file.

Options:

-h/–help, -q/–quiet

10.2 5C Options

Universal Options:

-h, --help Display the help message and command/subcommand options and arguments and exit.

-q, --quiet Suppress all messages generated during HiFive processing.

5C Fend Options:

-r, --re str The name of the restriction enzyme.

-g, --genome str The name of the genome.

5C Data Options:

-B, --bam FILES A pair of BAM filenames separated by spaces corresponding to the two independently-mapped ends of a set of reads. Multiple file pairs may be passed by calling this argument more than once. This option is mutually exclusive with -C/–count.

-C, --count FILE A tabular text file containing pairs of fragment primer names and their associated read count (see Loading 5C Data for more information). This option is mutually exclusive with -B/–bam.

5C Project Options:

-f, --min-interactions int The minimum number of interactions with valid fragments to keep a fragment in the analysis. [20]

-m, --min-distance int The minimum distance between fragment midpoints to include in calculating numbers of interactions for fragment filtering and (if called by 5c-normalization or 5c-complete) the minimum interaction distance included in learning correction parameter values. [0]

-x, --max-distance int The maximum distance between fragment midpoints to include in calculating numbers of interactions for fragment filtering and (if called by 5c-normalization or 5c-complete) the maximum interaction distance included in learning correction parameter values. A value of zero indicates no maximum distance cutoff. [0]

5C Normalization Options:

-r, --regions str A comma-separated list of region numbers to include fragments from when calculating correction parameter values. [all regions]

-o, --output FILE An optional filename to save the updated HiFive project to, leaving the original unchanged. [None]

5C Complete Options:

-o, --output FILES A set of three filenames separated by spaces to save the newly-created HiFive fragment, dataset, and project files to. Mutually exclusive with -P/–prefix.

-P, --prefix str A prefix for the output filenames. The file extensions .frags, .fcd, and .fcp will be used for the fragment, dataset, and project files, respectively. This option is mutually exclusive with -o/–output.

10.2.1 5C Normalization Algorithms

5C Probability Options:

-b, --max-iterations int The maximum number of iterations to run the learning process for. [1000]

-g, --min-change dec The minimum allowable absolute gradient size to coninute learning process. [0.0005]

-p, --precalculate Prior to beginning learning, set initial guesses for each correction value to be learned to the fragment's mean difference between its log-counts and predicted distance-dependence signal.

-l, --learning-step dec The scaling factor for decreasing learning rate by if step doesn't meet Armijo criterion. [0.5]

5C Express Options:

-e, --express-iterations int The number of iterations to run the learning process for. [1000]

-d, --remove-distance Calculate and subtract out the predicted distance-dependence signal from each log-count prior to learning correction parameters.

-w, --express-reads str Which set reads to use for learning correction parameter values, cis, trans, or all. [cis]

-k, --logged Use log-counts instead of counts for learning.

-z, --knight-ruiz Use the Knight Ruiz matrix balancing algorithm instead of weighted matrix balancing. This option ignores 'iterations' and 'logged'.

5C Binning Options:

-i, --binning-iterations int The maximum number of iterations to run the learning process for. [1000]

-t, --learning-threshold dec The maximum change in log-likelihood necessary to stop the learning process early. [1.0]

-y, --binning-reads str Which set of reads to use for learning correction parameter values, cis, trans, or all. [cis]

-v, --model str A comma-separated list of fragment features to calculate corrections for. Acceptable features are len (length) and any features loaded in the BED file used to create the HiFive fragment file. [len]

-n, --model-bins str A comma-separated list of numbers of bins to partition fragment features into for modeling. [10]

-u, --parameter-types str A comma-separated list of model parameter types. Acceptable values are even, fixed, even-const, and fixed-const. Even means that fragment features are partitioned such that each bin has approximately even numbers of fragments. Fixed means that the range of the feature is divided into fixed-width bins. The -const suffix indicates that the correction values are held at their seed-values and not updated. [even]

10.2.2 5C Interaction Binning Options

5C Heatmap Options:

-b, --binsize int The width of bins (in basepairs) to partition data into. A value of zero indicates that each bin is to correspond with a single fragment. [10000]

-t, --trans Calculate and include trans interactions in heatmaps.

-r, --regions str A comma-separated list if region numbers to include in the heatmaps. [all regions]

-d, --datatype str Type of data to produce for the heatmaps. Valid options are raw, fragment (only fragment corrections applied), distance (only distance-dependence signal removed), enrichment (both fragment correction and distance-dependence signal removed), and expected (only predicted signal). [fragment]

-a, --arraytype str If data is unbinned, this option specifies whether the heatmaps should be full or compact. Full means that there is a row and column for every fragment, while compact means that rows are forward fragments only and columns are reverse fragments only. [full]

-y, --dynamically-bin Dynamically bin heatmap.

-x, --expansion-binsize int The size of bins, in base pairs, to group data into for expanding under-populated bins. [10000]

-f, --minobservations int The minimum number of observed reads in a bin for it to be considered valid. [20]

-g, --search-distance int The furthest distance from the bin minpoint to expand bounds. If set to zero, there is no limit on expansion distance. [0]

-v, --remove-failed If a non-zero 'search-distance' is given, it is possible for a bin not to meet the 'minobservations' criteria before stopping looking. If this occurs and 'remove-failed' is set, the observed and expected values for that bin are zero.

5C Interval Options:

-c, --region int The index of the region to pull data from.

-b, --binsize int The width of bins (in basepairs) to partition data into. A value of zero indicates that each bin is to correspond with a single fragment.

-s, --start int The first coordinate of the region to pull data from. None indicates the beginning of the region. [None]

-e, --stop int The last coordinate + 1 of the region to pull data from. None indicates the end of the region. [None]

-y, --dynamically-bin Dynamically bin heatmap.

-x, --expansion-binsize int The size of bins, in base pairs, to group data into for expanding under-populated bins. [10000]

-f, --minobservations int The minimum number of observed reads in a bin for it to be considered valid. [20]

-g, --search-distance int The furthest distance from the bin minpoint to expand bounds. If set to zero, there is no limit on expansion distance. [0]

-v, --remove-failed If a non-zero ‘search-distance’ is given, it is possible for a bin not to meet the ‘minobservations’ criteria before stopping looking. If this occurs and ‘remove-failed’ is set, the observed and expected values for that bin are zero.

5C Plotting Options:

-i, --image FILE Generate an image from the region or regions for which heatmap data is being calculated. [None]

-p, --pdf Format the image as a pdf. [None]

-r, --rotate Rotate the image 45 degrees so the chromosome axis is horizontal and only plot the triangle above this axis. This option can only be used with a full arraytype.

-t, --ticks Add coordinate ticks and labels to heatmap. This option can only be used if a pdf is requested.

-l, --legend Add a color scale bar corresponding to interaction strength. This option can only be used if a pdf is requested.

-n, --names Add region names to the plot. This option can only be used if a pdf is requested.

-k, --keyword str Pass additional plotting options accepted by the `plotting` module. Arguments should be of the format KEYWORD=VALUE. This option can be passed multiple times. [None]

10.3 HiC Subcommands

Each subcommand uses its own set of options although there is some overlap of options between subcommands.

10.3.1 fends

```
> hifive fends [-h] (-F FEND | -B BED) [-r RE] [-g GENOME] [-q] output
```

Arguments:

output A filename to write the HiFive Fend file to.

Options:

-h/–help, -F/–fend, -B/–bed, -r/–re, -g/–genome, -q/–quiet

10.3.2 hic-data

```
> hifive hic-data [-h] (-S BAM BAM | -R RAW | -M MAT) [-i INSERT] [-q]
      fend output
```

Arguments:

fragment The HiFive Fend file to associate with this dataset.

output A filename to write the HiFive HiC dataset to.

Options:

-h/–help, -S/–bam, -R/–raw, -M/–mat, -i/–insert, -q/–quiet

10.3.3 hic-project

This command is MPI-compatible.

```
> [mpirun -np NP] hifive hic-project [-h] [-f MININT] [-m MINDIST]
                      [-x MAXDIST] [-j MINBIN] [-n NUMBINS] [-q] data
                      output
```

Arguments:

data The HiFive HiC dataset file to associate with this project.

output A filename to write the HiFive HiC project to.

Options:

-h/–help, -f/–min-interactions, -m/–min-distance, -x/–max-distance, -j/–min-binsize, -n/–num-bins, -q/–quiet

10.3.4 hic-normalize

This command is MPI-compatible.

```
> [mpirun -np NP] hifive hic-normalize <SUBCOMMAND> [-h] [-m MINDIST]
                      [-x MAXDIST] [-c CHROMS] [-o OUTPUT] [-q]
                      [normalization options] project
```

HiFive's HiC normalization subcommand allows the user to select the normalization approach to use. Each approach has its own set of options.

Arguments:

project The HiFive HiC project to find fragment corrections for.

Options:

-h/–help, -m/–min-distance, -x/–max-distance, -c/–chromosomes, -o/–output, -q/–quiet

Subcommands:

probability, express, binning, binning-probability, binning-express

10.3.5 hic-complete

This command is MPI-compatible.

```
> [mpirun -np NP] hifive hic-complete <SUBCOMMAND> [-h]
                      (-F FEND | -B BED) [-r RE] [-g GENOME]
                      (-S BAM BAM | -R RAW | -M MAT) [-i INSERT]
                      [-f MININT] [-m MINDIST] [-x MAXDIST]
                      [-j MINBIN] [-n NUMBINS] [-c CHROMS]
                      (-o OUTPUT OUTPUT OUTPUT | -P PREFIX) [-q]
                      [normalization options]
```

HiFive's complete HiC analysis subcommand allows the user to select the normalization approach to use. Each approach has its own set of options.

Options:

-h/–help, -F/–fend, -B/–bed, -r/–re, -g/–genome, -S/–bam, -R/–RAW, -M/–mat, -f/–min-interactions, -m/–min-distance, -x/–max-distance, -j/–min-binsize, -n/–num-bins, -c/–chromosomes, -o/–output, -P/–prefix -q/–quiet

Subcommands:

probability, express, binning, binning-probability, binning-express

10.3.6 hic-heatmap

This command is MPI-compatible.

```
> [mpirun -np NP] hifive hic-heatmap [-h] [-b BINSIZE] [-t]
                       [-c CHROMS]
                       [-d {raw,fend,distance,enrichment,expected}]
                       [-y] [-x EXPBINSIZE] [-f MINOBS] [-a SEARCH]
                       [-v]  [-i IMAGE] [-p] [-l] [-n] [-k KEYWORDS]
                       [-q] project output
```

Arguments:

project The HiFive HiC project to create a heatmap for.

output The filename to write the HiFive HiC heatmap to.

Options:

-h/–help, -b/–binsize, -t/–trans, -c/–chromosomes, -d/–datatype, -y/–dynamically-bin, -x/–expansion-binsize, -f/–minobservations, -a/–search-distance, -v/–remove-failed, -i/–image, -p/–pdf, -l/–legend, -n/–names, -k/–keyword, -q/–quiet

10.3.7 hic-interval

```
> hifive hic-interval [-h] -c CHROM [-s START] [-e STOP] [-b BINSIZE]
      [-m MAXDIST] [-d {raw,fend,distance,enrichment,expected}] [-y]
      [-x EXPBINSIZE] [-f MINOBS] [-a SEARCH] [-v] [-i IMAGE] [-p]
      [-r] [-t] [-l] [-k KEYWORDS] [-q] project output
```

Arguments:

project The HiFive HiC project to create a heatmap for.

output The filename to write the HiFive HiC genomic interval file to.

Options:

-h/–help, -c/–chromosome, -s/–start, -e/–stop, -b/–binsize, -m/–max-distance, -d/–datatype, -y/–dynamically-bin, -x/–expansion-binsize, -f/–minobservations, -a/–search-distance, -v/–remove-failed, -i/–image, -p/–pdf, -r/–rotate, -t/–ticks, -l/–legend, -k/–keyword, -q/–quiet

10.3.8 hic-combine-replicates

```
> hifive hic-combine-replicates [-h] [-q] replicate1 replicate2 output
```

Arguments:

replicate1 The first HiFive HiC dataset file to be combined.

replicate2 The second HiFive HiC dataset file to be combined.

output The filename to write the new HiFive HiC dataset file to.

Options:

-h/–help, -q/–quiet

10.3.9 hic-mrheatmap

```
> hifive hic-mrheatmap [-h] [-t] [-c CHROMS] [-f MINOBS] [-B MAXBIN]
      [-b MINBIN] [-R MAXTRANSBIN] [-r MINTRANSBIN] [-m MIDBIN]
      [-d {raw,fend,distance,enrichment}] [-q] project output
```

Arguments:

project The HiFive HiC project to create a multi-resolution heatmap for.

output The filename to write the multi-resolution heatmap to.

Options:

-h/–help, -q/–qiuet, -t/–trans, -c/–chromosomes, -f/–minobservations, -B/–maximum-binsize, -b/–minimum-binsize, -R/–maximum-trans-binsize, -r/–minimum-trans-binsize, -m/–mid-binsize, -d/–datatype,

10.4 HiC Options

Universal Options:

-h, --help Display the help message and command/subcommand options and arguments and exit.

-q, --quiet Suppress all messages generated during HiFive processing.

HiC Fend Options:

-F, --fend FILE A tabular file in a format compatible with HiCPipe containing fragment and fend indices, fragment length, start or end position, and any additional fragment features desired (see Loading HiC Fends for more information).

-B, --bed FILE A BED file containing either restriction enzyme fragment coordinates or retriction enzyme cutsite coordinates. Fragment features may be included in columns after the strand column. Features should be formatted with one feature per column and two values per feature separated by a comma. If the coordinates are of

RE fragment boundaries, the feature values should correspond to the upstream end of the fragment followed by the downstream end of the fragment. If the coordinates are of RE cutsites, the values should correspond to the sequence just upstream of the cutsite followed by the sequence just downstream of the cutsite. If additional features are included, the bed file must have a header line identifying the features.

-r, --re str The name of the restriction enzyme.

-g, --genome str The name of the genome.

HiC Data Options:

-S, --bam FILES A pair of BAM filenames separated by spaces corresponding to the two independently-mapped ends of a set of reads. Multiple file pairs may be passed by calling this argument more than once. This option is mutually exclusive with -R/–raw and -M/–mat.

-R, --raw FILE A tabular file containing pairs of mapped read positions (see Loading HiC Data for more information).

-M, --mat FILE A tabular file containing pairs of fend indices and their corresponding numbers of reads (see Loading HiC Data for more information).

-i, --insert int The maximum allowable insert size, as measured by the sum of both read end mapping positions to the nearest RE cutsite in the direction of alignment.

HiC Project Options:

-f, --min-interactions int The minimum number of interactions with valid fends to keep a fend in the analysis. [20]

-m, --min-distance int The minimum distance between fend midpoints to include in calculating numbers of interactions for fend filtering and (if called by hic-normalization or hic-complete) the minimum interaction distance included in learning correction parameter values. [0]

-x, --max-distance int The maximum distance between fend midpoints to include in calculating numbers of interactions for fend filtering and (if called by hic-normalization or hic-complete) the maximum interaction distance included in learning correction parameter values. A value of zero indicates no maximum distance cutoff. [0]

-j, --min-binsize int The cutoff size limit for the smallest distance bin used for estimating the distance dependence (see HiC Distance Dependence Estimation for more information). [1000]

-n, --num-bins int The number of bins to partition the interaction size ranges into for estimating the distance dependence function (see HiC Distance Dependence Estimation for more information). A value of zero indicates that finding the distance dependence function should be skipped.

HiC Normalization Options:

-c, --chromosomes str A comma-separated list of chromosome names to include fends from when calculating correction parameter values. [all chromosomes]

-o, --output FILE An optional filename to save the updated HiFive project to, leaving the original unchanged. [None]

HiC Complete Options:

-o, --output FILES A set of three filenames separated by spaces to save the newly-created HiFive fend, dataset, and project files to. Mutually exclusive with -P/–prefix.

-P, --prefix str A prefix for the output filenames. The file extensions .fends, .hcd, and .hcp will be used for the fragment, dataset, and project files, respectively. This option is mutually exclusive with -o/–output.

10.4.1 HiC Normalization Algorithms

HiC Probability Options:

-b, --max-iterations int The maximum number of iterations to run the learning process for. [1000]

-g, --min-change dec The minimum allowable absolute gradient size to coninute learning process. [0.0005]

-p, --precalculate Prior to beginning learning, set initial guesses for each correction value to be learned to the fragment's mean difference between its log-counts and predicted distance-dependence signal.

-l, --learning-step dec The scaling factor for decreasing learning rate by if step doesn't meet Armijo criterion. [0.5]

-a, --probability-model Which probability model to use for normalization (binomial or poisson).

HiC Express Options:

-e, --express-iterations int The number of iterations to run the learning process for. [1000]

-d, --remove-distance Calculate and divide out the predicted distance-dependence signal from each count prior to learning correction parameters.

-w, --express-reads str Which set reads to use for learning correction parameter values, cis, trans, or all. [cis]

-g, --min-change The minimum mean change in fend correction parameter values needed to keep running past 'iterations' number of iterations. If using the Knight-Ruiz algorithm this is the residual cutoff.

-f, --min-interations int The minimum number of interactions for fend filtering if refiltering is required due to distance cutoff parameters or selected reads to be used. [20]

-k, --binary bool Use binary indicator instead of counts.

-z, --knight-ruiz bool Use the Knight Ruiz matrix balancing algorithm instead of weighted matrix balancing. This option ignores 'iterations'.

HiC Binning Options:

-r, --binning-iterations int The maximum number of iterations to run the learning process for. [1000]

-t, --learning-threshold dec The maximum change in log-likelihood necessary to stop the learning process early. [1.0]

-y, --binning-reads str Which set of reads to use for learning correction parameter values, cis, trans, or all. [cis]

-v, --model str A comma-separated list of fend features to calculate corrections for. Acceptable features are len (length), distance, and any features loaded in the BED or FEND file used to create the HiFive fend file. [len,distance]

-s, --model-bins str A comma-separated list of numbers of bins to partition fend features into for modeling. [20,20]

-u, --parameter-types str A comma-separated list of model parameter types. Acceptable values are even, fixed, even-const, and fixed-const. Even means that fend features are partitioned such that each bin has approximately even numbers of fends. Fixed means that the range of the feature is divided into fixed-width bins. The -const suffix indicates that the correction values are held at their seed-values and not updated. [even,fixed-const]

--pseudocounts int The number of pseudo-counts to add to each bin prior to seeding and learning normalization values. [None]

10.4.2 HiC Interaction Binning Options

HiC Heatmap Options:

-b, --binsize int The width of bins (in basepairs) to partition data into. A value of zero indicates that each bin is to correspond with a single fend. [10000]

-t, --trans Calculate and include trans interactions in heatmaps.

-c, --chromosomes str A comma-separated list if chromosome names to include in the heatmaps. [all chromosomes]

-d, --datatype str Type of data to produce for the heatmaps. Valid options are raw, fend (only fend corrections applied), distance (only distance-dependence signal removed), enrichment (both fend correction and distance-dependence signal removed), and expected (only predicted signal). [fend]

-y, --dynamically-bin Dynamically bin heatmap.

-x, --expansion-binsize int The size of bins, in base pairs, to group data into for expanding underpopulated bins. [10000]

-f, --minobservations int The minimum number of observed reads in a bin for it to be considered valid. [20]

-a, --search-distance int The furthest distance from the bin minpoint to expand bounds. If set to zero, there is no limit on expansion distance. [0]

-v, --remove-failed If a non-zero 'search-distance' is given, it is possible for a bin not to meet the 'minobservations' criteria before stopping looking. If this occurs and 'remove-failed' is set, the observed and expected values for that bin are zero.

HiC Interval Options:

-c, --chromosome str The chromosome to pull data from.

-b, --binsize int The width of bins (in basepairs) to partition data into. A value of zero indicates that each bin is to correspond with a single fend.

-s, --start int The first coordinate of the chromosome to pull data from. None indicates the beginning of the chromosome. [None]

-e, --stop int The last coordinate + 1 of the chromosome to pull data from. None indicates the end of the chromosome. [None]

-m, --max-distance int The largest interaction distance to include in the interval file. A value of zero indicates no upper limit. [0]

-d, --datatype str Type of data to produce for the heatmaps. Valid options are raw, fend (only fend corrections applied), distance (only distance-dependence signal removed), enrichment (both fend correction and distance-dependence signal removed), and expected (only predicted signal). [fend]

-y, --dynamically-bin Dynamically bin heatmap.

-x, --expansion-binsize int The size of bins, in base pairs, to group data into for expanding underpopulated bins. [10000]

-f, --minobservations int The minimum number of observed reads in a bin for it to be considered valid. [20]

-a, --search-distance int The furthest distance from the bin minpoint to expand bounds. If set to zero, there is no limit on expansion distance. [0]

-v, --remove-failed If a non-zero 'search-distance' is given, it is possible for a bin not to meet the 'minobservations' criteria before stopping looking. If this occurs and 'remove-failed' is set, the observed and expected values for that bin are zero.

HiC Plotting Options:

-i, --image FILE Generate an image from the region or regions for which heatmap data is being calculated. [None]

-p, --pdf Format the image as a pdf. [None]

-r, --rotate Rotate the image 45 degrees so the chromosome axis is horizontal and only plot the triangle above this axis.

-t, --ticks Add coordinate ticks and labels to heatmap. This option can only be used if a pdf is requested.

-l, --legend Add a color scale bar corresponding to interaction strength. This option can only be used if a pdf is requested.

-n, --names Add chromosome names to the plot. This option can only be used if a pdf is requested.

-k, --keyword str Pass additional plotting options accepted by the `plotting` module. Arguments should be of the format KEYWORD=VALUE. This option can be passed multiple times. [None]

HiC Multi-Resolution Heatmap Options:

-t, --trans Calculate and include trans interactions in heatmaps.

-c, --chromosomes str A comma-separated list if chromosome names to include in the heatmaps. [all chromosomes]

-f, --minobservations int The minimum number of observed reads in a bin for it to be considered valid. [20]

-B, --maximum-binsize int The largest sized bin to use (minimum resolution) in, base pairs. [1280000]

-b, --minimum-binsize int The smallest sized bin to use (maximum resolution) in, base pairs. [10000]

-R, --maximum-trans-binsize int The largest sized bin to use (minimum resolution) for inter-chromosomal interactions, in base pairs. If not specified, this defaults to the value of the -B option. [use -B value]

-r, --minimum-trans-binsize int The smallest sized bin to use (maximum resolution) for inter-chromosomal interactions, in base pairs. If not specified, this defaults to the value of the -b option. [use -b value]

-m, --mid-binsize The smalled sized bin to use for binning the entire chromosome, in base pairs. This is used to balance memory usage vs. speed and does not affect the output. [40000]

-d, --datatype str Type of data to produce for the heatmaps. Valid options are raw, fend (only fend corrections applied), distance (only distance-dependence signal removed), enrichment (both fend correction and distance-dependence signal removed), and expected (only predicted signal). [fend]

Tutorial for 5C Classes

In order to perform analysis using the classes defined in `HiFive`, you must construct a series of objects, each one relying on the previous one. This tutorial will walk you through the process for doing a basic analysis of 5C data directly using the library of classes and functions.

11.1 Creating a `Fragment` object

In order to use `HiFive` to process 5C data, the first thing that you need to do is create a *Fragment* object. The *Fragment* object is an set of information contained in an HDF5 file. The object contains information about fragment sizes as determined by a series of boundary coordinates and orientations given in a BED formatted file, as well as an index for converting between string-based region names and numerical identifiers and any addition fragment features included in that bed file. The user may also include information about the genome and restriction enzyme, although these are optional and do not affect the functionality.

To create the *Fragment* object, you must provide a bed file in which each line contains the up- and downstream boundaries of a fragment and the strand to which this fragment belongs. Fragment names are also stored from the BED file and are used to connect mapped reads to fragments. Finally, groups of fragments can be separated into distinct regions which will be handled separately in downstream normalization and analysis. Fragments occurring on different chromosomes are always assigned to different regions. In addition, the user has the option of either explicitly specifying a list of regions or giving a minimum separation distance between fragments that is used to partition fragments into distinct regions.

To create a basic *Fragment* object, use the following command:

```
import hifive
fragment = hifive.Fragment(out_filename, mode='w')
fragment.load_fragments(bed_filename, genome_name='MM9', re_name='HindIII')
fragment.save()
```

In this case, the 'out_filename' specifies the location to save the *Fragment* object to. The 'bed_filename' contains the fragment boundaries, primer names, and strand information in BED format. The 'genome_name' and 're_name' are option strings that may be passed. In this case, the regions would be automatically determined using the default minimum distance between regions of 1 Mb. This could set by specifying a value with the keyword 'minregionspacing'.

In order to specify how to partition fragments into regions, we could use the following commands:

```
import hifive
fragment = hifive.Fragment(out_filename, mode='w')
fragment.load_fragments(bed_filename, regions=[['chr1', start1, stop1], ['chr2', start2, stop2]])
fragment.save()
```

This would create two regions that would include all of the fragments between coordinate values start1 - stop1 and start2 - stop2. When regions are explicitly given, the 'minregionspacing' variable is ignored.

11.2 Creating a `FiveCData` object

In order to create a 5C dataset, you first need to have created an appropriate *Fragment* object. You can create the *FiveCData* object either from a file containing primer pairs and their observed counts or directly from mapped data in BAM format. If using counts data to create the dataset, the format should be:

```
primer1   primer2 #_observed
```

where values are separated by tabs and '#_observed' is an integer. With either format the primer names must exactly match those in the BED file used to create the *Fragment* object.

To create the 5C dataset, you can run the following commands:

```
import hifive
data = hifive.FiveCData(out_filename, mode='w')
data.load_data_from_counts(fragment_filename, [counts1.txt, counts2.txt])
data.save()
```

In this case, 'out_filename' specifies the location to save the *FiveCData* object to. The 'fragment_filename' value is the location of the appropriate *Fragment* object. Multiple files containing counts data may be passed to the function as a list or, if only a single counts file is needed, it may be passed as a string. In order to load data from a set of BAM files, a similar procedure is used:

```
import hifive
data = hifive.FiveCDataset(out_filename, mode='w')
data.load_data_from_bam(fragment_filename, [[bam_file1, bam_file2]])
data.save()
```

In this case, the only difference is that pairs of file names corresponding to the two mapped read ends are passed as lists. Like the function for counts data, if only a single pair of files is needed, it may be passed as a list (not nested).

> Note: The *FiveCData* object can now be used by multiple analyses of this sample and does not need to be created separately for each one.

11.3 Creating a `FiveC` project object

The 5C project object, *FiveC*, contains links to a *FiveCData* and *Fragment* object, information about which fragments to include in the analysis, model parameters, and learned model values. This is the standard way of working with 5C data in HiFive and this object will be used for learning the model, extracting portions of data, plotting, and downstream analysis.

To create a *FiveC* object, you can use the following commands:

```
import hifive
fivec = hifive.FiveC(out_filename, 'w')
fivec.load_data(data_filename)
fivec.save()
```

In this case, 'out_filename' specifies the location to save the *FiveC* object to. The 'data_filename' value is the location of the appropriate *FiveCData* object.

> **Warning:** Because data and fragment data are stored in their own objects, each object keeps track of the location of its dependents through relative file names. This means that links between them will break if the relative pathway is changed.

11.4 Filter 5C fragments

Prior to modeling the data, you need to filter out fragments that have few valid reads mapped to them. `HiFive` uses an iterative filtering approach such that only when all fragments satisfy a user-defined minimum number of valid interactions does the filtering process cease.

To filter fragments, you can use the following commands:

```
import hifive
fivec = hifive.FiveC(fivec_filename)
fivec.filter_fragments(mininteractions=25)
fivec.save()
```

In this case, 'fivec_filename' is a previously saved `FiveC` analysis object. No value was passed to mode, since it defaults to 'r' for read. This loads the data from a previously created `FiveCData` object. In order for changes to be kept to a `FiveC` object, it must be written to file using the save command.

11.5 Find 5C distance function

`HiFive` approximates the distance-signal relationship using a power-law regression such that the log of the distance between the midpoints of two fragments and the log of their observed interactions. To do an initial estimate of this function, you can use the following command:

```
fivec.find_distance_parameters()
```

11.6 Learn 5C normalization parameters

11.6.1 Using the probability algorithm

In order to learn the correction model for 5C data using the probability algorithm, `HiFive` uses a bactracking line gradient descent. In addition, `HiFive` limits which interactions it uses to learn the model parameters to those that fall within a user-specified maximum interaction distance.

To learn 5C corrections using the probability approach, you can use the following command:

```
fivec.find_probability_fragment_corrections(mindistance=50000,
                                            maxdistance=0,
                                            learningstep=0.5,
                                            max_iterations=1000,
                                            minchange=0.005,
                                            regions=[0, 1, 2])
```

In the above call, 'mindistance' is set to 50 kb, indicating that interactions shorter than that distance are no useed in calculating correction values. maxdistance' is set to zero, indicating that there is no upper limit on interaction distance to be used for learning model parameters. TThe 'learningstep' specifies how quickly to scale down the step value if the current try doesn't meet the arjimo learning criterion. The 'max_iterations' specifies a limit for how long to run the

learning process for. 'minchange' is the stopping threshold such that if all absolute gradient values are below this the learning terminates early. Finally, the 'regions' parameter specifies that we only want to learn corrections for regions 0 - 3. Not specifying a value for this parameter would default to including all regions.

11.6.2 Using the express algorithm

`HiFive` also offers a matrix-balancing approach for learning correction values. The primary differences to the probability model from the user's perspective are a single learning phase and a lack of learning rate.

To learn 5C corrections using the approximation approach, you can use the following command:

```
fivec.find_express_fragment_corrections(iterations=1000,
                                        mindistance=50000,
                                        maxdistance=0,
                                        remove_distance=True)
```

In the above call, the 'remove_distance' argument specifies whether to remove the distance-dependent portion of the signal prior to approximating correction values. For best results, this should set to true (its default value).

11.6.3 Using the binning algorithm

`HiFive` also offers a fragment characteristic-based approach adapted from the learning model used by HiCPipe. This algorithm takes a list of features to be partitioned and a number of bins to partition them into and learns correction values associated with each partition based on a log-normal distribution of non-zero interactions corrected for distance-dependence.

To learn 5C corrections using the binning approach, you can use the following command:

```
fivec.find_binning_fragment_corrections(max_iterations=1000,
                                        mindistance=50000,
                                        maxdistance=0,
                                        num_bins=[10, 10],
                                        model=['len', 'gc'],
                                        parameters=['even', 'fixed-const'],
                                        usereads='cis',
                                        learning_threshold=1.0)
```

Unlike the other two learning algorithms, this approach caps the learning iterations using 'max_iterations' and provides a means of early termination. This is done with the 'learning_threhold' parameter, which specifies that if the change in log-likelihood drops below 1.0, then cease iterating. The 'model', 'num_bins', and 'parameters' values should consist of equal-length lists and describe the correction values that are to be learned. Here, we told HiFive to use the length and gc content (specified in our BED file) for each fragment. Each feature was partitioned into a number of bins specified in 'num_bins'. The partitioning of length was done to create bins containing equal numbers of fragments while the gc content was divided such that each bin spanned an equal portion of the characteristic's range. Finally, the '-const' suffix told HiFive not to optimize the values for gc content. The 'usereads' value 'cis' specified that only within-region interactions should be used to learn these correction values.

11.6.4 Chaining learning algorithms

Because they work in very different ways, `HiFive` allows the binning algorithm to be chained with either the probability or express algorithm. The learning algorithms can be run in either order and the corrections from the first algorithm are applied prior to learning corrections for the second algorithm. This can be done by using the 'precorrect' option as follows:

```
fivec.find_express_fragment_corrections(iterations=1000,
                                        mindistance=50000,
                                        maxdistance=0,
                                        remove_distance=True)
fivec.find_binning_fragment_corrections(max_iterations=1000,
                                        mindistance=50000,
                                        maxdistance=0,
                                        num_bins=[10],
                                        model=['len'],
                                        parameters=['even'],
                                        usereads='cis',
                                        learning_threshold=1.0,
                                        precorrect=True)
```

11.7 Generating a heatmap

In order to immediately make use of data, `HiFive` allows you to pull data from a region and create a heatmap. The data can be returned unbinned, binned using a fixed-width bin size, or binned using boundaries passed by the user. There are several options for the format the data can be passed back in. Please refer to the *cis_heatmap* function for more details. There are also several options for transformations to the data. These are used to remove the distance-dependence signal, fragment bias, both, or to return only the predicted signal. In this example, we'll get a set of data from an entire region binned into 10 Kb bins as follows:

```
heatmap = fivec.cis_heatmap(region=1,
                            binsize=10000,
                            arraytype='compact',
                            datatype='enrichment')
```

In the above call, 'enrichment' specifies to find the observed counts and expected counts, which includes the distance-dependence and fragment bias values. The observed counts are in the first index of the last dimension of the returned array, the expected counts are in the second index of the last dimension. 'compact' specifies a rectangular array where the first axis is the forward primers and the second axis is the reverse primers. 'region' refers to the region index given by `HiFive`. To find out more details about that region, we could do the following:

```
print fivec.frags['regions'][1]
```

This returns the region's index, chromosome, starting fragment, stopping fragment (first fragment outside the region), starting coordinate and stopping coordinate.

11.8 Accessing heatmap data

When a heatmap is generated, data are stored in an HDF5 dictionary, a binary file format that allows easy access through python. In order to access data from your heatmap, you can load it as follows:

```
import h5py
import numpy
print heatmap.keys()
heatmap = h5py.File(heatmap_file, 'r')
counts = heatmap['0.counts'][...]
expected = heatmap['0.expected'][...]
enrichment = numpy.zeros((counts.shape[0], 2), dtype=numpy.float32)
where = numpy.where(counts > 0)[0]
```

```
enrichment[where, 0] = numpy.log(counts[where] / expected[where])
enrichment[where, 1] = 1
```

Note that we used the 'r' option when opening the file with h5py. This ensures that we are in 'read' mode. You could also use 'a' for 'append' mode, which is the default. First we printed out the available dataset names in our heatmap file. These are all of the arrays that are accessible to us by calling them like any other key value in a dictionary. Next, in order to load data from the heatmap into memory rather than access it from the disk every time we refer to it, we use the '[...]' indexing call after pass the heatmap filestream the name of the data we want. In this case, we asked for the counts and expected values for region zero. In order to look at the enrichments, we took the log of the ratio of observed to expected values for each bin. However, there are likely bins that contain no observed counts which would give us a divide by zero error in the log function. So, we can use numpy's 'where' function to get a index list of places that match our criterion, in this case non-zero counts. Finally, we have made the enrichment array 2D so we can keep track of which bins are valid (nonzero counts). If we were looking at trans data, we would need one more dimension as the counts and expected arrays would be two-dimensional instead of one.

11.9 Plotting a heatmap

In order to visualize the heatmap we just produced, `HiFive` has several plotting functions that take different shaped arrays. The function called needs to match the array produced. However, in this case, the 5C compact array is compatible with the *plot_full_array* function, so we'll use that as follows:

```
img = hifive.plotting.plot_full_array(heatmap, symmetric_scaling=True)
img.save(out_fname)
```

In calling the function, we pass the heatmap and that would be sufficient. There are, however, additional options. For example, 'symmetric_scaling' specifies whether the color scale should be expanded to run from the minimum value to the maximum (False) or so that the maximum absolute value determine both upper and lower color bounds. The image returned is a PIL image of type 'png'.

CHAPTER 12

Tutorial for HiC Classes

In order to perform analysis using the classes defined in `HiFive`, you must construct a series of objects, each one relying on the previous one. This tutorial will walk you through the process for doing a basic analysis of HiC data directly using the library of classes and functions.

12.1 Creating a `Fend` object

In order to use `HiFive` to process HiC data, the first thing that you need to do is create a fragment-end (Fend) object. The *Fend* object is an set of information contained in an HDF5 file. The object contains information about the fragments created by digestion of a genome by a specific restriction enzyme (RE) as well as an index for converting between string-based chromosome names and numerical identifiers. The user may also include information about the genome and restriction enzyme, although these are optional and do not affect the functionality.

To create a *Fend* object, you must provide the location of RE fragments. This information is supplied in the form of either a 'fend' file with a format compatible with HiCPipe or a BED-formatted file containing fragment boundaries produced by RE digest or locations of the RE cut sites.

To create a basic *Fend* object, use the following command:

```
import hifive
fend = hifive.Fend(out_filename, mode='w')
fend.load_fends(RE_data_filename, genome_name='MM9', re_name='HindIII', format='bed')
fend.save()
```

In this case, the 'out_filename' specifies the location to save the *Fend* object to. The 'RE_data_filename' contains the RE fragment boundaries in one of the formats described above. The 'genome_name' and 're_name' are option strings that may be passed. The 'format' argument specifies that we are passing a BED file containing the fend data to load.

Note: The *Fend* object can now be used by any experiment that relies on the same genome / restriction enzyme combination and does not need to be created separately for different experiments or analyses.

12.2 Creating a `HiCData` object

In order to create a HiC dataset, you first need to have created an appropriate *Fend* object. You can create the `HiCData` object from a file containing fend indices and their observed counts, the format used with HiCPipe data (the 'mat' format), a text file containing pairs of chromosomes, coordinates, and strands (referred to from now on as a 'raw' file), or directly from mapped data in BAM format. If using raw coordinate data to create the dataset, the format should be:

```
chr1   coord1    strand1     chr2     coord2   strand2
```

where values are separated by tabs and strands are denoted by '+' or '-'. In addition to mapped reads, you need to provide a maximum insert size, i.e. the total acceptable length of a sequenced fragment as determined by the sum of each mapping location to its downstream RE cutsite.

To create the 5C dataset from a HiCPipe-compatible format, you can run the following commands:

```
import hifive
data = hifive.HiCData(out_filename, mode='w')
data.load_data_from_mat(fend_filename, data_filename)
data.save()
```

In this case, 'out_filename' specifies the location to save the *HiCData* object to. The 'fend_filename' value is the location of the appropriate *Fend* object. To maintain compatibility with HiCPipe-formatted 'mat' files, `HiFive` expects that fend and fragment numbering begin at index 1, not 0.

To create the 5C dataset from raw coordinate data, you can run the following commands:

```
import hifive
data = hifive.HiCData(out_filename, mode='w')
data.load_data_from_raw(fend_filename, [raw1.txt, raw2.txt], maxinsert=500)
data.save()
```

In this case, 'out_filename' specifies the location to save the `HiCData` object to. The 'fend_filename' value is the location of the appropriate *Fend* object. Multiple files containing paired-end mapped coordinates may be passed to the function as a list or, if only a single file is needed, it may be passed as a string.

In order to load data from a set of BAM files, a similar procedure is used:

```
import hifive
data = hifive.HiCData(out_filename, mode='w')
data.load_data_from_bam(fragment_filename,
  [[bam_file1_1, bam_prefix1_2], [bam_file2_1, bam_file2_2]],
  maxinsert=500)
data.save()
```

IIn this case, the only difference is that pairs of file names corresponding to the two mapped read ends are passed as lists. Like the function for counts data, if only a single pair of files is needed, it may be passed as a list (not nested).

Note: The `HiCData` object can now be used by multiple analyses of this sample and does not need to be created separately for each one.

12.3 Creating a `HiC` project object

The HiC project object, *HiC*, contains links to a HiCData and *Fend* object, information about which fends to include in the analysis, model parameters, and learned model values. This is the standard way of working with HiC data in `HiFive` and this object will be used for learning the model, extracting portions of data, plotting, and downstream analysis.

To create a *HiC* object, you can use the following commands:

```
import hifive
hic = hifive.HiC(out_filename, 'w')
hic.load_data(data_filename)
hic.save()
```

In this case, 'out_filename' specifies the location to save the *HiC* object to. The 'data_filename' value is the location of the appropriate data object.

> **Warning:** Because data and fragment data are stored in their own objects, each object keeps track of the location of its dependents through relative file names. This means that links between them will break if the relative pathway is changed.

12.4 Filter HiC fends

Prior to modeling the data, you need to filter out fends that have few valid reads mapped to them. `HiFive` uses an iterative filtering approach such that only when all fends satisfy a user-defined minimum number of valid interactions does the filtering process cease.

To filter fends, you can use the following commands:

```
import hifive
hic = hifive.HiC(hic_filename)
hic.filter_fends(mininteractions=25, mindistance=5000000)
hic.save()
```

In this case, 'hic_filename' is a previously saved *HiC* analysis object. No value was passed to mode, since it defaults to 'r' for read. This loads the data from a previously created HiCData object. In order for changes to be kept to a FiveC object, it must be written to file using the save command. The 'mindistance' argument specifies that only reads associated with interactions spanning that distance or more are counted for purposes of filtering fends.

12.5 Find HiC distance function

`HiFive` approximates the distance-signal relationship using a series of linear transitions between bin means of mean log interaction counts. Spanning from a user-defined minimum interaction distance up to the genome maximum interaction distance, the range is divided into equal-sized log distance bins. Values falling between bin midpoints are interpolated based on a linear transition between bins. To estimate this function, you can use the following command:

```
hic.find_distance_parameters(numbins=90,
                             minsize=200,
                             maxsize=0)
```

In this function call, the range of interaction sizes is being broken into 90 bins, 1 bin covering interactions <= 200 bp, and the other 89 spanning up to the maximum interaction distance with breaks evenly spaced in log space. The maximum of this range is set by 'maxsize', which can either be zero, as in this call, setting the maximum size equal to the longest interaction distance, or a positive integer value which would exclude any interaction distances greater than 'maxsize'.

Because this function involves scanning large amounts of data, it has been made to utilize MPI. To do so, you can use a scripts such as the following:

```
import hifive
from mpi4py import MPI

rank = MPI.COMM_WORLD.Get_rank()
hic = hifive.HiC(hic_filename)
hic.find_distance_means(numbins=90,
                        minsize=200,
                        maxsize=0)
```

```
if rank == 0:
  hic.save()
```

12.6 Learn HiC normalization parameters

12.6.1 Using the probability algorithm

In order to learn the correction model for HiC data using the probability algorithm, `HiFive` uses a bactracking line gradient descent. In addition, `HiFive` limits which interactions it uses to learn the model parameters to those that fall within a user-specified maximum interaction distance.

To learn HiC corrections using the modeling approach, you can use the following command:

```
hic.find_probability_fend_corrections(mindistance=5000000,
                                      learningstep=0.5,
                                      max_iterations=1000,
                                      minchange=0.0005)
```

In the above call, 'mindistance' indicates that interactions spanning less than 5 Mb are excluded from calculations. Setting this to zero would include all unfiltered cis interactions. The 'learningstep' specifies how quickly to scale down the step value if the current try doesn't meet the arjimo learning criterion. The 'max_iterations' specifies a limit for how long to run the learning process for. Finally, 'minchange' is the stopping threshold such that if all absolute gradient values are below this the learning terminates early.

Because of the large numbers of calculations involved in this function, it has been made to utilize MPI. To do so, you can use a scripts such as the following:

```
import hifive
from mpi4py import MPI

rank = MPI.COMM_WORLD.Get_rank()
hic = hifive.HiC(hic_filename)
hic.find_fend_corrections(mindistance=5000000,
                          learningstep=0.5,
                          max_iterations=1000,
                          minchange=0.0005)
if rank == 0:
  hic.save()
```

12.6.2 Using the express algorithm

`HiFive` also offers an express algorithm based on a matrix-balancing approach for learning correction values. The primary differences to the correction model from the user's perspective are a single learning phase and a lack of learning rate. The approximation learning approach can still recalculate the distance function parameters periodically.

To learn HiC corrections using the approximation approach, you can use the following command:

```
hic.find_express_fend_corrections(iterations=1000,
                                  mindistance=0,
                                  usereads='cis',
                                  remove_distance=True)
```

In the above call, 'mindistance' is used to exclude interaction distances shorter that the passed value. If this results in the exclusion of any reads, fends are refiltered using either the value passed under the keyword 'mininteractions' or, if that is not specified, the value passed the last time fends were filtered. The 'usereads' argument allows the user to base

the correction value approximation on 'cis' interactions, 'trans' interactions, or 'all'. Selecting 'trans' interactions will also result in a refiltering of fends to ensure that all of them are involved in sufficient interactions as described previously. The 'remove_distance' argument specifies whether to remove the distance-dependent portion of the signal prior to approximating correction values. For best results, this should set to true (its default value).

Although this function is much more computationally efficient, the calculation of the distance-dependence signal estimates can take time and so has been made to utilize the MPI environment when available as follows:

```
import hifive
from mpi4py import MPI

rank = MPI.COMM_WORLD.Get_rank()
hic = hifive.HiC(hic_filename)
hic.find_express_fend_corrections(iterations=1000,
                                  mindistance=0,
                                  usereads='cis',
                                  remove_distance=True)
if rank == 0:
  hic.save()
```

12.6.3 Using the binning algorithm

`HiFive` also offers a fend characteristic-based approach adapted from the learning model used by HiCPipe. This algorithm takes a list of features to be partitioned and a number of bins to partition them into and learns correction values associated with each partition based on a binomial distribution of binary data (observed / not observed).

To learn HiC corrections using the binning approach, you can use the following command:

```
hic.find_binning_fragment_corrections(max_iterations=1000,
                                      mindistance=5000000,
                                      maxdistance=0,
                                      num_bins=[10, 10],
                                      model=['len', 'gc'],
                                      parameters=['even', 'fixed-const'],
                                      usereads='cis',
                                      learning_threshold=1.0)
```

Unlike the other two learning algorithms, this approach caps the learning iterations using 'max_iterations' and provides a means of early termination. This is done with the 'learning_threhold' parameter, which specifies that if the change in log-likelihood drops below 1.0, then cease iterating. The 'model', 'num_bins', and 'parameters' values should consist of equal-length lists and describe the correction values that are to be learned. Here, we told HiFive to use the length and gc content (specified in our BED file) for each fend. Each feature was partitioned into a number of bins specified in 'num_bins'. The partitioning of length was done to create bins containing equal numbers of fends while the gc content was divided such that each bin spanned an equal portion of the characteristic's range. Finally, the '-const' suffix told HiFive not to optimize the values for gc content. The 'usereads' value 'cis' specified that only intra-chromosomal interactions should be used to learn these correction values.

Although learning the correction values with this algorithm is much more computationally efficient, calculating the number of observations and possible observations per bin must span a large number of fend combinations so this function has been made to utilize the MPI environment when available as follows:

```
import hifive
from mpi4py import MPI

rank = MPI.COMM_WORLD.Get_rank()
hic = hifive.HiC(hic_filename)
hic.find_binning_fragment_corrections(max_iterations=1000,
```

```
                                    mindistance=5000000,
                                    maxdistance=0,
                                    num_bins=[10, 10],
                                    model=['len', 'gc'],
                                    parameters=['even', 'fixed-const'],
                                    usereads='cis',
                                    learning_threshold=1.0)
if rank == 0:
  hic.save()
```

12.6.4 Chaining learning algorithms

Because they work in very different ways, `HiFive` allows the binning algorithm to be chained with either the probability or express algorithm. The binning learning algorithm must be performed first and the corrections are applied prior to learning corrections with the second algorithm. This can be done by using the 'precorrect' option as follows:

```
fivec.find_binning_fragment_corrections(max_iterations=1000,
                                        mindistance=5000000,
                                        maxdistance=0,
                                        num_bins=[20],
                                        model=['len','gc'],
                                        parameters=['even','even'],
                                        usereads='cis',
                                        learning_threshold=1.0)
fivec.find_express_fragment_corrections(iterations=1000,
                                        mindistance=5000000,
                                        maxdistance=0,
                                        remove_distance=True,
                                        precorrect=True)
```

12.7 Generating a heatmap

In order to immediately make use of data, `HiFive` allows you to pull data from a region and create a heatmap. The data can be returned unbinned, binned using a fixed-width bin size, or binned using boundaries passed by the user. There are several options for the format the data can be passed back in. Please refer to the *cis_heatmap* function for more details. There are also several options for transformations to the data. These are used to remove the distance-dependence signal, fend bias, both, or to return only the predicted signal. In this example, we'll get a portion of chromosome 1 binned into 10 Kb bins as follows:

```
heatmap = hic.cis_heatmap(chrom='1',
                          start=1000000
                          stop=3000000
                          binsize=10000,
                          arraytype='upper',
                          datatype='enrichment')
```

In the above call, All valid possible interactions were queried from chromosome 1 between 1000000 and 3000000. For valid interactions that had no observation, an expected value was still added to the bin. 'enrichment' specifies to find the observed counts and expected counts, which includes the distance-dependence and fend bias values. The observed counts are in the first index of the last dimension of the returned array, the expected counts are in the second index of the last dimension. 'Upper' specifies a row-major upper triangle array (all values above the matrix diagonal flattened).

12.8 Accessing heatmap data

When a heatmap is generated, data are stored in an HDF5 dictionary, a binary file format that allows easy access through python. In order to access data from your heatmap, you can load it as follows:

```
import h5py
import numpy
heatmap = h5py.File(heatmap_file, 'r')
print heatmap.keys()
counts = heatmap['0.counts'][...]
expected = heatmap['0.expected'][...]
enrichment = numpy.zeros((counts.shape[0], 2), dtype=numpy.float32)
where = numpy.where(counts > 0)[0]
enrichment[where, 0] = numpy.log(counts[where] / expected[where])
enrichment[where, 1] = 1
```

Note that we used the 'r' option when opening the file with h5py. This ensures that we are in 'read' mode. You could also use 'a' for 'append' mode, which is the default. First we printed out the available dataset names in our heatmap file. These are all of the arrays that are accessible to us by calling them like any other key value in a dictionary. Next, in order to load data from the heatmap into memory rather than access it from the disk every time we refer to it, we use the '[...]' indexing call after pass the heatmap filestream the name of the data we want. In this case, we asked for the counts and expected values for region zero. In order to look at the enrichments, we took the log of the ratio of observed to expected values for each bin. However, there are likely bins that contain no observed counts which would give us a divide by zero error in the log function. So, we can use numpy's 'where' function to get a index list of places that match our criterion, in this case non-zero counts. Finally, we have made the enrichment array 2D so we can keep track of which bins are valid (nonzero counts). If we were looking at trans data, we would need one more dimension as the counts and expected arrays would be two-dimensional instead of one.

12.9 Plotting a heatmap

In order to visualize the heatmap we just produced, `HiFive` has several plotting functions that take different shaped arrays. The function called needs to match the array produced. In this case, we produced an upper array which is compatible with the *`hifive.plotting.plot_upper_array()`* function, so we'll use that as follows:

```
img = hifive.plotting.plot_upper_array(heatmap, symmetric_scaling=True)
img.save(out_fname)
```

In calling the function, we pass the heatmap and that would be sufficient. There are, however, additional options. For example, 'symmetric_scaling' specifies whether the color scale should be expanded to run from the minimum value to the maximum (False) or so that the maximum absolute value determine both upper and lower color bounds. The image returned is a `PIL` image of type 'png'.

12.10 Creating a multi-resolution heatmap

An Alternative to the standard heatmap is a HiFive-specific filetype called a *Multi-Resolution Heatmap* or MRH. In order to create this compact heatmap file, we can use the built-in *`HiC`* function as follows:

```
hic.load( project_filename )
hic.write_multiresolution_heatmap(out_fname, datatype='fend', maxbinsize=1280000, minbinsize=5000, i
```

This call would create an MRH file under the name specified in 'out_fname'. Data would cover all intra-chromosomal interactions and, because we passed 'True' to the 'includetrans' argument, all of the inter-chromosomal interactions

as well. Only bins with at least 5 reads would be included in the heatmaps because of the value passed to 'minobservations'. All resolutions from binsizes of 1.28Mb to 5Kb would be heatmapped in steps of 2X (i.e. 5Kb, 10Kb, 20Kb, etc). This imposes a limitation such that minbinsize and maxbinsize must differ from each other by an integer power of two.

In order to make use of this MRH file, we could either visualize it in Galaxy or use the stand-alone program 'fetch_mrh_data' included with HiFive. For example if we wanted to get data from chromosome 1 between 10Mb and 20Mb including all resolution data down to 10Kb, we could call the program as follows:

```
> fetch_mrh_data -c 1 -s 10000000 -e 20000000 -R 10000 mrh_fname img_fname
```

This would pull data from the MRH file 'mrh_fname' and plot it as an image in the file 'img_fname'. For specifics of the 'fetch_mrh_data' options, see *Standalone MRH program*.

CHAPTER 13

HiFive's API

Contents:

13.1 The Fragment class

A class for handling 5C fragment information.

class hifive.fragment.**Fragment** (*filename*, *mode='r'*, *silent=False*)

This class handles restriction enzyme digest-generated fragment data for 5C experiments.

The Fragment class contains all of the genomic information for a 5C experiment, including fragment locations and orientations, chromosome name mapping, and region locations and indices.

Note: This class is also available as hifive.Fragment

When initialized, this class creates an h5dict in which to store all data associated with this object.

Parameters

- **filename** (*str.*) – The file name of the h5dict. This should end with the suffix '.hdf5'
- **mode** (*str.*) – The mode to open the h5dict with. This should be 'w' for creating or overwriting an h5dict with name given in filename.
- **silent** (*bool.*) – Indicates whether to print information about function execution for this object.

Returns *Fragment* class object.

Attributes

- **file** (*str.*) - A string containing the name of the file passed during object creation for saving the object to.
- **silent** (*bool.*) - A boolean indicating whether to suppress all of the output messages.
- **history** (*str.*) - A string containing all of the commands executed on this object and their outcome.

load()

Load fragment data from h5dict specified at object creation.

Any call of this function will overwrite current object data with values from the last *save()* call.

Returns None

load_fragments(*filename*, *genome_name=None*, *re_name=None*, *regions=[]*, *minregionspacing=1000000*)

Parse and store fragment data from a bed for a 5C assay file into an h5dict.

Parameters

- **filename** (*str.*) – A file name to read restriction fragment data from. This should be a BED file containing fragment boundaries for all probed fragments and primer names that match those used for read mapping.
- **genome_name** (*str.*) – The name of the species and build. Optional.
- **re_name** (*str.*) – The name of the restriction enzyme used to produce the fragment set. Optional.
- **regions** (*list*) – User-defined partitioning of fragments into different regions. This argument should be a list of lists containing the chromosome, start, and stop coordinates for each region.
- **minregionspacing** (*int.*) – If 'regions' is not defined, this is used to parse regions by inserting breaks where fragments are spaced apart greater than this value.

Returns None

Attributes

- **chromosomes** (*ndarray*) - A numpy array containing chromosome names as strings. The position of the chromosome name in this array is referred to as the chromosome index.
- **fragments** (*ndarray*) - A numpy array of length N where N is the number of fragments and containing the fields 'chr', 'start', 'stop', 'mid', 'strand', 'region', and 'name'. With the exception of the 'name' field which is of type string, all of these are of type int32. The 'chr' and 'region' fields contain the indices of the chromosome and region, respectively. If the bed file used to create the Fragment object contains additional columns, these features are also included as fields with names corresponding to the bed header names. These additional fields are of type float32. Fragments are sorted by chromosome (the order in the 'chromosomes' array) and then by coordinates.
- **chr_indices** (*ndarray*) - A numpy array with a length of the number of chromosomes in 'chromosomes' + 1. This array contains the first position in 'fragments' for the chromosome in the corresponding position in the 'chromosomes' array. The last position in the array contains the total number of fragments.
- **regions** (*ndarray*) - A numpy array of length equal to the number of regions a containing the fields 'index', 'chromosome', 'start_frag', 'stop_frag', 'start' and 'stop'. Except for 'chromosome' which is a string, all fields are of type int32.

save()

Save fragment data to h5dict.

Returns None

13.2 The Fend class

A class for handling HiC fend information.

class hifive.fend.**Fend**(*filename*, *mode='r'*, *silent=False*)

This class handles restriction enzyme digest-generated fragment data for HiC experiments.

This class stores a list of chromosomes, a dictionary for converting from chromosome label to integer and back, fragment starts, stops, and chromosome number in an h5dict.

Note: This class is also available as hifive.Fend

When initialized, this class creates an h5dict in which to store all data associated with this object.

Parameters

- **filename** (*str.*) – The file name of the h5dict. This should end with the suffix '.hdf5'
- **mode** (*str.*) – The mode to open the h5dict with. This should be 'w' for creating or overwriting an h5dict with name given in filename.
- **silent** (*bool.*) – Indicates whether to print information about function execution for this object.

Returns `Fend` class object

Attributes

- **file** (*str.*) - A string containing the name of the file passed during object creation for saving the object to.
- **silent** (*bool.*) - A boolean indicating whether to suppress all of the output messages.
- **history** (*str.*) - A string containing all of the commands executed on this object and their outcome.

load()

Load fend data from h5dict specified at object creation.

Any call of this function will overwrite current object data with values from the last `save()` call.

Returns None

load_fends (*filename*, *genome_name=None*, *re_name=None*, *format=None*)

Parse and store fend data in h5dict.

Parameters

- **filename** (*str.*) – A file name to read restriction fragment data from. The file may be a 'mat' file compatible with HiCPipe, or a BED file containing RE fragment boundaries or cutsites.
- **genome_name** (*str.*) – The name of the species and build. Optional.
- **re_name** (*str.*) – The name of the restriction enzyme used to produce the fragment set. Optional.
- **format** (*str.*) – Format of the input file. If not specified, it will be inferred from the file extension. Optional.

Returns None

Attributes

- **chromosomes** (*ndarray*) - A numpy array containing chromosome names as strings. The position of the chromosome name in this array is referred to as the chromosome index.
- **fends** (*ndarray*) - A numpy array of length N where N is the number of fends and containing the fields 'chr', 'start', 'stop', and 'mid'. All of these are of type int32. The 'chr' field contains the index of the chromosome. If the bed file or fend file used to create the Fend object contains additional columns, these features are also included as fields with names corresponding to the header names. These additional fields are of type float32. If produced

from a bed file, fends are sorted by chromosome (the order in the 'chromosomes' array) and then by coordinates.

- **chr_indices** (*ndarray*) - A numpy array with a length of the number of chromosomes in 'chromosomes' + 1. This array contains the first position in 'fragments' for the chromosome in the corresponding position in the 'chromosomes' array. The last position in the array contains the total number of fragments.
- **genome_name** (*str.*) - A string (or None if not passed as argument) of the genome from which the fends originated.
- **re_name** (*str.*) - A string (or None if not passed as argument) of the restriction enzyme used to produce the fends.

save()

Save fend data to h5dict.

Returns None

13.3 The FiveCData class

A class for handling 5C read data.

class hifive.fivec_data.**FiveCData** (*filename*, *mode='r'*, *silent=False*)

This class handles interaction count data for 5C experiments.

The FiveCData class contains all of the interaction information for a 5C experiment, including pairs of fragment indices and their associated counts.

Note: This class is also available as hifive.FiveCData

When initialized, this class creates an h5dict in which to store all data associated with this object.

Parameters

- **filename** (*str.*) – The file name of the h5dict. This should end with the suffix '.hdf5'
- **mode** (*str.*) – The mode to open the h5dict with. This should be 'w' for creating or overwriting an h5dict with name given in filename.
- **silent** (*bool.*) – Indicates whether to print information about function execution for this object.

Returns *FiveCData* class object.

Attributes

- **file** (*str.*) A string containing the name of the file passed during object creation for saving the object to.
- **silent** (*bool.*) - A boolean indicating whether to suppress all of the output messages.
- **history** (*str.*) - A string containing all of the commands executed on this object and their outcomes.

load()

Load data from h5dict specified at object creation.

Any call of this function will overwrite current object data with values from the last *save()* call.

Returns None

load_data_from_bam (*fragfilename*, *filelist*)
Read interaction counts from pairs of BAM files and place in h5dict.

Parameters

- **fragfilename** (*str.*) – This specifies the file name of the `Fragment` object to associate with the dataset.
- **filelist** (*list*) – A list containing lists of paired read end files.

Returns None

Attributes

- **fragfilename** (*str.*) - A string containing the relative path of the fragment file.
- **cis_data** (*ndarray*) - A numpy array of type int32 and shape N x 3 where N is the number of valid non-zero intra-regional fragment pairings observed in the data. The first column contains the fragment index (from the 'fragments' array in the Fragment object) of the upstream fragment, the second column contains the idnex of the downstream fragment, and the third column contains the number of reads observed for that fragment pair.
- **cis_indices** (*ndarray*) - A numpy array of type int64 and a length of the number of fragments + 1. Each position contains the first entry for the correspondingly-indexed fragment in the first column of 'cis_data'. For example, all of the downstream cis interactions for the fragment at index 5 in the Fragment object 'fragments' array are in cis_data[cis_indices[5]:cis_indices[6], :].
- **trans_data** (*ndarray*) - A numpy array of type int32 and shape N x 3 where N is the number of valid non-zero inter-regional fragment pairings observed in the data. The first column contains the fragment index (from the 'fragments' array in the Fragment object) of the upstream fragment (upstream also refers to the lower indexed chromosome in this context), the second column contains the idnex of the downstream fragment, and the third column contains the number of reads observed for that fragment pair.
- **trans_indices** (*ndarray*) - A numpy array of type int64 and a length of the number of fragments + 1. Each position contains the first entry for the correspondingly-indexed fragment in the first column of 'trans_data'. For example, all of the downstream trans interactions for the fragment at index 5 in the Fragment object 'fragments' array are in cis_data[cis_indices[5]:cis_indices[6], :].
- **frags** (*filestream*) - A filestream to the hdf5 Fragment file such that all saved Fragment attributes can be accessed through this class attribute.

When data is loaded the 'history' attribute is updated to include the history of the Fragment file that becomes associated with it.

load_data_from_counts (*fragfilename*, *filelist*)
Read interaction counts from a text file(s) and place in h5dict.

Parameters

- **fragfilename** (*str.*) – This specifies the file name of the `Fragment` object to associate with the dataset.
- **filelist** (*list*) – A list containing all of the file names of counts text files to be included in the dataset. If only one file is needed, this may be passed as a string.

Returns None

Attributes

- **fragfilename** (*str.*) - A string containing the relative path of the fragment file.

- **cis_data** (*ndarray*) - A numpy array of type int32 and shape N x 3 where N is the number of valid non-zero intra-regional fragment pairings observed in the data. The first column contains the fragment index (from the 'fragments' array in the Fragment object) of the upstream fragment, the second column contains the idnex of the downstream fragment, and the third column contains the number of reads observed for that fragment pair.
- **cis_indices** (*ndarray*) - A numpy array of type int64 and a length of the number of fragments + 1. Each position contains the first entry for the correspondingly-indexed fragment in the first column of 'cis_data'. For example, all of the downstream cis interactions for the fragment at index 5 in the Fragment object 'fragments' array are in cis_data[cis_indices[5]:cis_indices[6], :].
- **trans_data** (*ndarray*) - A numpy array of type int32 and shape N x 3 where N is the number of valid non-zero inter-regional fragment pairings observed in the data. The first column contains the fragment index (from the 'fragments' array in the Fragment object) of the upstream fragment (upstream also refers to the lower indexed chromosome in this context), the second column contains the index of the downstream fragment, and the third column contains the number of reads observed for that fragment pair.
- **trans_indices** (*ndarray*) - A numpy array of type int64 and a length of the number of fragments + 1. Each position contains the first entry for the correspondingly-indexed fragment in the first column of 'trans_data'. For example, all of the downstream trans interactions for the fragment at index 5 in the Fragment object 'fragments' array are in cis_data[cis_indices[5]:cis_indices[6], :].
- **frags** (*ndarray*) - A filestream to the hdf5 Fragment file such that all saved Fragment attributes can be accessed through this class attribute.

When data is loaded the 'history' attribute is updated to include the history of the Fragment file that becomes associated with it.

save()

Save analysis parameters to h5dict.

Returns None

13.4 The HiCData class

A class for handling HiC read data.

class hifive.hic_data.**HiCData**(*filename*, *mode='r'*, *silent=False*)

This class handles interaction count data for HiC experiments.

This class stores mapped paired-end reads, indexing them by fend-end (fend) number, in an h5dict.

Note: This class is also available as hifive.HiCData

When initialized, this class creates an h5dict in which to store all data associated with this object.

Parameters

- **filename** (*str.*) – The file name of the h5dict. This should end with the suffix '.hdf5'
- **mode** (*str.*) – The mode to open the h5dict with. This should be 'w' for creating or overwriting an h5dict with name given in filename.
- **silent** (*bool.*) – Indicates whether to print information about function execution for this object.

Returns *HiCData* class object.

Attributes

- **file** (*str.*) A string containing the name of the file passed during object creation for saving the object to.
- **silent** (*bool.*) - A boolean indicating whether to suppress all of the output messages.
- **history** (*str.*) - A string containing all of the commands executed on this object and their outcomes.

export_to_mat (*outfilename*)

Write reads loaded in data object to text file in `HiCPipe`-compatible 'mat' format.

Parameters **outfilename** (*str.*) – Specifies the file to save data in.

Returns None

load ()

Load data from h5dict specified at object creation.

Any call of this function will overwrite current object data with values from the last *save()* call.

Returns None

load_data_from_bam (*fendfilename*, *filelist*, *maxinsert*)

Read interaction counts from pairs of BAM-formatted alignment file(s) and place in h5dict.

Parameters

- **fendfilename** (*str.*) – This specifies the file name of the `Fend` object to associate with the dataset.
- **filelist** (*list of mapped sequencing runs. Each run should be a list of the first and second read end bam files ([[run1_1, run1_2], [run2_1, run2_2]...])*) – A list containing lists of paired end bam files. If only one pair of files is needed, the list may contain both file path strings.
- **maxinsert** (*int.*) – A cutoff for filtering paired end reads whose total distance to their respective restriction sites exceeds this value.

Returns None

Attributes

- **fendfilename** (*str.*) - A string containing the relative path of the fend file.
- **cis_data** (*ndarray*) - A numpy array of type int32 and shape N x 3 where N is the number of valid non-zero intra-chromosomal fend pairings observed in the data. The first column contains the fend index (from the 'fends' array in the fend object) of the upstream fend, the second column contains the idnex of the downstream fend, and the third column contains the number of reads observed for that fend pair.
- **cis_indices** (*ndarray*) - A numpy array of type int64 and a length of the number of fends + 1. Each position contains the first entry for the correspondingly-indexed fend in the first column of 'cis_data'. For example, all of the downstream cis interactions for the fend at index 5 in the fend object 'fends' array are in cis_data[cis_indices[5]:cis_indices[6], :].
- **trans_data** (*ndarray*) - A numpy array of type int32 and shape N x 3 where N is the number of valid non-zero inter-chroosomal fend pairings observed in the data. The first column contains the fend index (from the 'fends' array in the fend object) of the upstream fend (upstream also refers to the lower indexed chromosome in this context), the second

column contains the index of the downstream fend, and the third column contains the number of reads observed for that fend pair.

- **trans_indices** (*ndarray*) - A numpy array of type int64 and a length of the number of fends + 1. Each position contains the first entry for the correspondingly-indexed fend in the first column of 'trans_data'. For example, all of the downstream trans interactions for the fend at index 5 in the fend object 'fends' array are in cis_data[cis_indices[5]:cis_indices[6], :].
- **frags** (*ndarray*) - A filestream to the hdf5 fend file such that all saved fend attributes can be accessed through this class attribute.
- **maxinsert** (*int.*) - An interger denoting the maximum included distance sum between both read ends and their downstream RE site.

When data is loaded the 'history' attribute is updated to include the history of the fend file that becomes associated with it.

`load_data_from_mat` (*fendfilename*, *filename*, *maxinsert=0*)

Read interaction counts from a `HiCPipe`-compatible 'mat' text file and place in h5dict.

Parameters

- **`fendfilename`** (*str.*) – This specifies the file name of the `Fend` object to associate with the dataset.
- **`filename`** (*str.*) – File name of a 'mat' file containing fend pair and interaction count data.
- **`maxinsert`** (*int.*) – A cutoff for filtering paired end reads whose total distance to their respective restriction sites exceeds this value.

Returns None

Attributes

- **fendfilename** (*str.*) - A string containing the relative path of the fend file.
- **cis_data** (*ndarray*) - A numpy array of type int32 and shape N x 3 where N is the number of valid non-zero intra-chromosomal fend pairings observed in the data. The first column contains the fend index (from the 'fends' array in the fend object) of the upstream fend, the second column contains the idnex of the downstream fend, and the third column contains the number of reads observed for that fend pair.
- **cis_indices** (*ndarray*) - A numpy array of type int64 and a length of the number of fends + 1. Each position contains the first entry for the correspondingly-indexed fend in the first column of 'cis_data'. For example, all of the downstream cis interactions for the fend at index 5 in the fend object 'fends' array are in cis_data[cis_indices[5]:cis_indices[6], :].
- **trans_data** (*ndarray*) - A numpy array of type int32 and shape N x 3 where N is the number of valid non-zero inter-chroosomal fend pairings observed in the data. The first column contains the fend index (from the 'fends' array in the fend object) of the upstream fend (upstream also refers to the lower indexed chromosome in this context), the second column contains the index of the downstream fend, and the third column contains the number of reads observed for that fend pair.
- **trans_indices** (*ndarray*) - A numpy array of type int64 and a length of the number of fends + 1. Each position contains the first entry for the correspondingly-indexed fend in the first column of 'trans_data'. For example, all of the downstream trans interactions for the fend at index 5 in the fend object 'fends' array are in cis_data[cis_indices[5]:cis_indices[6], :].
- **frags** (*ndarray*) - A filestream to the hdf5 fend file such that all saved fend attributes can be accessed through this class attribute.

- **maxinsert** (*int.*) - An interger denoting the maximum included distance sum between both read ends and their downstream RE site.

When data is loaded the 'history' attribute is updated to include the history of the fend file that becomes associated with it.

load_data_from_raw (*fendfilename*, *filelist*, *maxinsert*)
Read interaction counts from a text file(s) and place in h5dict.

Files should contain both mapped ends of a read, one read per line, separated by tabs. Each line should be in the following format:

```
chromosome1    coordinate1  strand1   chromosome2    coordinate2  strand2
```

where strands are given by the characters '+' and '-'.

Parameters

- **fendfilename** (*str.*) – This specifies the file name of the `Fend` object to associate with the dataset.
- **filelist** (*list*) – A list containing all of the file names of mapped read text files to be included in the dataset. If only one file is needed, this may be passed as a string.
- **maxinsert** (*int.*) – A cutoff for filtering paired end reads whose total distance to their respective restriction sites exceeds this value.

Returns None

Attributes

- **fendfilename** (*str.*) - A string containing the relative path of the fend file.
- **cis_data** (*ndarray*) - A numpy array of type int32 and shape N x 3 where N is the number of valid non-zero intra-chromosomal fend pairings observed in the data. The first column contains the fend index (from the 'fends' array in the fend object) of the upstream fend, the second column contains the idnex of the downstream fend, and the third column contains the number of reads observed for that fend pair.
- **cis_indices** (*ndarray*) - A numpy array of type int64 and a length of the number of fends + 1. Each position contains the first entry for the correspondingly-indexed fend in the first column of 'cis_data'. For example, all of the downstream cis interactions for the fend at index 5 in the fend object 'fends' array are in cis_data[cis_indices[5]:cis_indices[6], :].
- **trans_data** (*ndarray*) - A numpy array of type int32 and shape N x 3 where N is the number of valid non-zero inter-chroosomal fend pairings observed in the data. The first column contains the fend index (from the 'fends' array in the fend object) of the upstream fend (upstream also refers to the lower indexed chromosome in this context), the second column contains the index of the downstream fend, and the third column contains the number of reads observed for that fend pair.
- **trans_indices** (*ndarray*) - A numpy array of type int64 and a length of the number of fends + 1. Each position contains the first entry for the correspondingly-indexed fend in the first column of 'trans_data'. For example, all of the downstream trans interactions for the fend at index 5 in the fend object 'fends' array are in cis_data[cis_indices[5]:cis_indices[6], :].
- **frags** (*ndarray*) - A filestream to the hdf5 fend file such that all saved fend attributes can be accessed through this class attribute.
- **maxinsert** (*int.*) - An interger denoting the maximum included distance sum between both read ends and their downstream RE site.

When data is loaded the 'history' attribute is updated to include the history of the fend file that becomes associated with it.

save()
Save analysis parameters to h5dict.

Returns None

13.5 The FiveC class

A class for handling 5C analysis.

class hifive.fivec.**FiveC**(*filename*, *mode='r'*, *silent=False*)
This is the class for handling 5C analysis.

This class relies on *Fragment* and *FiveCData* for genomic position and interaction count data. Use this class to perform filtering of fragments based on coverage, model fragment bias and distance dependence, and downstream analysis and manipulation. This includes binning of data, plotting of data, and statistical analysis.

Note: This class is also available as hifive.FiveC

When initialized, this class creates an h5dict in which to store all data associated with this object.

Parameters

- **filename** (*str.*) – The file name of the h5dict. This should end with the suffix '.hdf5'
- **mode** (*str.*) – The mode to open the h5dict with. This should be 'w' for creating or overwriting an h5dict with name given in filename.
- **silent** (*bool.*) – Indicates whether to print information about function execution for this object.

Returns *FiveC* class object.

Attributes

- **file** (*str.*) - A string containing the name of the file passed during object creation for saving the object to.
- **silent** (*bool.*) - A boolean indicating whether to suppress all of the output messages.
- **history** (*str.*) - A string containing all of the commands executed on this object and their outcome.
- **normalization** (*str.*) - A string stating which type of normalization has been performed on this object. This starts with the value 'none'.

In addition, many other attributes are initialized to the 'None' state.

cis_heatmap(*region*, *binsize=0*, *binbounds=None*, *start=None*, *stop=None*, *startfrag=None*, *stopfrag=None*, *datatype='enrichment'*, *arraytype='full'*, *skipfiltered=False*, *returnmapping=False*, *dynamically_binned=False*, *minobservations=0*, *searchdistance=0*, *expansion_binsize=0*, *removefailed=False*, *image_file=None*, ***kwargs*)
Return a heatmap of cis data of the type and shape specified by the passed arguments.

This function returns a heatmap for a single region, bounded by either 'start' and 'stop' or 'startfrag' and 'stopfrag' ('start' and 'stop' take precedence). If neither is given, the complete region is included. The data in the array is determined by the 'datatype', being raw, fragment-corrected, distance-corrected, enrichment, or expected data. The array shape is given by 'arraytype' and can be compact (if unbinned), upper, or

full. See `fivec_binning` for further explanation of 'datatype' and 'arraytype'. If using dynamic binning ('dynamically_binned' is set to True), 'minobservations', 'searchdistance', 'expansion_binsize', and 'removefailed' are used to control the dynamic binning process. Otherwise these arguments are ignored.

Parameters

- **region** (*int.*) – The index of the region to obtain data from.
- **binsize** (*int.*) – This is the coordinate width of each bin. If 'binsize' is zero, unbinned data is returned.
- **binbounds** (*numpy array*) – An array containing start and stop coordinates for a set of user-defined bins. Any fragment not falling in a bin is ignored.
- **start** (*int.*) – The smallest coordinate to include in the array, measured from fragment midpoints. If both 'start' and 'startfrag' are given, 'start' will override 'startfrag'. If unspecified, this will be set to the midpoint of the first fragment for 'region'. Optional.
- **stop** (*int.*) – The largest coordinate to include in the array, measured from fragment midpoints. If both 'stop' and 'stopfrag' are given, 'stop' will override 'stopfrag'. If unspecified, this will be set to the midpoint of the last fragment plus one for 'region'. Optional.
- **startfrag** (*int.*) – The first fragment to include in the array. If unspecified and 'start' is not given, this is set to the first fragment in 'region'. In cases where 'start' is specified and conflicts with 'startfrag', 'start' is given preference. Optional
- **stopfrag** (*str.*) – The first fragment not to include in the array. If unspecified and 'stop' is not given, this is set to the last fragment in 'region' plus one. In cases where 'stop' is specified and conflicts with 'stopfrag', 'stop' is given preference. Optional.
- **datatype** (*str.*) – This specifies the type of data that is processed and returned. Options are 'raw', 'distance', 'fragment', 'enrichment', and 'expected'. Observed values are always in the first index along the last axis, except when 'datatype' is 'expected'. In this case, filter values replace counts. Conversely, if 'raw' is specified, unfiltered fends return value of one. Expected values are returned for 'distance', 'fend', 'enrichment', and 'expected' values of 'datatype'. 'distance' uses only the expected signal given distance for calculating the expected values, 'fragment' uses only fragment correction values, and both 'enrichment' and 'expected' use both correction and distance mean values.
- **arraytype** (*str.*) – This determines what shape of array data are returned in. Acceptable values are 'compact' (if unbinned), 'full', and 'upper'. 'compact' means data are arranged in a N x M x 2 array where N and M are the number of forward and reverse probe fragments, respectively. 'full' returns a square, symmetric array of size N x N x 2 where N is the total number of fragments or bins. 'upper' returns only the flattened upper triangle of a full array, excluding the diagonal of size (N * (N - 1) / 2) x 2, where N is the total number of fragments or bins.
- **skipfiltered** (*bool.*) – If True, all interaction bins for filtered out fragments are removed and a reduced-size array is returned.
- **returnmapping** (*bool.*) – If True, a list containing the data array and either a 1d array containing fragment numbers included in the data array if the array is not compact or two 1d arrays containin fragment numbers for forward and reverse fragments if the array is compact is return. Otherwise only the data array is returned.
- **dynamically_binned** (*bool.*) – If True, return dynamically binned data.
- **minobservations** (*int.*) – The fewest number of observed reads needed for a bin to counted as valid and stop expanding.

- **searchdistance** (*int.*) – The furthest distance from the bin minpoint to expand bounds. If this is set to zero, there is no limit on expansion distance.
- **expansion_binsize** (*int.*) – The size of bins to use for data to pull from when expanding dynamic bins. If set to zero, unbinned data is used.
- **removefailed** (*bool.*) – If a non-zero 'searchdistance' is given, it is possible for a bin not to meet the 'minobservations' criteria before stopping looking. If this occurs and 'removefailed' is True, the observed and expected values for that bin are zero.
- **image_file** (*str.*) – If a filename is specified, a PNG image file is written containing the heatmap data. Arguments for the appearance of the image can be passed as additional keyword arguments.

Returns Array in format requested with 'arraytype' containing data requested with 'datatype'. If returnmapping is True, a list is returned containined the requested data array and an array of associated positions (dependent on the binning options selected).

filter_fragments(*mininteractions=20*, *mindistance=0*, *maxdistance=0*)

Iterate over the dataset and remove fragments that do not have 'minobservations' using only unfiltered fragments and interactions falling with the distance limits specified.

In order to create a set of fragments that all have the necessary number of interactions, after each round of filtering, fragment interactions are retallied using only interactions that have unfiltered fragments at both ends.

Parameters

- **mininteractions** (*int.*) – The required number of interactions for keeping a fragment in analysis.
- **mindistance** (*int.*) – The minimum inter-fragment distance to be included in filtering.
- **maxdistance** (*int.*) – The maximum inter-fragment distance to be included in filtering. A value of zero indicates no maximum cutoff.

Returns None

find_binning_fragment_corrections(*mindistance=0, maxdistance=0, model=['gc', 'len'], num_bins=[10, 10], parameters=['even', 'even'], learning_threshold=1.0, max_iterations=100, usereads='cis', regions=[], precorrect=False*)

Using multivariate binning model, learn correction values for combinations of model parameter bins.

Parameters

- **mindistance** (*int.*) – The minimum inter-fend distance to be included in modeling.
- **maxdistance** (*int.*) – The maximum inter-fend distance to be included in modeling.
- **model** (*list*) – A list of fragment features to be used in model. Valid values are 'len' and any features included in the creation of the associated Fragment object.
- **num_bins** (*int.*) – The number of approximately equal-sized bins two divide model components into.
- **parameters** (*list*) – A list of types, one for each model parameter. Types can be either 'even' or 'fixed', indicating whether each parameter bin should contain approximately even numbers of interactions or be of fixed width spanning 1 / Nth of the range of the parameter's values, respectively. Parameter types can also have the suffix '-const' to indicate that the parameter should not be optimized.
- **remove_distance** (*bool.*) – Use distance dependence curve in prior probability calculation for each observation.

- **learning_threshold** (*float*) – The minimum change in log-likelihood needed to continue iterative learning process.
- **max_iterations** (*int.*) – The maximum number of iterations to use for learning model parameters.
- **usereads** (*str.*) – Specifies which set of interactions to use, 'cis', 'trans', and 'all'.
- **regions** (*list*) – A list of regions to calculate corrections for. If set as None, all region corrections are found.
- **precorrect** (*bool.*) – Use fragment-based corrections in expected value calculations, resulting in a chained normalization approach.

Returns None

Attributes

- **model_parameters** (*ndarray*) - A numpy array of strings containing model parameter names.
- **binning_num_bins** (*ndarray*) - A numpy array of type int32 containing the number of bins for each model parameter.
- **binning corrections** (*ndarray*) - A numpy array of type float32 and length equal to the sum of binning_num_bins * (binning_num_bins - 1) / 2. This array contains a 1D stack of correction values, ordered according to the parameter order in the 'model_parameters' attribute.
- **binning_correction_indices** (*ndarray*) - A numpy array of type int32 and length equal to the number of model parameters plus one. This array contains the first position in 'binning_corrections' for the first bin of the model parameter in the corresponding position in the 'model_parameters' array. The last position in the array contains the total number of binning correction values.
- **binning_frag_indices** (*ndarray*) - A numpy array of type int32 and size N x M where M is the number of model parameters and N is the number of fragments. This array contains the binning index for each parameter for each fragment.

The 'normalization' attribute is updated to 'binning', 'probability-binning', or 'express-binning', depending on if the 'precorrect' option is selected and which normalization has been previously run.

find_distance_parameters()

Regress log counts versus inter-fragment distances to find slope and intercept values and then find the standard deviation of corrected counts.

Returns None

Attributes

- **gamma** (*float*) - A float denoting the negative slope of the distance-dependence regression line.
- **sigma** (*float*) - A float denoting the standard deviation of nonzero data about the distance-dependence regression line.
- **region_means** (*ndarray*) - A numpy array of type float32 and length equal to the number of regions. This is initialized to zeros until fragment correction values are found.

find_express_fragment_corrections(*mindistance=0*, *maxdistance=0*, *iterations=1000*, *remove_distance=False*, *usereads='cis'*, *regions=[]*, *precorrect=False*, *logged=True*, *kr=False*)

Using iterative approximation, learn correction values for each valid fragment.

Parameters

- **mindistance** (*int.*) – The minimum inter-fragment distance to be included in modeling.
- **maxdistance** (*int.*) – The maximum inter-fragment distance to be included in modeling.
- **iterations** (*int.*) – The number of iterations to use for learning fragment corrections.
- **remove_distance** (*bool.*) – Specifies whether the estimated distance-dependent portion of the signal is removed prior to learning fragment corrections.
- **usereads** (*str.*) – Specifies which set of interactions to use, 'cis', 'trans', or 'all'.
- **regions** (*list*) – A list of regions to calculate corrections for. If set as None, all region corrections are found.
- **precorrect** (*bool.*) – Use binning-based corrections in expected value calculations, resulting in a chained normalization approach.
- **logged** (*bool.*) – Use log-counts instead of counts for learning.
- **kr** (*bool.*) – Use the Knight Ruiz matrix balancing algorithm instead of weighted matrix balancing. This option ignores 'iterations' and 'logged'.

Returns None

Calling this function creates the following attributes:

Attributes

- **corrections** (*ndarray*) - A numpy array of type float32 and length equal to the number of fragments. All invalid fragments have an associated correction value of zero.

The 'normalization' attribute is updated to 'express' or 'binning-express', depending on if the 'precorrect' option is selected. In addition, if the 'remove_distance' option is selected, the 'region_means' attribute is updated such that the mean correction (sum of all valid regional correction value pairs) is adjusted to zero and the corresponding region mean is adjusted the same amount but the opposite sign.

find_probability_fragment_corrections(*mindistance=0*, *maxdistance=0*, *max_iterations=1000*, *minchange=0.0005*, *learningstep=0.1*, *precalculate=True*, *regions=[]*, *precorrect=False*)

Using gradient descent, learn correction values for each valid fragment based on a Log-Normal distribution of observations.

Parameters

- **mindistance** (*int.*) – The minimum inter-fragment distance to be included in modeling.
- **maxdistance** (*int.*) – The maximum inter-fragment distance to be included in modeling.
- **max_iterations** (*int.*) – The maximum number of iterations to carry on gradient descent for.
- **minchange** (*float*) – The cutoff threshold for early learning termination for the maximum absolute gradient value.
- **learningstep** (*float*) – The scaling factor for decreasing learning rate by if step doesn't meet armijo criterion.
- **precalculate** (*bool.*) – Specifies whether the correction values should be initialized at the fragment means.

- **regions** (*list*) – A list of regions to calculate corrections for. If set as None, all region corrections are found.
- **precorrect** (*bool.*) – Use binning-based corrections in expected value calculations, resulting in a chained normalization approach.

Returns None

Attributes

- **corrections** (*ndarray*) - A numpy array of type float32 and length equal to the number of fragments. All invalid fragments have an associated correction value of zero.

The 'normalization' attribute is updated to 'probability' or 'binning-probability', depending on if the 'precorrect' option is selected. In addition, the 'region_means' attribute is updated such that the mean correction (sum of all valid regional correction value pairs) is adjusted to zero and the corresponding region mean is adjusted the same amount but the opposite sign.

find_trans_mean()

Calculate the mean signal across all valid fragment-pair trans (inter-region) interactions.

Returns None

Attributes

- **trans_mean** (*float*) - A float corresponding to the mean signal of inter-region interactions.

load()

Load analysis parameters from h5dict specified at object creation and open h5dicts for associated `FiveCData` and `Fragment` objects.

Any call of this function will overwrite current object data with values from the last `save()` call.

Returns None

load_data(*filename*)

Load fragment-pair counts and fragment object from `FiveCData` object.

Parameters **filename** (*str.*) – Specifies the file name of the `FiveCData` object to associate with this analysis.

Returns None

Attributes

- **datafilename** (*str.*) - A string containing the relative path of the FiveCData file.
- **fragfilename** (*str.*) - A string containing the relative path of the Fragment file associated with the FiveCData file.
- **frags** (*filestream*) - A filestream to the hdf5 Fragment file such that all saved Fragment attributes can be accessed through this class attribute.
- **data** (*filestream*) - A filestream to the hdf5 FiveCData file such that all saved FiveCData attributes can be accessed through this class attribute.
- **chr2int** (*dict.*) - A dictionary that converts chromosome names to chromosome indices.
- **filter** (*ndarray*) - A numpy array of type int32 and size N where N is the number of fragments. This contains the inclusion status of each fragment with a one indicating included and zero indicating excluded and is initialized with all fragments included.

When a FiveCData object is associated with the project file, the 'history' attribute is updated with the history of the FiveCData object.

save (*out_fname=None*)

Save analysis parameters to h5dict.

Parameters **filename** (*str.*) – Specifies the file name of the FiveC object to save this analysis to.

Returns None

trans_heatmap (*region1*, *region2*, *binsize=1000000*, *binbounds1=None*, *start1=None*, *stop1=None*, *startfrag1=None*, *stopfrag1=None*, *binbounds2=None*, *start2=None*, *stop2=None*, *startfrag2=None*, *stopfrag2=None*, *datatype='enrichment'*, *arraytype='full'*, *returnmapping=False*, *dynamically_binned=False*, *minobservations=0*, *searchdistance=0*, *expansion_binsize=0*, *removefailed=False*, *skipfiltered=False*, *image_file=None*, ***kwargs*)

Return a heatmap of trans data of the type and shape specified by the passed arguments.

This function returns a heatmap for trans interactions between two regions, bounded by either 'start1', 'stop1', 'start2' and 'stop2' or 'startfrag1', 'stopfrag1', 'startfrag2', and 'stopfrag2' ('start' and 'stop' take precedence). The data in the array is determined by the 'datatype', being raw, fragment-corrected, distance-corrected, enrichment, or expected data. The array shape is always rectangular but can be either compact (which returns two arrays) or full. See fivec_binning for further explanation of 'datatype' and 'arraytype'. If using dynamic binning ('dynamically_binned' is set to True), 'minobservations', 'searchdistance', 'expansion_binsize', and 'removefailed' are used to control the dynamic binning process. Otherwise these arguments are ignored.

Parameters

- **region1** (*int.*) – The index of the first region to obtain data from.
- **region2** (*int.*) – The index of the second region to obtain data from.
- **binsize** (*int.*) – This is the coordinate width of each bin.
- **binbounds1** (*numpy array*) – An array containing start and stop coordinates for a set of user-defined bins for 'region1'. Any fragment not falling in a bin is ignored.
- **start1** (*int.*) – The coordinate at the beginning of the smallest bin from 'region1'. If unspecified, 'start1' will be the first multiple of 'binsize' below the 'startfrag1' mid. If there is a conflict between 'start1' and 'startfrag1', 'start1' is given preference. Optional.
- **stop1** (*int.*) – The largest coordinate to include in the array from 'region1', measured from fragment midpoints. If both 'stop1' and 'stopfrag1' are given, 'stop1' will override 'stopfrag1'. 'stop1' will be shifted higher as needed to make the last bin of size 'binsize'. Optional.
- **startfrag1** (*int.*) – The first fragment from 'region1' to include in the array. If unspecified and 'start1' is not given, this is set to the first valid fend in 'region1'. In cases where 'start1' is specified and conflicts with 'startfrag1', 'start1' is given preference. Optional.
- **stopfrag1** (*int.*) – The first fragment not to include in the array from 'region1'. If unspecified and 'stop1' is not given, this is set to the last valid fragment in 'region1' + 1. In cases where 'stop1' is specified and conflicts with 'stopfrag1', 'stop1' is given preference. Optional.
- **start1** – The coordinate at the beginning of the smallest bin from 'region1'. If unspecified, 'start1' will be the first multiple of 'binsize' below the 'startfrag1' mid. If there is a conflict between 'start1' and 'startfrag1', 'start1' is given preference. Optional.
- **binbounds2** (*numpy array*) – An array containing start and stop coordinates for a set of user-defined bins for 'region2'. Any fragment not falling in a bin is ignored.

- **stop2** (*int.*) – The largest coordinate to include in the array from 'region2', measured from fragment midpoints. If both 'stop2' and 'stopfrag2' are given, 'stop2' will override 'stopfrag2'. 'stop2' will be shifted higher as needed to make the last bin of size 'binsize'. Optional.
- **startfrag2** (*int.*) – The first fragment from 'region2' to include in the array. If unspecified and 'start2' is not given, this is set to the first valid fend in 'region2'. In cases where 'start2' is specified and conflicts with 'startfrag2', 'start2' is given preference. Optional.
- **stopfrag2** (*int.*) – The first fragment not to include in the array from 'region2'. If unspecified and 'stop2' is not given, this is set to the last valid fragment in 'region2' + 2. In cases where 'stop2' is specified and conflicts with 'stopfrag2', 'stop2' is given preference. Optional.
- **datatype** (*str.*) – This specifies the type of data that is processed and returned. Options are 'raw', 'distance', 'fragment', 'enrichment', and 'expected'. Observed values are aways in the first index along the last axis, except when 'datatype' is 'expected'. In this case, filter values replace counts. Conversely, if 'raw' is specified, non-filtered bins return value of 1. Expected values are returned for 'distance', 'fragment', 'enrichment', and 'expected' values of 'datatype'. 'distance' uses only the expected signal given distance for calculating the expected values, 'fragment' uses only fragment correction values, and both 'enrichment' and 'expected' use both correction and distance mean values.
- **arraytype** (*str.*) – This determines what shape of array data are returned in. Acceptable values are 'compact' (if unbinned) and 'full'. 'compact' means data are arranged in a N x M x 2 array where N and M are the number of forward and reverse probe fragments, respectively. If compact is selected, only data for the forward primers of 'region1' and reverse primers of 'region2' are returned. 'full' returns a square, symmetric array of size N x N x 2 where N is the total number of fragments or bins.
- **returnmapping** (*bool.*) – If 'True', a list containing the data array and mapping information is returned. Otherwise only a data array(s) is returned.
- **dynamically_binned** (*bool.*) – If 'True', return dynamically binned data.
- **minobservations** (*int.*) – The fewest number of observed reads needed for a bin to counted as valid and stop expanding.
- **searchdistance** (*int.*) – The furthest distance from the bin minpoint to expand bounds. If this is set to zero, there is no limit on expansion distance.
- **expansion_binsize** (*int.*) – The size of bins to use for data to pull from when expanding dynamic bins. If set to zero, unbinned data is used.
- **removefailed** (*bool.*) – If a non-zero 'searchdistance' is given, it is possible for a bin not to meet the 'minobservations' criteria before stopping looking. If this occurs and 'removefailed' is True, the observed and expected values for that bin are zero.
- **skipfiltered** (*bool.*) – If 'True', all interaction bins for filtered out fragments are removed and a reduced-size array is returned.
- **image_file** (*str.*) – If a filename is specified, a PNG image file is written containing the heatmap data. Arguments for the appearance of the image can be passed as additional keyword arguments.

Returns Array in format requested with 'arraytype' containing inter-region data requested with 'datatype'. If 'returnmapping' is True, a list is returned with mapping information. If 'arraytype' is 'full', a single data array and two 1d arrays of fragments corresponding to rows and columns, respectively is returned. If 'arraytype' is 'compact', two data arrays are returned

(forward1 by reverse2 and forward2 by reverse1) along with forward and reverse fragment positions for each array for a total of 5 arrays.

write_heatmap (*filename*, *binsize*, *includetrans=True*, *datatype='enrichment'*, *arraytype='full'*, *regions=[]*, *dynamically_binned=False*, *minobservations=0*, *searchdistance=0*, *expansion_binsize=0*, *removefailed=False*)

Create an h5dict file containing binned interaction arrays, bin positions, and an index of included regions.

Parameters

- **filename** (*str.*) – Location to write h5dict object to.
- **binsize** (*int.*) – Size of bins for interaction arrays. If "binsize" is zero, fragment interactions are returned without binning.
- **includetrans** (*bool.*) – Indicates whether trans interaction arrays should be calculated and saved.
- **datatype** (*str.*) – This specifies the type of data that is processed and returned. Options are 'raw', 'distance', 'fragment', 'enrichment', and 'expected'. Observed values are aways in the first index along the last axis, except when 'datatype' is 'expected'. In this case, filter values replace counts. Conversely, if 'raw' is specified, non-filtered bins return value of 1. Expected values are returned for 'distance', 'fragment', 'enrichment', and 'expected' values of 'datatype'. 'distance' uses only the expected signal given distance for calculating the expected values, 'fragment' uses only fragment correction values, and both 'enrichment' and 'expected' use both correction and distance mean values.
- **arraytype** (*str.*) – This determines what shape of array data are returned in. Acceptable values are 'compact' and 'full'. 'compact' means data are arranged in a N x M x 2 array where N is the number of bins, M is the maximum number of steps between included bin pairs, and data are stored such that bin n,m contains the interaction values between n and n + m + 1. 'full' returns a square, symmetric array of size N x N x 2.
- **regions** (*list.*) – If given, indicates which regions should be included. If left empty, all regions are included.
- **dynamically_binned** (*bool.*) – If 'True', return dynamically binned data.
- **minobservations** (*int.*) – The fewest number of observed reads needed for a bin to counted as valid and stop expanding.
- **searchdistance** (*int.*) – The furthest distance from the bin minpoint to expand bounds. If this is set to zero, there is no limit on expansion distance.
- **expansion_binsize** (*int.*) – The size of bins to use for data to pull from when expanding dynamic bins. If set to zero, unbinned data is used.
- **removefailed** – If a non-zero 'searchdistance' is given, it is possible for a bin not to meet the 'minobservations' criteria before stopping looking. If this occurs and 'removefailed' is True, the observed and expected values for that bin are zero.

Returns None

The following attributes are created within the hdf5 dictionary file. Arrays are accessible as datasets while the resolution is held as an attribute.

Attributes

- **resolution** (*int.*) - The bin size that data are accumulated in.
- **regions** (*ndarray*) - A numpy array containing region data for each region included in the heatmaps.

- **N.positions** (*ndarray*) - A series of numpy arrays of type int32, one for each region where N is the region index, containing one row for each bin and four columns denoting the start and stop coordinates and first fragment and last fragment plus one for each bin. This is included if data is in the 'full' format.
- **N.forward_positions** (*ndarray*) - A series of numpy arrays of type int32, one for each region where N is the region index, containing one row for each bin along the first axis and four columns denoting the start and stop coordinates and first fragment and last fragment plus one for each bin. This is included if data is in the 'compact' format and corresponds to only forward strand fragments.
- **N.reverse_positions** (*ndarray*) - A series of numpy arrays of type int32, one for each region where N is the region index, containing one row for each bin along the second axis and four columns denoting the start and stop coordinates and first fragment and last fragment plus one for each bin. This is included if data is in the 'compact' format and corresponds to only reverse strand fragments.
- **N.counts** (*ndarray*) - A series of numpy arrays of type int32, one for each region where N is the region index, containing the observed counts for valid fragment combinations. If arrays are in the 'compact' format, the first axis corresponds to forward fragments and the second axis corresponds to reverse fragments. If the array is in the 'upper' format,data are in an upper-triangle format such that they have N * (N - 1) / 2 entries where N is the number of fragments or bins in the region.
- **N.expected** (*ndarray*) - A series of numpy arrays of type float32, one for each region where N is the region index, containing the expected counts for valid fragment combinations. If the array is in the 'upper' format,data are in an upper-triangle format such that they have N * (N - 1) / 2 entries where N is the number of fragments or bins in the region.
- **N_by_M.counts** (*ndarray*) - A series of numpy arrays of type int32, one for each region pair N and M if trans data are included, containing the observed counts for valid fragment combinations. The region index order specifies which axis corresponds to which region. If data are in the 'compact' format, both region index orders will be present.
- **N_by_M.expected** (*ndarray*) - A series of numpy arrays of type float32, one for each region pair N and M if trans data are included, containing the expected counts for valid fend combinations. The chromosome name order specifies which axis corresponds to which region. If data are in the 'compact' format, both region index orders will be present.

13.6 The HiC class

A class for handling HiC analysis.

class `hifive.hic.`**`HiC`** (*filename*, *mode='r'*, *silent=False*)

This is the class for handling HiC analysis.

This class relies on `Fend` and `HiCData` for genomic position and interaction count data. Use this class to perform filtering of fends based on coverage, model fend bias and distance dependence, and downstream analysis and manipulation. This includes binning of data, plotting of data, modeling of data, and statistical analysis.

Note: This class is also available as hifive.HiC

When initialized, this class creates an h5dict in which to store all data associated with this object.

Parameters

- **`filename`** (*str.*) – The file name of the h5dict. This should end with the suffix '.hdf5'

- `mode` (*str.*) – The mode to open the h5dict with. This should be 'w' for creating or overwriting an h5dict with name given in filename.
- `silent` (*bool.*) – Indicates whether to print information about function execution for this object.

Returns `HiC` class object.

Attributes

- **file** (*str.*) - A string containing the name of the file passed during object creation for saving the object to.
- **silent** (*bool.*) - A boolean indicating whether to suppress all of the output messages.
- **history** (*str.*) - A string containing all of the commands executed on this object and their outcome.
- **normalization** (*str.*) - A string stating which type of normalization has been performed on this object. This starts with the value 'none'.
- **comm** (*class*) - A link to the MPI.COMM_WORLD class from the mpi4py package. If this package isn't present, this is set to 'None'.
- **rank** (*int.*) - The rank integer of this process, if running with mpi, otherwise set to zero.
- **num_procs** (*int.*) - The number of processes being executed in parallel. If mpi4py package is not present, this is set to one.

In addition, many other attributes are initialized to the 'None' state.

cis_heatmap(*chrom*, *start=None*, *stop=None*, *startfend=None*, *stopfend=None*, *binsize=0*, *binbounds=None*, *datatype='enrichment'*, *arraytype='compact'*, *maxdistance=0*, *skipfiltered=False*, *returnmapping=False*, *dynamically_binned=False*, *minobservations=0*, *searchdistance=0*, *expansion_binsize=0*, *removefailed=False*, *image_file=None*, *proportional=False*, ***kwargs*)

Return a heatmap of cis data of the type and shape specified by the passed arguments.

This function returns a heatmap for a single chromosome region, bounded by either 'start' and 'stop' or 'startfend' and 'stopfend' ('start' and 'stop' take precedence), or if given, the outer coordinates of the array passed by 'binbounds'. If none of these are specified, data for the complete chromosome is used. The data in the array is determined by the 'datatype', being raw, fend-corrected, distance-corrected, enrichment, or expected data. The array shape is given by 'arraytype' and can be compact, upper, or full. See `hic_binning` for further explanation of 'datatype' and 'arraytype'. The returned data will include interactions ranging from zero to 'maxdistance' apart. If maxdistance is zero, all interactions within the requested bounds are returned. If using dynamic binning ('dynamically_binned' is set to True), 'minobservations', 'searchdistance', 'expansion_binsize', and 'removefailed' are used to control the dynamic binning process. Otherwise these arguments are ignored.

Parameters

- `chrom` (*str.*) – The name of a chromosome to obtain data from.
- `start` (*int.*) – The smallest coordinate to include in the array, measured from fend midpoints. If both 'start' and 'startfend' are given, 'start' will override 'startfend'. If unspecified, this will be set to the midpoint of the first fend for 'chrom'. Optional.
- `stop` (*int.*) – The largest coordinate to include in the array, measured from fend midpoints. If both 'stop' and 'stopfend' are given, 'stop' will override 'stopfend'. If unspecified, this will be set to the midpoint of the last fend plus one for 'chrom'. Optional.

- **startfend** (*int.*) – The first fend to include in the array. If unspecified and 'start' is not given, this is set to the first fend in 'chrom'. In cases where 'start' is specified and conflicts with 'startfend', 'start' is given preference. Optional
- **stopfend** (*str.*) – The first fend not to include in the array. If unspecified and 'stop' is not given, this is set to the last fend in 'chrom' plus one. In cases where 'stop' is specified and conflicts with 'stopfend', 'stop' is given preference. Optional.
- **binsize** (*int.*) – This is the coordinate width of each bin. If 'binsize' is zero, unbinned data is returned. If 'binbounds' is not None, this value is ignored.
- **binbounds** (*numpy array*) – An array containing start and stop coordinates for a set of user-defined bins. Any fend not falling in a bin is ignored. Optional.
- **datatype** (*str.*) – This specifies the type of data that is processed and returned. Options are 'raw', 'distance', 'fend', 'enrichment', and 'expected'. Observed values are always in the first index along the last axis, except when 'datatype' is 'expected'. In this case, filter values replace counts. Conversely, if 'raw' is specified, unfiltered fends return value of one. Expected values are returned for 'distance', 'fend', 'enrichment', and 'expected' values of 'datatype'. 'distance' uses only the expected signal given distance for calculating the expected values, 'fend' uses only fend correction values, and both 'enrichment' and 'expected' use both correction and distance mean values.
- **arraytype** (*str.*) – This determines what shape of array data are returned in. Acceptable values are 'compact', 'full', and 'upper'. 'compact' means data are arranged in a N x M x 2 array where N is the number of fends or bins, M is the maximum number of steps between included fend pairs or bin pairs and data are stored such that bin n,m contains the interaction values between n and n + m + 1. 'full' returns a square, symmetric array of size N x N x 2. 'upper' returns only the flattened upper triangle of a full array, excluding the diagonal of size (N * (N - 1) / 2) x 2.
- **maxdistance** (*str.*) – This specifies the maximum coordinate distance between bins that will be included in the array. If set to zero, all distances are included.
- **skipfiltered** (*bool.*) – If 'True', all interaction bins for filtered out fends are removed and a reduced-size array is returned.
- **returnmapping** (*bool.*) – If 'True', a list containing the data array and a 1d array containing fend numbers included in the data array if unbinned or a 2d array of N x 4 containing the first fend and last fend plus one included in each bin and first and last coordinates if binned is return. Otherwise only the data array is returned.
- **dynamically_binned** (*bool.*) – If 'True', return dynamically binned data.
- **minobservations** (*int.*) – The fewest number of observed reads needed for a bin to counted as valid and stop expanding.
- **searchdistance** (*int.*) – The furthest distance from the bin minpoint to expand bounds. If this is set to zero, there is no limit on expansion distance.
- **expansion_binsize** (*int.*) – The size of bins to use for data to pull from when expanding dynamic bins. If set to zero, unbinned data is used.
- **removefailed** (*bool.*) – If a non-zero 'searchdistance' is given, it is possible for a bin not to meet the 'minobservations' criteria before stopping looking. If this occurs and 'removefailed' is True, the observed and expected values for that bin are zero.
- **image_file** (*str.*) – If a filename is specified, a PNG image file is written containing the heatmap data. Arguments for the appearance of the image can be passed as additional keyword arguments.

- **proportional** (*bool.*) – Indicates whether interactions should proportionally contribute to bins based on the amount of overlap instead of being attributed solely based on midpoint. Only valid for binned heatmaps and does not work in conjunction with dynamic binning.

Returns Array in format requested with 'arraytype' containing data requested with 'datatype'. If returnmapping is True, a list is returned containined the requested data array and an array of associated positions (dependent on the binning options selected).

filter_fends (*mininteractions=10*, *mindistance=0*, *maxdistance=0*)

Iterate over the dataset and remove fends that do not have 'minobservations' within 'maxdistance' of themselves using only unfiltered fends.

In order to create a set of fends that all have the necessary number of interactions, after each round of filtering, fend interactions are retallied using only interactions that have unfiltered fends at both ends.

Parameters

- **mininteractions** (*int.*) – The required number of interactions for keeping a fend in analysis.
- **mindistance** (*int.*) – The minimum inter-fend distance used to count fend interactions.
- **maxdistance** (*int.*) – The maximum inter-fend distance used to count fend interactions. A value of 0 indicates no maximum should be used.

Returns None

find_binning_fend_corrections (*mindistance=0, maxdistance=0, chroms=[], num_bins=[20, 20, 20], parameters=['even', 'even', 'even-const'], model=['gc', 'len', 'distance'], learning_threshold=1.0, max_iterations=10, usereads='cis', pseudocounts=0*)

Using a multivariate binning model, learn correction values for combinations of model parameter bins. This function is MPI compatible.

Parameters

- **mindistance** (*int.*) – The minimum inter-fend distance to be included in modeling.
- **maxdistance** (*int.*) – The maximum inter-fend distance to be included in modeling.
- **chroms** (*list*) – A list of chromosomes to calculate corrections for. If set as None, all chromosome corrections are found.
- **remove_distance** (*bool.*) – Use distance dependence curve in prior probability calculation for each observation.
- **model** (*list*) – A list of fend features to be used in model. Valid values are 'len', 'distance', and any features included in the creation of the associated Fend object. The 'distance' parameter is only good with 'cis' or 'all' reads. If used with 'all', distances will be partitioned into n - 1 bins and the final distance bin will contain all trans data.
- **num_bins** (*list*) – A list of the number of approximately equal-sized bins two divide model components into.
- **parameters** (*list*) – A list of types, one for each model parameter. Types can be either 'even' or 'fixed', indicating whether each parameter bin should contain approximately even numbers of interactions or be of fixed width spanning 1 / Nth of the range of the parameter's values, respectively. Parameter types can also have the suffix '-const' to indicate that the parameter should not be optimized.
- **learning_threshold** (*float*) – The minimum change in log-likelihood needed to continue iterative learning process.

- **max_iterations** (*int.*) – The maximum number of iterations to use for learning model parameters.
- **usereads** (*str.*) – Specifies which set of interactions to use, 'cis', 'trans', and 'all'.
- **pseudocounts** (*int.*) – The number of pseudo-counts to add to each bin prior to seeding and learning normalization values.

Returns None

Attributes

- **model_parameters** (*ndarray*) - A numpy array of strings containing model parameter names. If distance was included in the 'model' option, it is not included in this array since it is only for learning values, not for subsequent corretion.
- **binning_num_bins** (*ndarray*) - A numpy array of type int32 containing the number of bins for each non-distance model parameter.
- **binning corrections** (*ndarray*) - A numpy array of type float32 and length equal to the sum of binning_num_bins * (binning_num_bins - 1) / 2. This array contains a 1D stack of correction values, ordered according to the parameter order in the 'model_parameters' attribute.
- **binning_correction_indices** (*ndarray*) - A numpy array of type int32 and length equal to the number of non-distance model parameters plus one. This array contains the first position in 'binning_corrections' for the first bin of the model parameter in the corresponding position in the 'model_parameters' array. The last position in the array contains the total number of binning correction values.
- **binning_fend_indices** (*ndarray*) - A numpy array of type int32 and size N x M x 2 where M is the number of non-distance model parameters and N is the number of fends. This array contains the binning index for each parameter for each fend for the first and the second position in the correction array.

The 'normalization' attribute is updated to 'binning'.

find_distance_parameters (*numbins=90*, *minsize=200*, *maxsize=0*, *corrected=False*)

Count reads and possible interactions from valid fend pairs in each distance bin to find mean bin signals. This function is MPI compatible.

This partitions the range of interaction distances (measured from mipoints of the involved fends) from the 'minsize' to 'maxsize' into a number of partitions equal to 'numbins'. The first bin contains all distances less than or equal to 'minsize'. The remaining bins are defined such that their log ranges are equal to one another. The curve defined by the mean interaction value of each bin can be smoothed using a triangular smoothing operation.

Parameters

- **numbins** (*int.*) – The number of bins to divide the distance range into. The first bin extends from zero to 'minsize', while the remaining bins are divided into evenly-spaced log-sized bins from 'minsize' to 'maxsize' or the maximum inter-fend distance, whichever is greater.
- **minsize** (*int.*) – The upper size limit of the smallest distance bin.
- **maxsize** (*int.*) – If this value is larger than the largest included chromosome, it will extend bins out to maxsize. If this value is smaller, it is ignored.
- **corrected** (*bool.*) – If True, correction values are applied to counts prior to summing.

Returns None

Attributes

- **distance_parameters** (*ndarray*) - A numpy array of type float32 and size of N x 3 where N is one less than the number of distance bins containing at least one valid observation out of the 'numbins' number of bins that the distance range was divided into. The First column contains upper distance cutoff for each bin, the second column contains the slope associated with each bin line segment, and the third column contains the line segment intercepts. Line segments describe the relationship of observation counts versus distance.
- **bin_distance_parameters** (*ndarray*) - A numpy array of type float32 and size of N x 3 where N is one less than the number of distance bins containing at least one valid observation out of the 'numbins' number of bins that the distance range was divided into. The First column contains upper distance cutoff for each bin, the second column contains the slope associated with each bin line segment, and the third column contains the line segment intercepts. Line segments describe the relationship of binary observations versus distance.
- **chromosome_means** (*ndarray*) - A numpy array of type float32 and length equal to the number of chromosomes. This is initialized to zeros until fend correction values are found.

find_express_fend_corrections(*iterations=100*, *mindistance=0*, *maxdistance=0*, *remove_distance=True*, *usereads='cis'*, *mininteractions=0*, *minchange=0.0001*, *chroms=[]*, *precorrect=False*, *binary=False*, *kr=False*)

Using iterative matrix-balancing approximation, learn correction values for each valid fend. This function is MPI compatible.

Parameters

- **iterations** (*int.*) – The minimum number of iterations to use for learning fend corrections.
- **mindistance** (*int.*) – This is the minimum distance between fend midpoints needed to be included in the analysis. All possible and observed interactions with a distance shorter than this are ignored. If 'usereads' is set to 'trans', this value is ignored.
- **maxdistance** (*int.*) – The maximum inter-fend distance to be included in modeling. If 'usereads' is set to 'trans', this value is ignored.
- **remove_distance** (*bool.*) – Specifies whether the estimated distance-dependent portion of the signal is removed prior to learning fend corrections.
- **usereads** (*str.*) – Specifies which set of interactions to use, 'cis', 'trans', or 'all'.
- **mininteractions** (*int.*) – If a non-zero 'mindistance' is specified or only 'trans' interactions are used, fend filtering will be performed again to ensure that the data being used is sufficient for analyzed fends. This parameter may specify how many interactions are needed for valid fends. If not given, the value used for the last call to `filter_fends()` is used or, barring that, one.
- **minchange** (*float*) – The minimum mean change in fend correction parameter values needed to keep running past 'iterations' number of iterations. If using the Knight-Ruiz algorithm this is the residual cutoff.
- **chroms** (*list*) – A list of chromosomes to calculate corrections for. If set as None, all chromosome corrections are found.
- **precorrect** (*bool.*) – Use binning-based corrections in expected value calculations, resulting in a chained normalization approach.
- **binary** (*bool.*) – Use binary indicator instead of counts.

- **kr** (*bool.*) – Use the Knight Ruiz matrix balancing algorithm instead of weighted matrix balancing. This option ignores 'iterations'.

Returns None

Attributes

- **corrections** (*ndarray*) - A numpy array of type float32 and length equal to the number of fends. All invalid fends have an associated correction value of zero.

The 'normalization' attribute is updated to 'express' or 'binning-express', depending on if the 'precorrect' option is selected. In addition, the 'chromosome_means' attribute is updated such that the mean correction (sum of all valid chromosomal correction value pairs) is adjusted to zero and the corresponding chromosome mean is adjusted the same amount but the opposite sign.

find_probability_fend_corrections(*mindistance=0*, *maxdistance=0*, *minchange=0.0001*, *max_iterations=1000*, *learningstep=0.5*, *chroms=[]*, *precalculate=True*, *precorrect=False*, *model='binomial'*)

Using gradient descent, learn correction values for each valid fend based on a binomial or Poisson distribution of observations. This function is MPI compatible.

Parameters

- **mindistance** (*int.*) – The minimum inter-fend distance to be included in modeling.
- **maxdistance** (*int.*) – The maximum inter-fend distance to be included in modeling.
- **minchange** (*float*) – The cutoff threshold for early learning termination for the maximum absolute gradient value.
- **max_iterations** (*int.*) – The maximum number of iterations to carry on gradient descent for.
- **learningstep** (*float*) – The scaling factor for decreasing learning rate by if step doesn't meet armijo criterion.
- **chroms** (*list*) – A list of chromosomes to calculate corrections for. If set as None, all chromosome corrections are found.
- **precalculate** (*bool.*) – Specifies whether the correction values should be initialized at the fend means.
- **precorrect** (*bool.*) – Use binning-based corrections in expected value calculations, resulting in a chained normalization approach.
- **model** (*str.*) – Which probability model to use, either 'poisson' or 'binomial'. If 'poisson' is chosen, read counts are used. If 'binomial' is chosen, reads are converted to a 0/1 indicator of observed/unobserved status.

Returns None

Attributes

- **corrections** (*ndarray*) - A numpy array of type float32 and length equal to the number of fends. All invalid fends have an associated correction value of zero.

The 'normalization' attribute is updated to 'probability' or 'binning-probability', depending on if the 'precorrect' option is selected. In addition, the 'chromosome_means' attribute is updated such that the mean correction (sum of all valid chromosomal correction value pairs) is adjusted to zero and the corresponding chromosome mean is adjusted the same amount but the opposite sign.

find_trans_means()

Calculate the mean signals across all valid fend-pair trans interactions for each chromosome pair.

Returns None

Attributes

- **trans_mean** (*float*) - A float corresponding to the mean signal of inter-chromosome interactions.

learn_fend_3D_model(*chrom*, *minobservations=10*)

Learn coordinates for a 3D model of data using an approximate PCA dimensional reduction.

This function makes use of the `mlpy` function `PCAFast()` to reduce the data to a set of three coordinates per fend. Cis data for all unfiltered fends for the specified chromosome are dynamically binned to yield a complete distance matrix. The diagonal is set equal to the highest valid enrichment value after dynamic binning. This N x N matrix is passed to `PCAFast()` and reduced to an N x 3 matrix.

Parameters

- **chrom** (*str.*) – The chromosome to learn the model for.
- **minobservations** (*int.*) – The minimum number of observed reads needed to cease bin expansion in the dynamic binning phase.

Returns Array containing a row for each valid fend and columns containing X coordinate, Y coordinate, Z coordinate, and sequence coordinate (fend midpoint).

load()

Load analysis parameters from h5dict specified at object creation and open h5dicts for associated `HiCData` and `Fend` objects.

Any call of this function will overwrite current object data with values from the last `save()` call.

Returns None

load_data(*filename*)

Load fend-pair counts and fend object from `HiCData` object.

Parameters **filename** (*str.*) – Specifies the file name of the `HiCData` object to associate with this analysis.

Returns None

Attributes

- **datafilename** (*str.*) - A string containing the relative path of the HiCData file.
- **fendfilename** (*str.*) - A string containing the relative path of the Fend file associated with the HiCData file.
- **fends** (*filestream*) - A filestream to the hdf5 Fragment file such that all saved Fend attributes can be accessed through this class attribute.
- **data** (*filestream*) - A filestream to the hdf5 FiveCData file such that all saved HiCData attributes can be accessed through this class attribute.
- **chr2int** (*dict.*) - A dictionary that converts chromosome names to chromosome indices.
- **filter** (*ndarray*) - A numpy array of type int32 and size N where N is the number of fends. This contains the inclusion status of each fend with a one indicating included and zero indicating excluded and is initialized with all fends included.

When a HiCData object is associated with the project file, the 'history' attribute is updated with the history of the HiCData object.

reset_filter()

Return all fends to a valid filter state.

Returns None

save (*out_fname=None*)

Save analysis parameters to h5dict.

Parameters **filename** (*str.*) – Specifies the file name of the *HiC* object to save this analysis to.

Returns None

trans_heatmap (*chrom1*, *chrom2*, *start1=None*, *stop1=None*, *startfend1=None*, *stopfend1=None*, *binbounds1=None*, *start2=None*, *stop2=None*, *startfend2=None*, *stopfend2=None*, *binbounds2=None*, *binsize=1000000*, *skipfiltered=False*, *datatype='enrichment'*, *returnmapping=False*, *dynamically_binned=False*, *minobservations=0*, *searchdistance=0*, *expansion_binsize=0*, *removefailed=False*, *image_file=None*, ***kwargs*)

Return a heatmap of trans data of the type and shape specified by the passed arguments.

This function returns a heatmap for trans interactions between two chromosomes within a region, bounded by either 'start1', 'stop1', 'start2' and 'stop2' or 'startfend1', 'stopfend1', 'startfend2', and 'stopfend2' ('start' and 'stop' take precedence), or if given, the outer coordinates of the arrays passed by 'binbounds1' and 'binbounds2'. The data in the array is determined by the 'datatype', being raw, fend-corrected, distance-corrected, enrichment, or expected data. The array shape is always rectangular. See *hic_binning* for further explanation of 'datatype'. If using dynamic binning ('dynamically_binned' is set to True), 'minobservations', 'searchdistance', 'expansion_binsize', and 'removefailed' are used to control the dynamic binning process. Otherwise these arguments are ignored.

Parameters

- **chrom1** (*str.*) – The name of the first chromosome to obtain data from.
- **chrom2** (*str.*) – The name of the second chromosome to obtain data from.
- **start1** (*int.*) – The coordinate at the beginning of the smallest bin from 'chrom1'. If unspecified, 'start1' will be the first multiple of 'binsize' below the 'startfend1' mid. If there is a conflict between 'start1' and 'startfend1', 'start1' is given preference. Optional.
- **stop1** (*int.*) – The largest coordinate to include in the array from 'chrom1', measured from fend midpoints. If both 'stop1' and 'stopfend1' are given, 'stop1' will override 'stopfend1'. 'stop1' will be shifted higher as needed to make the last bin of size 'binsize'. Optional.
- **startfend1** (*int.*) – The first fend from 'chrom1' to include in the array. If unspecified and 'start1' is not given, this is set to the first valid fend in 'chrom1'. In cases where 'start1' is specified and conflicts with 'startfend1', 'start1' is given preference. Optional
- **stopfend1** – The first fend not to include in the array from 'chrom1'. If unspecified and 'stop1' is not given, this is set to the last valid fend in 'chrom1' + 1. In cases where 'stop1' is specified and conflicts with 'stopfend1', 'stop1' is given preference. Optional.
- **binbounds1** (*numpy array*) – An array containing start and stop coordinates for a set of user-defined bins to use for partitioning 'chrom1'. Any fend not falling in a bin is ignored.
- **start2** (*int.*) – The coordinate at the beginning of the smallest bin from 'chrom2'. If unspecified, 'start2' will be the first multiple of 'binsize' below the 'startfend2' mid. If there is a conflict between 'start2' and 'startfend2', 'start2' is given preference. Optional.
- **stop2** (*int.*) – The largest coordinate to include in the array from 'chrom2', measured from fend midpoints. If both 'stop2' and 'stopfend2' are given, 'stop2' will override 'stopfend2'. 'stop2' will be shifted higher as needed to make the last bin of size 'binsize'. Optional.

- **startfend2** (*int.*) – The first fend from 'chrom2' to include in the array. If unspecified and 'start2' is not given, this is set to the first valid fend in 'chrom2'. In cases where 'start2' is specified and conflicts with 'startfend2', 'start2' is given preference. Optional
- **stopfend2** (*str.*) – The first fend not to include in the array from 'chrom2'. If unspecified and 'stop2' is not given, this is set to the last valid fend in 'chrom2' + 1. In cases where 'stop2' is specified and conflicts with 'stopfend2', 'stop1' is given preference. Optional.
- **binbounds2** (*numpy array*) – An array containing start and stop coordinates for a set of user-defined bins to use for partitioning 'chrom2'. Any fend not falling in a bin is ignored.
- **binsize** (*int.*) – This is the coordinate width of each bin. If binbounds is not None, this value is ignored.
- **skipfiltered** (*bool.*) – If 'True', all interaction bins for filtered out fends are removed and a reduced-size array is returned.
- **datatype** (*str.*) – This specifies the type of data that is processed and returned. Options are 'raw', 'distance', 'fend', 'enrichment', and 'expected'. Observed values are always in the first index along the last axis, except when 'datatype' is 'expected'. In this case, filter values replace counts. Conversely, if 'raw' is specified, unfiltered fends return value of one. Expected values are returned for 'distance', 'fend', 'enrichment', and 'expected' values of 'datatype'. 'distance' uses only the expected signal given distance for calculating the expected values, 'fend' uses only fend correction values, and both 'enrichment' and 'expected' use both correction and distance mean values.
- **returnmapping** (*bool.*) – If 'True', a list containing the data array and two 2d arrays of N x 4 containing the first fend and last fend plus one included in each bin and first and last coordinates for the first and second chromosomes is returned. Otherwise only the data array is returned.
- **dynamically_binned** (*bool.*) – If 'True', return dynamically binned data.
- **minobservations** (*int.*) – The fewest number of observed reads needed for a bin to counted as valid and stop expanding.
- **searchdistance** (*int.*) – The furthest distance from the bin minpoint to expand bounds. If this is set to zero, there is no limit on expansion distance.
- **expansion_binsize** (*int.*) – The size of bins to use for data to pull from when expanding dynamic bins. If set to zero, unbinned data is used.
- **removefailed** (*bool.*) – If a non-zero 'searchdistance' is given, it is possible for a bin not to meet the 'minobservations' criteria before stopping looking. If this occurs and 'removefailed' is True, the observed and expected values for that bin are zero.
- **image_file** (*str.*) – If a filename is specified, a PNG image file is written containing the heatmap data. Arguments for the appearance of the image can be passed as additional keyword arguments.

Returns Array in format requested with 'arraytype' containing data requested with 'datatype'. If returnmapping is True, a list is returned containined the requested data array and an array of associated positions (dependent on the binning options selected).

write_heatmap (*filename*, *binsize*, *includetrans=True*, *datatype='enrichment'*, *chroms=[]*, *dynamically_binned=False*, *minobservations=0*, *searchdistance=0*, *expansion_binsize=0*, *removefailed=False*)

Create an h5dict file containing binned interaction arrays, bin positions, and an index of included chromosomes. This function is MPI compatible.

Parameters

- **filename** (*str.*) – Location to write h5dict object to.
- **binsize** (*int.*) – Size of bins for interaction arrays.
- **includetrans** (*bool.*) – Indicates whether trans interaction arrays should be calculated and saved.
- **datatype** (*str.*) – This specifies the type of data that is processed and returned. Options are 'raw', 'distance', 'fend', 'enrichment', and 'expected'. Observed values are always in the first index along the last axis, except when 'datatype' is 'expected'. In this case, filter values replace counts. Conversely, if 'raw' is specified, unfiltered fends return value of one. Expected values are returned for 'distance', 'fend', 'enrichment', and 'expected' values of 'datatype'. 'distance' uses only the expected signal given distance for calculating the expected values, 'fend' uses only fend correction values, and both 'enrichment' and 'expected' use both correction and distance mean values.
- **chroms** (*list*) – A list of chromosome names indicating which chromosomes should be included. If left empty, all chromosomes are included. Optional.
- **dynamically_binned** (*bool.*) – If 'True', return dynamically binned data.
- **minobservations** (*int.*) – The fewest number of observed reads needed for a bin to counted as valid and stop expanding.
- **searchdistance** (*int.*) – The furthest distance from the bin minpoint to expand bounds. If this is set to zero, there is no limit on expansion distance.
- **expansion_binsize** (*int.*) – The size of bins to use for data to pull from when expanding dynamic bins. If set to zero, unbinned data is used.
- **removefailed** (*bool.*) – If a non-zero 'searchdistance' is given, it is possible for a bin not to meet the 'minobservations' criteria before stopping looking. If this occurs and 'removefailed' is True, the observed and expected values for that bin are zero.

Returns None

The following attributes are created within the hdf5 dictionary file. Arrays are accessible as datasets while the resolution is held as an attribute.

Attributes

- **resolution** (*int.*) - The bin size that data are accumulated in.
- **chromosomes** (*ndarray*) - A numpy array of strings listing all of the chromosomes included in the heatmaps.
- **N.positions** (*ndarray*) - A series of numpy arrays of type int32, one for each chromosome where N is the chromosome name, containing one row for each bin and four columns denoting the start and stop coordinates and first fend and last fend plus one for each bin.
- **N.counts** (*ndarray*) - A series of numpy arrays of type int32, one for each chromosome where N is the chromosome name, containing the observed counts for valid fend combinations. Arrays are in an upper-triangle format such that they have N * (N - 1) / 2 entries where N is the number of fends or bins in the chromosome.
- **N.expected** (*ndarray*) - A series of numpy arrays of type float32, one for each chromosome where N is the chromosome name, containing the expected counts for valid fend combinations. Arrays are in an upper-triangle format such that they have N * (N - 1) / 2 entries where N is the number of fends in the chromosome.
- **N_by_M.counts** (*ndarray*) - A series of numpy arrays of type int32, one for each chromosome pair N and M if trans data are included, containing the observed counts for valid

fend combinations. The chromosome name order specifies which axis corresponds to which chromosome.

- **N_by_M.expected** (*ndarray*) - A series of numpy arrays of type float32, one for each chromosome pair N and M if trans data are included, containing the expected counts for valid fend combinations. The chromosome name order specifies which axis corresponds to which chromosome.

write_multiresolution_heatmap (*filename*, *datatype='fend'*, *maxbinsize=1280000*, *minbinsize=5000*, *trans_maxbinsize=None*, *trans_minbinsize=None*, *minobservations=5*, *chroms=None*, *includetrans=True*, *midbinsize=40000*)

Create a multi-resolution heatmap file containing data for each requested chromosome. This function is MPI-compatible.

Parameters

- **filename** (*str.*) – Location to write the multi-resolution heamtap to.
- **datatype** (*str.*) – This specifies the type of data that is processed and returned. Options are 'raw', 'distance', 'fend', and 'enrichment'. Observed values are always in the first index along the last axis. If 'raw' is specified, unfiltered fends return value of one. Expected values are returned for 'distance', 'fend', 'enrichment', and 'expected' values of 'datatype'. 'distance' uses only the expected signal given distance for calculating the expected values, 'fend' uses only fend correction values, and 'enrichment' uses both correction and distance mean values.
- **maxbinsize** (*int.*) – The maximum sized bin (lowest resolution) heatmap to be produced for each chromosome.
- **minbinsize** (*int.*) – The minimum sized bin (highest resolution) heatmap to be produced for each chromosome. The maxbinsize and minbinsize must differ by an exponent of 2 (e.g. minbinsize * 2^N = maxbinsize for some integer N).
- **trans_maxbinsize** (*int.*) – The maximum sized bin (lowest resolution) heatmap to be produced for inter-chromosomal interactions. If trans_maxbinsize is None, the value maxbinsize is used for inter-chromosomal interactions.
- **trans_minbinsize** (*int.*) – The minimum sized bin (highest resolution) heatmap to be produced for inter-chromosomal interactions. If trans_minbinsize is None, the value minbinsize is used for inter-chromosomal interactions. The trans_maxbinsize and trans_minbinsize must differ by an exponent of 2 (e.g. trans_minbinsize * 2^N = trans_maxbinsize for some integer N).
- **minobservations** (*int.*) – The minimum number of reads needed for a bin to be considered valid and be included in the heatmap.
- **chroms** (*list*) – A list of chromosomes to include in the multi-resolution heatmap. If chroms is None, all chromosomes will be included.
- **includetrans** (*bool.*) – Indicates whether to calculate all of the inter-chromosomal interaction multi-resolution heatmaps.
- **midbinsize** (*int.*) – This is used to determine the smallest bin size (highest resolution) complete heatmap to generate in producing the multi-resolution heatmap. It does not affect the resulting output but can be used to limit the total memory usage, with higher values using less memory but more time.

The multi-resolution heatmap file has the following structure - header: magic number "42054205" - 4 bytes (8 2-bit hexadecimals) flag indicating if trans are included - 4 bytes (1 int32) number of chromosomes "N" - 4 bytes (1 int32) chromosome names - N * 10 bytes (N * 10 chars) chrom index bounds - (N * (N + 1)

/ 2 + 1) * 4 bytes (N * (N + 1) / 2 + 1 int32) intra-chrom top layer number of paritions - N * 4 bytes (N int32) inter-chrom top layer number of paritions - N * 4 bytes (N int32) chrom total data bins - (N * (N + 1) / 2) * 4 bytes (N int32) chrom total index bins - (N * (N + 1) / 2) * 4 bytes (N int32) intra-chrom start coordinates - N * 4 bytes (N int32) intra-chrom stop coordinates - N * 4 bytes (N int32) inter-chrom start coordinates - N * 4 bytes (N int32) inter-chrom stop coordinates - N * 4 bytes (N int32) chrom min scores - (N * (N + 1) / 2) * 4 bytes (N * (N + 1) / 2 float32) chrom max scores - (N * (N + 1) / 2) * 4 bytes (N * (N + 1) / 2 float32) intra-chromosome largest bin size - 4 bytes (1 int32) intra-chromosome smallest bin size - 4 bytes (1 int32) inter-chromosome largest bin size - 4 bytes (1 int32) inter-chromosome smallest bin size - 4 bytes (1 int32) minimum number of observed reads - 4 bytes (1 int32)

interaction and index arrays: data_array_1_by_1 float32 index_array_1_by_1 int32 shape_array_1_by_1 int32 data_array_1_by_2 float32 index_array_1_by_2 int32 shape_array_1_by_2 int32 ... data_array_1_by_N float32 index_array_1_by_N int32 shape_array_1_by_N int32 data_array_2_by_2 float32 index_array_2_by_2 int32 shape_array_2_by_2 int32 data_array_2_by_3 float32 index_array_2_by_3 int32 shape_array_2_by_3 int32 data_array_2_by_N float32 index_array_2_by_N int32 shape_array_2_by_N int32 ... data_array_N_by_N float32 index_array_N_by_N int32 shape_array_N_by_N int32

Each data array starts with a flattened array of the complete heatmap with the largest bin size. Intra-chromosomal heatmaps are upper-triangle arrays including the diagonal while inter-chromosomal arrays are rectangles. For each bin, there is a corresponding position in the index array pointing to the start index in the data array for the interactions contained within the data bin, partitioned into smaller bin sizes. Bins are always partitioned by a factor of 2. If none of the partitioned bins pass the minimum observation threshold, the index is -1. The shape array contains an interger indicating the number and position of valid bins (and therefore the number of bins, starting with the index number, containing data underneath the higher-level bin). Shape values are converted from a binary number with each bit representing whether or not each subpartition contains valid data, going left to right for the top row and then the bottom row. So a subdivision containing only data in the top-left bin would have a value of 2, whereas a completely full set of subpartitions would have a value of 15. The smallest binsize data does not have corresponding positions in the index array or shape array as there are no further sub-partitionings. Indices in the index array are relative to the data array start position, given in the ‘chrom index bounds’ portion of the header.

13.7 The fivec_binning module

This is a module contains scripts for generating compact, upper-triangle and full matrices of 5C interaction data.

13.7.1 Concepts

Data can either be arranged in compact, complete, or flattened (row-major) upper-triangle arrays. Compact arrays are N x M, where N is the number of forward probe fragments and M is the number of reverse probe fragments. Data can be raw, fragment-corrected, distance-dependence removed, or enrichment values. Arrays are 3-dimensional with observed values in the first layer of d3, expected values in the second layer of d3. The exception to this is upper-triangle arrays, which are 2d, dividing observed and expected along the second axis.

13.7.2 API documentation

`hifive.fivec_binning.`**`bin_cis_array`**(*data_array*, *data_mapping*, *binsize=10000*, *binbounds=None*, *start=None*, *stop=None*, *arraytype='full'*, *returnmapping=False*, ***kwargs*)

Create an array of format ‘arraytype’ and fill ‘binsize’ bins or bins defined by ‘binbounds’ with data provided in the array passed by ‘data_array’.

Parameters

- **data_array** (*numpy array*) – A 2d (upper) or 3d (full) array containing data to be binned. Array format will be determined from the number of dimensions.
- **data_mapping** (*numpy array*) – An N x 4 2d integer array containing the start and stop coordinates, and start and stop fragments for each of the N bin ranges in 'data_array'.
- **binsize** (*int.*) – This is the coordinate width of each bin. If binbounds is not None, this value is ignored.
- **binbounds** (*numpy array*) – An array containing start and stop coordinates for a set of user-defined bins. Any bin from 'data_array' not falling in a bin is ignored.
- **start** (*int.*) – The coordinate at the beginning of the first bin of the binned data. If unspecified, 'start' will be the first multiple of 'binsize' below the first coordinate from 'data_mapping'. If 'binbounds' is given, 'start' is ignored. Optional.
- **stop** (*int.*) – The coordinate at the end of the last bin of the binned data. If unspecified, 'stop' will be the first multiple of 'binsize' after the last coordinate from 'data_mapping'. If needed, 'stop' is adjusted upward to create a complete last bin. If 'binbounds' is given, 'stop' is ignored. Optional.
- **arraytype** (*str.*) – This determines what shape of array data are returned in. Acceptable values are 'full' and 'upper'. 'full' returns a square, symmetric array of size N x N x 2. 'upper' returns only the flattened upper triangle of a full array, excluding the diagonal of size (N * (N - 1) / 2) x 2.
- **returnmapping** (*bool.*) – If 'True', a list containing the data array and a 2d array containing first coordinate included and excluded from each bin, and the first fragment included and excluded from each bin is returned. Otherwise only the data array is returned.

Returns Array in format requested with 'arraytype' containing binned data requested with 'datatype' pulled from 'data_array' or list of binned data array and mapping array.

hifive.fivec_binning.**dynamically_bin_cis_array**(*unbinned*, *unbinnedpositions*, *binned*, *binbounds*, *minobservations=50*, *searchdistance=0*, *removefailed=True*, ***kwargs*)

Expand bins in 'binned' to include additional data provided in 'unbinned' as necessary to meet 'minobservations', or 'searchdistance' criteria.

Parameters

- **unbinned** (*numpy array*) – A full or upper array containing data to be binned. Array format will be determined from the number of dimensions.
- **unbinnedpositions** (*numpy array*) – A 2d integer array indicating the first and last coordinate of each bin in 'unbinned' array.
- **binned** (*numpy array*) – A full or upper array containing binned data to be dynamically binned. Array format will be determined from the number of dimensions. Data in this array will be altered by this function.
- **binbounds** (*numpy array*) – A N x 2 integer array indicating the start and end position of each of N bins in 'binned' array.
- **minobservations** (*int.*) – The fewest number of observed reads needed for a bin to counted as valid and stop expanding.
- **searchdistance** (*int.*) – The furthest distance from the bin minpoint to expand bounds. If this is set to zero, there is no limit on expansion distance.

- **removefailed** (*bool.*) – If a non-zero 'searchdistance' is given, it is possible for a bin not to meet the 'minobservations' criteria before stopping looking. If this occurs and 'removefailed' is True, the observed and expected values for that bin are zero.

Returns None

hifive.fivec_binning.**dynamically_bin_trans_array**(*unbinned*, *unbinnedpositions1*, *unbinnedpositions2*, *binned*, *binbounds1*, *binbounds2*, *minobservations=50*, *searchdistance=0*, *removefailed=True*, ***kwargs*)

Expand bins in 'binned' to include additional data provided in 'unbinned' as necessary to meet 'minobservations', or 'searchdistance' criteria.

Parameters

- **unbinned** (*numpy array*) – A full array containing data to be binned.
- **unbinnedpositions1** (*numpy array*) – A 2d integer array indicating the first and last coordinate of each bin in 'unbinned' array along the first axis.
- **unbinnedpositions2** (*numpy array*) – A 2d integer array indicating the first and last coordinate of each bin in 'unbinned' array along the second axis.
- **binned** (*numpy array*) – A full array containing binned data to be dynamically binned. Data in this array will be altered by this function.
- **binbounds1** (*numpy array*) – A N x 2 integer array indicating the start and end position of each of N bins in 'binned' array along the first axis.
- **binbounds2** (*numpy array*) – A N x 2 integer array indicating the start and end position of each of N bins in 'binned' array along the second axis.
- **minobservations** (*int.*) – The fewest number of observed reads needed for a bin to counted as valid and stop expanding.
- **searchdistance** (*int.*) – The furthest distance from the bin minpoint to expand bounds. If this is set to zero, there is no limit on expansion distance.
- **removefailed** (*bool.*) – If a non-zero 'searchdistance' is given, it is possible for a bin not to meet the 'minobservations' criteria before stopping looking. If this occurs and 'removefailed' is True, the observed and expected values for that bin are zero.

Returns None

hifive.fivec_binning.**find_cis_signal**(*fivec*, *region*, *binsize=0*, *binbounds=None*, *start=None*, *stop=None*, *startfrag=None*, *stopfrag=None*, *datatype='enrichment'*, *arraytype='full'*, *skipfiltered=False*, *returnmapping=False*, ***kwargs*)

Create an array of format 'arraytype' and fill with data requested in 'datatype'.

Parameters

- **fivec** (FiveC) – A FiveC class object containing fragment and count data.
- **region** (*int.*) – The index of the region to pull data from.
- **binsize** (*int.*) – This is the coordinate width of each bin. A value of zero indicates unbinned. If binbounds is not None, this value is ignored.
- **binbounds** (*numpy array*) – An array containing start and stop coordinates for a set of user-defined bins. Any fragment not falling in a bin is ignored.

- **start** (*int.*) – The smallest coordinate to include in the array, measured from fragment midpoints. If 'binbounds' is given, this value is ignored. If both 'start' and 'startfrag' are given, 'start' will override 'startfrag'. If unspecified, this will be set to the midpoint of the first fragment for 'region', adjusted to the first multiple of 'binsize' if not zero. Optional.
- **stop** (*int.*) – The largest coordinate to include in the array, measured from fragment midpoints. If 'binbounds' is given, this value is ignored. If both 'stop' and 'stopfrag' are given, 'stop' will override 'stopfrag'. If unspecified, this will be set to the midpoint of the last fragment plus one for 'region', adjusted to the last multiple of 'start' + 'binsize' if not zero. Optional.
- **startfrag** (*int.*) – The first fragment to include in the array. If 'binbounds' is given, this value is ignored. If unspecified and 'start' is not given, this is set to the first fragment in 'region'. In cases where 'start' is specified and conflicts with 'startfrag', 'start' is given preference. Optional.
- **stopfrag** (*int.*) – The first fragment not to include in the array. If 'binbounds' is given, this value is ignored. If unspecified and 'stop' is not given, this is set to the last fragment in 'region' plus one. In cases where 'stop' is specified and conflicts with 'stopfrag', 'stop' is given preference. Optional.
- **datatype** (*str.*) – This specifies the type of data that is processed and returned. Options are 'raw', 'distance', 'fragment', 'enrichment', and 'expected'. Observed values are aways in the first index along the last axis, except when 'datatype' is 'expected'. In this case, filter values replace counts. Conversely, if 'raw' is specified, unfiltered fragments return value of one. Expected values are returned for 'distance', 'fragment', 'enrichment', and 'expected' values of 'datatype'. 'distance' uses only the expected signal given distance for calculating the expected values, 'fragment' uses only fragment correction values, and both 'enrichment' and 'expected' use both correction and distance mean values. 'enrichment' also scales both observed and expected by the standard deviation, giving a completely normalized set of values.
- **arraytype** (*str.*) – This determines what shape of array data are returned in. Acceptable values are 'compact' (though only when 'binned' is zero), 'full', and 'upper'. 'compact' means data are arranged in a N x M x 2 array where N and M are the number of forward and reverse probe fragments, respectively. 'full' returns a square, symmetric array of size N x N x 2 where N is the total number of fragments. 'upper' returns only the flattened upper triangle of a full array, excluding the diagonal of size (N * (N - 1) / 2) x 2, where N is the total number of fragments.
- **skipfiltered** (*bool.*) – If 'True', all interaction bins for filtered out fragments are removed and a reduced-size array is returned.
- **returnmapping** (*bool.*) – If 'True', a list containing the data array and either one or two 2d arrays containing first coordinate included and excluded from each bin, and the first fragment included and excluded from each bin corresponding to both axes or the first and second axis for an upper or compact array, respectively, is returned. Otherwise only the data array is returned.

Returns Array in format requested with 'arraytype' containing data requested with 'datatype'.

`hifive.fivec_binning.`**`find_trans_signal`**(*fivec*, *region1*, *region2*, *binsize=0*, *binbounds1=None*, *start1=None*, *stop1=None*, *startfrag1=None*, *stopfrag1=None*, *binbounds2=None*, *start2=None*, *stop2=None*, *startfrag2=None*, *stopfrag2=None*, *datatype='enrichment'*, *arraytype='compact'*, *skipfiltered=False*, *returnmapping=False*, ***kwargs*)

Create an array of format 'arraytype' and fill with data requested in 'datatype'.

Parameters

- **fivec** (*FiveC*) – A *FiveC* class object containing fragment and count data.
- **region1** (*int.*) – The index of the first region to pull data from.
- **region2** (*int.*) – The index of the second region to pull data from.
- **binsize** (*int.*) – This is the coordinate width of each bin. A value of zero indicates unbinned. If binbounds is not None, this value is ignored.
- **binbounds1** (*numpy array*) – An array containing start and stop coordinates for a set of user-defined bins for region1. Any fragment not falling in a bin is ignored.
- **start1** (*int.*) – The smallest coordinate to include in the array from 'region1', measured from fragment midpoints. If 'binbounds1' is given, this value is ignored. If both 'start1' and 'startfrag1' are given, 'start1' will override 'startfrag1'. If unspecified, this will be set to the midpoint of the first fragment for 'region1', adjusted to the first multiple of 'binsize' if not zero. Optional.
- **stop1** (*int.*) – The largest coordinate to include in the array from 'region1', measured from fragment midpoints. If 'binbounds1' is given, this value is ignored. If both 'stop1' and 'stopfrag1' are given, 'stop1' will override 'stopfrag1'. If unspecified, this will be set to the midpoint of the last fragment plus one for 'region1', adjusted to the last multiple of 'start1' + 'binsize' if not zero. Optional.
- **startfrag1** (*int.*) – The first fragment to include in the array from 'region1'. If 'binbounds1' is given, this value is ignored. If unspecified and 'start1' is not given, this is set to the first fragment in 'region1'. In cases where 'start1' is specified and conflicts with 'startfrag1', 'start1' is given preference. Optional.
- **stopfrag1** (*int.*) – The first fragment not to include in the array from 'region1'. If 'binbounds1' is given, this value is ignored. If unspecified and 'stop1' is not given, this is set to the last fragment in 'region1' plus one. In cases where 'stop1' is specified and conflicts with 'stopfrag1', 'stop1' is given preference. Optional.
- **binbounds2** (*numpy array*) – An array containing start and stop coordinates for a set of user-defined bins for region2. Any fragment not falling in a bin is ignored.
- **start2** (*int.*) – The smallest coordinate to include in the array from 'region2', measured from fragment midpoints. If 'binbounds2' is given, this value is ignored. If both 'start2' and 'startfrag2' are given, 'start2' will override 'startfrag2'. If unspecified, this will be set to the midpoint of the first fragment for 'region2', adjusted to the first multiple of 'binsize' if not zero. Optional.
- **stop2** (*int.*) – The largest coordinate to include in the array from 'region2', measured from fragment midpoints. If 'binbounds2' is given, this value is ignored. If both 'stop2' and 'stopfrag2' are given, 'stop2' will override 'stopfrag2'. If unspecified, this will be set to the midpoint of the last fragment plus one for 'region2', adjusted to the last multiple of 'start2' + 'binsize' if not zero. Optional.
- **startfrag2** (*int.*) – The first fragment to include in the array from 'region2'. If 'binbounds2' is given, this value is ignored. If unspecified and 'start2' is not given, this is set to the first fragment in 'region2'. In cases where 'start2' is specified and conflicts with 'startfrag2', 'start2' is given preference. Optional.
- **stopfrag2** (*int.*) – The first fragment not to include in the array from 'region2'. If 'binbounds2' is given, this value is ignored. If unspecified and 'stop2' is not given, this is set to the last fragment in 'region2' plus one. In cases where 'stop2' is specified and conflicts with 'stopfrag2', 'stop2' is given preference. Optional.

- **datatype** (*str.*) – This specifies the type of data that is processed and returned. Options are 'raw', 'distance', 'fragment', 'enrichment', and 'expected'. Observed values are aways in the first index along the last axis, except when 'datatype' is 'expected'. In this case, filter values replace counts. Conversely, if 'raw' is specified, unfiltered fragments return value of one. Expected values are returned for 'distance', 'fragment', 'enrichment', and 'expected' values of 'datatype'. 'distance' uses only the expected signal given distance for calculating the expected values, 'fragment' uses only fragment correction values, and both 'enrichment' and 'expected' use both correction and distance mean values. 'enrichment' also scales both observed and expected by the standard deviation, giving a completely normalized set of values.
- **arraytype** (*str.*) – This determines what shape of array data are returned in. Acceptable values are 'compact' (though only when 'binned' is zero), and 'full'. 'compact' means data are arranged in a N x M x 2 array where N and M are the number of forward and reverse probe fragments, respectively. This will only return the array of forward primers from 'region1' and reverse primers from 'region2'. 'full' returns a square, symmetric array of size N x N x 2 where N is the total number of fragments.
- **skipfiltered** (*bool.*) – If 'True', all interaction bins for filtered out fragments are removed and a reduced-size array is returned.
- **returnmapping** (*bool.*) – If 'True', a list containing the data array and either one or four 2d arrays containing first coordinate included and excluded from each bin, and the first fragment included and excluded from each bin corresponding to both axes or the first and second axis for 'region1' forward fragments by 'region2' reverse fragments and 'region1' reverse fragments by 'region2' forward fragments for a full or compact array, respectively, is returned. Otherwise only the data array (or data arrays is compact) is returned.

Returns Array in format requested with 'arraytype' containing data requested with 'datatype'.

`hifive.fivec_binning.`**`write_heatmap_dict`**(*fivec*, *filename*, *binsize*, *includetrans=True*, *datatype='enrichment'*, *regions=[]*, *arraytype='full'*, *dynamically_binned=False*, *minobservations=0*, *searchdistance=0*, *expansion_binsize=0*, *removefailed=False*, ***kwargs*)

Create an h5dict file containing binned interaction arrays, bin positions, and an index of included regions.

Parameters

- **fivec** (`FiveC`) – A `FiveC` class object containing fragment and count data.
- **filename** (*str.*) – Location to write h5dict object to.
- **binsize** (*int.*) – Size of bins for interaction arrays. If "binsize" is zero, fragment interactions are returned without binning.
- **includetrans** (*bool.*) – Indicates whether trans interaction arrays should be calculated and saved.
- **datatype** – This specifies the type of data that is processed and returned. Options are 'raw', 'distance', 'fragment', 'enrichment', and 'expected'. Observed values are aways in the first index along the last axis, except when 'datatype' is 'expected'. In this case, filter values replace counts. Conversely, if 'raw' is specified, non-filtered bins return value of 1. Expected values are returned for 'distance', 'fragment', 'enrichment', and 'expected' values of 'datatype'. 'distance' uses only the expected signal given distance for calculating the expected values, 'fragment' uses only fragment correction values, and both 'enrichment' and 'expected' use both correction and distance mean values. :type datatype: str.
- **arraytype** (*str.*) – This determines what shape of array data are returned in if unbinned heatmaps are requested. Acceptable values are 'compact' and 'full'. 'compact' means data

are arranged in a N x M array where N is the number of bins, M is the maximum number of steps between included bin pairs, and data are stored such that bin n,m contains the interaction values between n and n + m + 1. 'full' returns a square, symmetric array of size N x N.

- **regions** (*list.*) – If given, indicates which regions should be included. If left empty, all regions are included.
- **dynamically_binned** (*bool.*) – If 'True', return dynamically binned data.
- **minobservations** (*int.*) – The fewest number of observed reads needed for a bin to counted as valid and stop expanding.
- **searchdistance** (*int.*) – The furthest distance from the bin minpoint to expand bounds. If this is set to zero, there is no limit on expansion distance.
- **expansion_binsize** (*int.*) – The size of bins to use for data to pull from when expanding dynamic bins. If set to zero, unbinned data is used.
- **removefailed** – If a non-zero 'searchdistance' is given, it is possible for a bin not to meet the 'minobservations' criteria before stopping looking. If this occurs and 'removefailed' is True, the observed and expected values for that bin are zero.

Returns None

13.8 The hic_binning module

This is a module contains scripts for generating compact, upper-triangle and full matrices of HiC interaction data.

13.8.1 Concepts

These functions rely on the `HiC` class in conjunction with the `Fend` and `HiCData` classes.

Data can either be arranged in compact, complete, or flattened (row-major) upper-triangle arrays. Compact arrays are N x M, where N is the number of fends or bins, and M is the maximum distance between fends or bins. This is useful for working with sets of short interactions. Data can be raw, fend-corrected, distance-dependence removed, or enrichment values. Arrays are 3-dimensional with observed values in the first layer of d3, expected values in the second layer of d3. The exception to this is upper-triangle arrays, which are 2d, divinding observed and expected along the second axis.

13.8.2 API Documentation

`hifive.hic_binning.`**`bin_cis_array`**(*data_array*, *data_mapping*, *binsize=10000*, *binbounds=None*, *start=None*, *stop=None*, *arraytype='full'*, *returnmapping=False*, ***kwargs*)

Create an array of format 'arraytype' and fill 'binsize' bins or bins defined by 'binbounds' with data provided in the array passed by 'data_array'.

Parameters

- **data_array** (*numpy array*) – A 2d (upper) or 3d (compact) array containing data to be binned. Array format will be determined from the number of dimensions.
- **data_mapping** (*numpy array*) – An N x 4 2d integer array containing the start and stop coordinates, and start and stop fends for each of the N bin ranges in 'data_array'.

- **binsize** (*int.*) – This is the coordinate width of each bin. If binbounds is not None, this value is ignored.
- **binbounds** (*numpy array*) – An array containing start and stop coordinates for a set of user-defined bins. Any bin from 'data_array' not falling in a bin is ignored.
- **start** (*int.*) – The coordinate at the beginning of the first bin of the binned data. If unspecified, 'start' will be the first multiple of 'binsize' below the first coordinate from 'data_mapping'. If 'binbounds' is given, 'start' is ignored. Optional.
- **stop** (*int.*) – The coordinate at the end of the last bin of the binned data. If unspecified, 'stop' will be the first multiple of 'binsize' after the last coordinate from 'data_mapping'. If needed, 'stop' is adjusted upward to create a complete last bin. If 'binbounds' is given, 'stop' is ignored. Optional.
- **arraytype** (*str.*) – This determines what shape of array data are returned in. Acceptable values are 'compact', 'full', and 'upper'. 'compact' means data are arranged in a N x M x 2 array where N is the number of bins, M is the maximum number of steps between included bin pairs, and data are stored such that bin n,m contains the interaction values between n and n + m + 1. 'full' returns a square, symmetric array of size N x N x 2. 'upper' returns only the flattened upper triangle of a full array, excluding the diagonal of size (N * (N - 1) / 2) x 2.
- **returnmapping** (*bool.*) – If 'True', a list containing the data array and a 2d array containing first coordinate included and excluded from each bin, and the first fend included and excluded from each bin is returned. Otherwise only the data array is returned.

Returns Array in format requested with 'arraytype' containing binned data requested with 'datatype' pulled from 'data_array' or list of binned data array and mapping array.

`hifive.hic_binning.`**`bin_trans_array`**(*data_array*, *data_mapping1*, *data_mapping2*, *binsize=10000*, *binbounds1=None*, *start1=None*, *stop1=None*, *binbounds2=None*, *start2=None*, *stop2=None*, *returnmapping=False*, ***kwargs*)

Create an array of format 'arraytype' and fill 'binsize' bins or bins defined by 'binbounds' with data provided in the array passed by 'unbinned'.

Parameters

- **hic** (*HiC*) – A *HiC* class object containing fend and count data.
- **data_array** (*numpy array*) – A 3d array containing data to be binned.
- **data_mapping1** (*numpy array*) – An N x 4 2d integer array containing the start and stop coordinates, and start and stop fends for each of the N bin ranges along the first axis in 'data_array'.
- **data_mapping2** (*numpy array*) – An N x 4 2d integer array containing the start and stop coordinates, and start and stop fends for each of the N bin ranges along the second axis in 'data_array'.
- **binsize** (*int.*) – This is the coordinate width of each bin. If binbounds is not None, this value is ignored.
- **binbounds1** (*numpy array*) – An array containing start and stop coordinates for a set of user-defined bins along the first axis. Any bin from 'data_array' not falling in a bin is ignored.
- **start1** (*int.*) – The coordinate at the beginning of the first bin for the first axis of the binned data. If unspecified, 'start1' will be the first multiple of 'binsize' below the first coordinate from 'data_mapping1'. If 'binbounds1' is given, 'start1' is ignored. Optional.

- **stop1** (*int.*) – The coordinate at the end of the last bin for the first axis of the binned data. If unspecified, 'stop1' will be the first multiple of 'binsize' after the last coordinate from 'data_mapping1'. If needed, 'stop1' is adjusted upward to create a complete last bin. If 'binbounds1' is given, 'stop1' is ignored. Optional.
- **binbounds2** (*numpy array*) – An array containing start and stop coordinates for a set of user-defined bins along the second axis. Any bin from 'data_array' not falling in a bin is ignored.
- **start2** (*int.*) – The coordinate at the beginning of the first bin for the second axis of the binned data. If unspecified, 'start2' will be the first multiple of 'binsize' below the first coordinate from 'data_mapping2'. If 'binbounds2' is given, 'start2' is ignored. Optional.
- **stop2** (*int.*) – The coordinate at the end of the last bin for the second axis of the binned data. If unspecified, 'stop2' will be the first multiple of 'binsize' after the last coordinate from 'data_mapping2'. If needed, 'stop2' is adjusted upward to create a complete last bin. If 'binbounds2' is given, 'stop2' is ignored. Optional.
- **datatype** (*str.*) – This specifies the type of data that is processed and returned. Options are 'raw', 'distance', 'fend', 'enrichment', and 'expected'. Observed values are always in the first index along the last axis, except when 'datatype' is 'expected'. In this case, filter values replace counts. Conversely, if 'raw' is specified, unfiltered fends return value of one. Expected values are returned for 'distance', 'fend', 'enrichment', and 'expected' values of 'datatype'. 'distance' uses only the expected signal given distance for calculating the expected values, 'fend' uses only fend correction values, and both 'enrichment' and 'expected' use both correction and distance mean values.
- **returnmapping** (*bool.*) – If 'True', a list containing the data array and a 2d array containing first coordinate included and excluded from each bin, and the first fend included and excluded from each bin is returned. Otherwise only the data array is returned.

Returns Array in format requested with 'arraytype' containing binned data requested with 'datatype' pulled from 'unbinned'.

hifive.hic_binning.**dynamically_bin_cis_array**(*unbinned*, *unbinnedpositions*, *binned*, *binbounds*, *minobservations=10*, *searchdistance=0*, *removefailed=True*, ***kwargs*)

Expand bins in 'binned' to include additional data provided in 'unbinned' as necessary to meet 'minobservations', or 'searchdistance' criteria.

Parameters

- **unbinned** (*numpy array*) – A 2d or 3d array containing data in either compact or upper format to be used for filling expanding bins. Array format will be determined from the number of dimensions.
- **unbinnedpositions** (*numpy array*) – A 2d integer array indicating the first and last coordinate of each bin in 'unbinned' array.
- **binned** (*numpy array*) – A 2d or 3d array containing binned data in either compact or upper format to be dynamically binned. Array format will be determined from the number of dimensions. Data in this array will be altered by this function.
- **binbounds** (*numpy array*) – An integer array indicating the start and end position of each bin in 'binned' array. This array should be N x 2, where N is the number of intervals in 'binned'.
- **minobservations** (*int.*) – The fewest number of observed reads needed for a bin to counted as valid and stop expanding.

- **searchdistance** (*int.*) – The furthest distance from the bin minpoint to expand bounds. If this is set to zero, there is no limit on expansion distance.
- **removefailed** (*bool.*) – If a non-zero 'searchdistance' is given, it is possible for a bin not to meet the 'minobservations' criteria before stopping looking. If this occurs and 'removefailed' is True, the observed and expected values for that bin are zero.

Returns None

hifive.hic_binning.**dynamically_bin_trans_array**(*unbinned*, *unbinnedpositions1*, *unbinnedpositions2*, *binned*, *binbounds1*, *binbounds2*, *minobservations=10*, *searchdistance=0*, *removefailed=False*, ***kwargs*)

Expand bins in 'binned' to include additional data provided in 'unbinned' as necessary to meet 'minobservations', or 'searchdistance' criteria.

Parameters

- **unbinned** (*numpy array*) – A 3d array containing data to be used for filling expanding bins. This array should be N x M x 2, where N is the number of bins or fends from the first chromosome and M is the number of bins or fends from the second chromosome.
- **unbinnedpositions1** (*numpy array*) – A 2d integer array indicating the first and last coordinate of each bin along the first axis in 'unbinned' array.
- **unbinnedpositions2** (*numpy array*) – A 2d integer array indicating the first and last coordinate of each bin along the first axis in 'unbinned' array.
- **binned** (*numpy array*) – A 3d array containing binned data to be dynamically binned. This array should be N x M x 2, where N is the number of bins from the first chromosome and M is the number of bins from the second chromosome. Data in this array will be altered by this function.
- **binbounds1** (*numpy array*) – An integer array indicating the start and end position of each bin from the first chromosome in the 'binned' array. This array should be N x 2, where N is the size of the first dimension of 'binned'.
- **binbounds2** (*numpy array*) – An integer array indicating the start and end position of each bin from the second chromosome in the 'binned' array. This array should be N x 2, where N is the size of the second dimension of 'binned'.
- **minobservations** (*int.*) – The fewest number of observed reads needed for a bin to counted as valid and stop expanding.
- **searchdistance** (*int.*) – The furthest distance from the bin minpoint to expand bounds. If this is set to zero, there is no limit on expansion distance.
- **removefailed** (*bool.*) – If a non-zero 'searchdistance' is given, it is possible for a bin not to meet the 'minobservations' criteria before stopping looking. If this occurs and 'removefailed' is True, the observed and expected values for that bin are zero.

Returns None

hifive.hic_binning.**find_cis_signal**(*hic*, *chrom*, *binsize=10000*, *binbounds=None*, *start=None*, *stop=None*, *startfend=None*, *stopfend=None*, *datatype='enrichment'*, *arraytype='compact'*, *maxdistance=0*, *skipfiltered=False*, *returnmapping=False*, *proportional=False*, ***kwargs*)

Create an array of format 'arraytype' and fill with data requested in 'datatype'.

Parameters

- **hic** (*HiC*) – A *HiC* class object containing fend and count data.
- **chrom** (*str.*) – The name of a chromosome contained in 'hic'.
- **binsize** (*int.*) – This is the coordinate width of each bin. A value of zero indicates unbinned. If binbounds is not None, this value is ignored.
- **binbounds** (*numpy array*) – An array containing start and stop coordinates for a set of user-defined bins. Any fend not falling in a bin is ignored.
- **start** (*int.*) – The smallest coordinate to include in the array, measured from fend midpoints or the start of the first bin. If 'binbounds' is given, this value is ignored. If both 'start' and 'startfend' are given, 'start' will override 'startfend'. If unspecified, this will be set to the midpoint of the first fend for 'chrom', adjusted to the first multiple of 'binsize' if not zero. Optional.
- **stop** (*int.*) – The largest coordinate to include in the array, measured from fend midpoints or the end of the last bin. If 'binbounds' is given, this value is ignored. If both 'stop' and 'stopfend' are given, 'stop' will override 'stopfend'. If unspecified, this will be set to the midpoint of the last fend plus one for 'chrom', adjusted to the last multiple of 'start' + 'binsize' if not zero. Optional.
- **startfend** (*int.*) – The first fend to include in the array. If 'binbounds' is given, this value is ignored. If unspecified and 'start' is not given, this is set to the first valid fend in 'chrom'. In cases where 'start' is specified and conflicts with 'startfend', 'start' is given preference. Optional
- **stopfend** (*str.*) – The first fend not to include in the array. If 'binbounds' is given, this value is ignored. If unspecified and 'stop' is not given, this is set to the last valid fend in 'chrom' plus one. In cases where 'stop' is specified and conflicts with 'stopfend', 'stop' is given preference. Optional.
- **datatype** (*str.*) – This specifies the type of data that is processed and returned. Options are 'raw', 'distance', 'fend', 'enrichment', and 'expected'. Observed values are always in the first index along the last axis, except when 'datatype' is 'expected'. In this case, filter values replace counts. Conversely, if 'raw' is specified, unfiltered fends return value of one. Expected values are returned for 'distance', 'fend', 'enrichment', and 'expected' values of 'datatype'. 'distance' uses only the expected signal given distance for calculating the expected values, 'fend' uses only fend correction values, and both 'enrichment' and 'expected' use both correction and distance mean values.
- **arraytype** (*str.*) – This determines what shape of array data are returned in. Acceptable values are 'compact', 'full', and 'upper'. 'compact' means data are arranged in a N x M x 2 array where N is the number of bins, M is the maximum number of steps between included bin pairs, and data are stored such that bin n,m contains the interaction values between n and n + m + 1. 'full' returns a square, symmetric array of size N x N x 2. 'upper' returns only the flattened upper triangle of a full array, excluding the diagonal of size (N * (N - 1) / 2) x 2.
- **maxdistance** (*str.*) – This specifies the maximum coordinate distance between bins that will be included in the array. If set to zero, all distances are included.
- **skipfiltered** (*bool.*) – If 'True', all interaction bins for filtered out fends are removed and a reduced-size array is returned.
- **returnmapping** (*bool.*) – If 'True', a list containing the data array and a 2d array containing first coordinate included and excluded from each bin, and the first fend included and excluded from each bin is returned. Otherwise only the data array is returned.

- **proportional** (*bool.*) – Indicates whether interactions should proportionally contribute to bins based on the amount of overlap instead of being attributed solely based on midpoint. Only valid for binned heatmaps.

Returns Array in format requested with 'arraytype' containing data requested with 'datatype'.

hifive.hic_binning.**find_multiresolution_heatmap**(*hic*, *chrom*, *start*, *stop*, *chrom2=None*, *start2=None*, *stop2=None*, *minbinsize=5000*, *maxbinsize=12800000*, *minobservations=5*, *datatype='fend'*, *midbinsize=40000*, *silent=True*)

Create a multi-resolution data and index heatmap array for a chromosome or chromosome pair.

Parameters

- **hic** (*HiC*) – A *HiC* class object containing fend and count data.
- **chrom** (*str.*) – The first (or only) chromosome to find the multi-resolution heatmap for.
- **start** (*int.*) – The first bin start coordinate.
- **stop** (*int.*) – The last bin stop coordinate. The difference between start and stop must be a multiple of maxbinsize.
- **chrom2** (*str.*) – The second chromosome to find the multi-resolution heatmap for. If None, an intra-chromosomal multi-resolution heatmap is returned for chrom.
- **start2** (*int.*) – The first bin start coordinate for the second chromosome.
- **stop2** (*int.*) – The last bin stop coordinate for the second chromosome. The difference between start and stop must be a multiple of maxbinsize.
- **maxbinsize** (*int.*) – The maximum sized bin (lowest resolution) heatmap to be produced for each chromosome.
- **minbinsize** (*int.*) – The minimum sized bin (highest resolution) heatmap to be produced for each chromosome.
- **minobservations** (*int.*) – The minimum number of reads needed for a bin to be considered valid and be included in the heatmap.
- **datatype** (*str.*) – This specifies the type of data that is processed and returned. Options are 'raw', 'distance', 'fend', and 'enrichment'. Observed values are always in the first index along the last axis. If 'raw' is specified, unfiltered fends return value of one. Expected values are returned for 'distance', 'fend', 'enrichment', and 'expected' values of 'datatype'. 'distance' uses only the expected signal given distance for calculating the expected values, 'fend' uses only fend correction values, and 'enrichment' uses both correction and distance mean values.
- **midbinsize** (*int.*) – This is used to determine the smallest bin size (highest resolution) complete heatmap to generate in producing the multi-resolution heatmap. It does not affect the resulting output but can be used to limit the total memory usage, with higher values using less memory but more time.
- **silent** (*bool.*) – Indicates whether to display messages or not.

hifive.hic_binning.**find_trans_signal**(*hic*, *chrom1*, *chrom2*, *binsize=10000*, *binbounds1=None*, *binbounds2=None*, *start1=None*, *stop1=None*, *startfend1=None*, *stopfend1=None*, *start2=None*, *stop2=None*, *startfend2=None*, *stopfend2=None*, *datatype='enrichment'*, *skipfiltered=False*, *returnmapping=False*, ***kwargs*)

Create an array of format 'arraytype' and fill with data requested in 'datatype'.

Parameters

- **hic** (*HiC*) – A *HiC* class object containing fend and count data.
- **chrom** (*str.*) – The name of a chromosome contained in 'hic'.
- **binsize** (*int.*) – This is the coordinate width of each bin. A value of zero indicates unbinned. If binbounds is not None, this value is ignored.
- **binbounds** (*numpy array*) – An array containing start and stop coordinates for a set of user-defined bins. Any fend not falling in a bin is ignored.
- **start** (*int.*) – The smallest coordinate to include in the array, measured from fend midpoints or the start of the first bin. If 'binbounds' is given, this value is ignored. If both 'start' and 'startfend' are given, 'start' will override 'startfend'. If unspecified, this will be set to the midpoint of the first fend for 'chrom', adjusted to the first multiple of 'binsize' if not zero. Optional.
- **stop** (*int.*) – The largest coordinate to include in the array, measured from fend midpoints or the end of the last bin. If 'binbounds' is given, this value is ignored. If both 'stop' and 'stopfend' are given, 'stop' will override 'stopfend'. If unspecified, this will be set to the midpoint of the last fend plus one for 'chrom', adjusted to the last multiple of 'start' + 'binsize' if not zero. Optional.
- **startfend** (*int.*) – The first fend to include in the array. If 'binbounds' is given, this value is ignored. If unspecified and 'start' is not given, this is set to the first valid fend in 'chrom'. In cases where 'start' is specified and conflicts with 'startfend', 'start' is given preference. Optional
- **stopfend** (*str.*) – The first fend not to include in the array. If 'binbounds' is given, this value is ignored. If unspecified and 'stop' is not given, this is set to the last valid fend in 'chrom' plus one. In cases where 'stop' is specified and conflicts with 'stopfend', 'stop' is given preference. Optional.
- **datatype** (*str.*) – This specifies the type of data that is processed and returned. Options are 'raw', 'distance', 'fend', 'enrichment', and 'expected'. Observed values are always in the first index along the last axis, except when 'datatype' is 'expected'. In this case, filter values replace counts. Conversely, if 'raw' is specified, unfiltered fends return value of one. Expected values are returned for 'distance', 'fend', 'enrichment', and 'expected' values of 'datatype'. 'distance' uses only the expected signal given distance for calculating the expected values, 'fend' uses only fend correction values, and both 'enrichment' and 'expected' use both correction and distance mean values.
- **arraytype** (*str.*) – This determines what shape of array data are returned in. Acceptable values are 'compact', 'full', and 'upper'. 'compact' means data are arranged in a N x M x 2 array where N is the number of bins, M is the maximum number of steps between included bin pairs, and data are stored such that bin n,m contains the interaction values between n and n + m + 1. 'full' returns a square, symmetric array of size N x N x 2. 'upper' returns only the flattened upper triangle of a full array, excluding the diagonal of size (N * (N - 1) / 2) x 2.
- **maxdistance** (*str.*) – This specifies the maximum coordinate distance between bins that will be included in the array. If set to zero, all distances are included.
- **skipfiltered** (*bool.*) – If 'True', all interaction bins for filtered out fends are removed and a reduced-size array is returned.
- **returnmapping** (*bool.*) – If 'True', a list containing the data array and two 2d array containing first coordinate included and excluded from each bin, and the first fend included

and excluded from each bin for the first and second axis is returned. Otherwise only the data array is returned.

Returns Array in format requested with 'arraytype' containing data requested with 'datatype'.

`hifive.hic_binning.`**`write_heatmap_dict`**(*hic*, *filename*, *binsize*, *includetrans=True*, *datatype='enrichment'*, *chroms=[]*, *dynamically_binned=False*, *minobservations=0*, *searchdistance=0*, *expansion_binsize=0*, *removefailed=False*, ***kwargs*)

Create an h5dict file containing binned interaction arrays, bin positions, and an index of included chromosomes. This function is MPI compatible.

Parameters

- **`hic`** (`HiC`) – A `HiC` class object containing fend and count data.
- **`filename`** (*str.*) – Location to write h5dict object to.
- **`binsize`** (*int.*) – Size of bins for interaction arrays.
- **`includetrans`** (*bool.*) – Indicates whether trans interaction arrays should be calculated and saved.
- **`datatype`** (*str.*) – This specifies the type of data that is processed and returned. Options are 'raw', 'distance', 'fend', 'enrichment', and 'expected'. Observed values are always in the first index along the last axis, except when 'datatype' is 'expected'. In this case, filter values replace counts. Conversely, if 'raw' is specified, unfiltered fends return value of one. Expected values are returned for 'distance', 'fend', 'enrichment', and 'expected' values of 'datatype'. 'distance' uses only the expected signal given distance for calculating the expected values, 'fend' uses only fend correction values, and both 'enrichment' and 'expected' use both correction and distance mean values.
- **`chroms`** (*list*) – A list of chromosome names indicating which chromosomes should be included. If left empty, all chromosomes are included. Optional.
- **`dynamically_binned`** (*bool.*) – If 'True', return dynamically binned data.
- **`minobservations`** (*int.*) – The fewest number of observed reads needed for a bin to counted as valid and stop expanding.
- **`searchdistance`** (*int.*) – The furthest distance from the bin minpoint to expand bounds. If this is set to zero, there is no limit on expansion distance.
- **`expansion_binsize`** (*int.*) – The size of bins to use for data to pull from when expanding dynamic bins. If set to zero, unbinned data is used.
- **`removefailed`** (*bool.*) – If a non-zero 'searchdistance' is given, it is possible for a bin not to meet the 'minobservations' criteria before stopping looking. If this occurs and 'removefailed' is True, the observed and expected values for that bin are zero.

Returns None

13.9 The plotting module

This is a module contains scripts for generating plots from compact and full matrices of interaction data.

13.9.1 Concepts

These functions take either compact, upper-triangle, or full 3d data matrices.

Data can either be arranged in compact, upper-triangle, or complete (rectangular) arrays. With HiC data, compact arrays are N x M x 2, where N is the number of fends or bins, and M is the maximum distance between fends or bins. This is useful for working with sets of short interactions. When using 5C data, the compact format is an N x M x 2 array where N is the number of forward primers and M is the number of reverse primers. Data can be raw, fend-corrected, distance-dependence removed, or enrichment values. Arrays are 3-dimensional with observed values in the first layer of d3, expected values in the second layer of d3.

13.9.2 API Documentation

`hifive.plotting.`**`plot_compact_array`**(*data*, *maxscore=None*, *minscore=None*, *symmetricscaling=True*, *logged=True*, *min_color='0000ff'*, *mid_color='ffffff'*, *max_color='ff0000'*, *returnscale=False*, ***kwargs*)

Fill in and rescale bitmap from a HiC compact array.

Parameters

- **data** (*numpy array*) – A three-dimensional compact array of HiC interaction data.
- **maxscore** (*float*) – A ceiling value to cutoff scores at for plot color.
- **minscore** (*float*) – A floor value to cutoff scores at for plot color.
- **symmetricscaling** (*bool.*) – Indicates whether to recenter data for scaling or maintain scores about zero.
- **logged** (*bool.*) – Indicates whether to use log2 values of scores for color values.
- **min_color** (*str.*) – This is a hex color code ("rrggbb" where each pair ranges from 00-ff) specifying the color associated with the minimum plot value. This variable is used to create a color gradient for plotting along with max_color and mid_color.
- **mid_color** (*str.*) – This is a hex color code ("rrggbb" where each pair ranges from 00-ff) specifying the color associated with the minimum plot value. This can be set to None to create a gradient ranging from min_color to max_color or to a hex color to create a divergent gradient.
- **max_color** (*str.*) – This is a hex color code ("rrggbb" where each pair ranges from 00-ff) specifying the color associated with the maximum plot value. This variable is used to create a color gradient for plotting along with min_color and mid_color.
- **returnscale** (*bool.*) – Indicates whether to return a list containing the bitmap, minimum score, and maximum score, or just the bitmap.

Returns `PIL` bitmap object and if requested, minimum and maximum scores.

`hifive.plotting.`**`plot_diagonal_from_compact_array`**(*data*, *maxscore=None*, *minscore=None*, *symmetricscaling=True*, *logged=True*, *min_color='0000ff'*, *mid_color='ffffff'*, *max_color='ff0000'*, *returnscale=False*, ***kwargs*)

Fill in and rescale bitmap from a HiC compact array, plotting only the upper triangle rotated 45 degrees counter-clockwise.

Parameters

- **data** (*numpy array*) – A three-dimensional compact array of HiC interaction data.
- **maxscore** (*float*) – A ceiling value to cutoff scores at for plot color.
- **minscore** (*float*) – A floor value to cutoff scores at for plot color.
- **symmetricscaling** (*bool.*) – Indicates whether to recenter data for scaling or maintain scores about zero.
- **logged** (*bool.*) – Indicates whether to use log2 values of scores for color values.
- **min_color** (*str.*) – This is a hex color code ("rrggbb" where each pair ranges from 00-ff) specifying the color associated with the minimum plot value. This variable is used to create a color gradient for plotting along with max_color and mid_color.
- **mid_color** (*str.*) – This is a hex color code ("rrggbb" where each pair ranges from 00-ff) specifying the color associated with the minimum plot value. This can be set to None to create a gradient ranging from min_color to max_color or to a hex color to create a divergent gradient.
- **max_color** (*str.*) – This is a hex color code ("rrggbb" where each pair ranges from 00-ff) specifying the color associated with the maximum plot value. This variable is used to create a color gradient for plotting along with min_color and mid_color.
- **returnscale** (*bool.*) – Indicates whether to return a list containing the bitmap, minimum score, and maximum score, or just the bitmap.

Returns `PIL` bitmap object and if requested, minimum and maximum scores.

`hifive.plotting.`**`plot_diagonal_from_upper_array`**(*data*, *maxscore=None*, *minscore=None*, *symmetricscaling=True*, *logged=True*, *min_color='0000ff'*, *mid_color='ffffff'*, *max_color='ff0000'*, *returnscale=False*, ***kwargs*)

Fill in and rescale bitmap from a HiC upper array, plotting only the upper triangle rotated 45 degrees counter-clockwise.

Parameters

- **data** (*numpy array*) – A three-dimensional upper array of HiC interaction data.
- **maxscore** (*float*) – A ceiling value to cutoff scores at for plot color.
- **minscore** (*float*) – A floor value to cutoff scores at for plot color.
- **symmetricscaling** (*bool.*) – Indicates whether to recenter data for scaling or maintain scores about zero.
- **logged** (*bool.*) – Indicates whether to use log2 values of scores for color values.
- **min_color** (*str.*) – This is a hex color code ("rrggbb" where each pair ranges from 00-ff) specifying the color associated with the minimum plot value. This variable is used to create a color gradient for plotting along with max_color and mid_color.
- **mid_color** (*str.*) – This is a hex color code ("rrggbb" where each pair ranges from 00-ff) specifying the color associated with the minimum plot value. This can be set to None to create a gradient ranging from min_color to max_color or to a hex color to create a divergent gradient.
- **max_color** (*str.*) – This is a hex color code ("rrggbb" where each pair ranges from 00-ff) specifying the color associated with the maximum plot value. This variable is used to create a color gradient for plotting along with min_color and mid_color.

- **returnscale** (*bool.*) – Indicates whether to return a list containing the bitmap, minimum score, and maximum score, or just the bitmap.

Returns PIL bitmap object and if requested, minimum and maximum scores.

hifive.plotting.**plot_fivec_compact_heatmap_dict**(*filename*, *maxscore=None*, *minscore=None*, *symmetricscaling=True*, *logged=True*, *regions=[]*, *min_color='0000ff'*, *mid_color='ffffff'*, *max_color='ff0000'*, *returnscale=False*, ***kwargs*)

Fill in and rescale bitmap in a compact from a 5C heatmap h5dict file.

This plots the data in a 5C compact format such that the rows correspond to positive-strand primers and columns correspond to negative-strand primers.

Parameters

- **filename** – Location of a heatmap h5dict containing 5C data arrays.
- **maxscore** (*float*) – A ceiling value to cutoff scores at for plot color.
- **minscore** (*float*) – A floor value to cutoff scores at for plot color.
- **symmetricscaling** (*bool.*) – Indicates whether to recenter data for scaling or maintain scores about zero.
- **logged** (*bool.*) – Indicates whether to use log2 values of scores for color values.
- **regions** (*list*) – If specified, only the indicated regions are plotted. Otherwise all regions present in the h5dict are plotted.
- **min_color** (*str.*) – This is a hex color code ("rrggbb" where each pair ranges from 00-ff) specifying the color associated with the minimum plot value. This variable is used to create a color gradient for plotting along with max_color and mid_color.
- **mid_color** (*str.*) – This is a hex color code ("rrggbb" where each pair ranges from 00-ff) specifying the color associated with the minimum plot value. This can be set to None to create a gradient ranging from min_color to max_color or to a hex color to create a divergent gradient.
- **max_color** (*str.*) – This is a hex color code ("rrggbb" where each pair ranges from 00-ff) specifying the color associated with the maximum plot value. This variable is used to create a color gradient for plotting along with min_color and mid_color.
- **returnscale** (*bool.*) – Indicates whether to return a list containing the bitmap, minimum score, and maximum score, or just the bitmap.

Returns PIL bitmap object and if requested, minimum and maximum scores.

hifive.plotting.**plot_fivec_heatmap**(*filename*, *maxscore=None*, *minscore=None*, *symmetricscaling=True*, *logged=True*, *regions=[]*, *min_color='0000ff'*, *mid_color='ffffff'*, *max_color='ff0000'*, *returnscale=False*, ***kwargs*)

Fill in and rescale bitmap in a full format from a 5C heatmap h5dict file.

Parameters

- **filename** – Location of a heatmap h5dict containing 5C data arrays.
- **maxscore** (*float*) – A ceiling value to cutoff scores at for plot color.
- **minscore** (*float*) – A floor value to cutoff scores at for plot color.

- **symmetricscaling** (*bool.*) – Indicates whether to recenter data for scaling or maintain scores about zero.
- **logged** (*bool.*) – Indicates whether to use log2 values of scores for color values.
- **regions** (*list*) – If specified, only the indicated regions are plotted. Otherwise all regions present in the h5dict are plotted.
- **min_color** (*str.*) – This is a hex color code ("rrggbb" where each pair ranges from 00-ff) specifying the color associated with the minimum plot value. This variable is used to create a color gradient for plotting along with max_color and mid_color.
- **mid_color** (*str.*) – This is a hex color code ("rrggbb" where each pair ranges from 00-ff) specifying the color associated with the minimum plot value. This can be set to None to create a gradient ranging from min_color to max_color or to a hex color to create a divergent gradient.
- **max_color** (*str.*) – This is a hex color code ("rrggbb" where each pair ranges from 00-ff) specifying the color associated with the maximum plot value. This variable is used to create a color gradient for plotting along with min_color and mid_color.
- **returnscale** (*bool.*) – Indicates whether to return a list containing the bitmap, minimum score, and maximum score, or just the bitmap.

Returns `PIL` bitmap object and if requested, minimum and maximum scores.

`hifive.plotting.`**`plot_full_array`**(*data*, *maxscore=None*, *minscore=None*, *symmetricscaling=True*, *logged=True*, *min_color='0000ff'*, *mid_color='ffffff'*, *max_color='ff0000'*, *returnscale=False*, ***kwargs*)

Fill in and rescale bitmap from a 5C or HiC full array.

Parameters

- **data** (*numpy array*) – A three-dimensional compact array of interaction data.
- **maxscore** (*float*) – A ceiling value to cutoff scores at for plot color.
- **minscore** (*float*) – A floor value to cutoff scores at for plot color.
- **symmetricscaling** (*bool.*) – Indicates whether to recenter data for scaling or maintain scores about zero.
- **logged** (*bool.*) – Indicates whether to use log2 values of scores for color values.
- **min_color** (*str.*) – This is a hex color code ("rrggbb" where each pair ranges from 00-ff) specifying the color associated with the minimum plot value. This variable is used to create a color gradient for plotting along with max_color and mid_color.
- **mid_color** (*str.*) – This is a hex color code ("rrggbb" where each pair ranges from 00-ff) specifying the color associated with the minimum plot value. This can be set to None to create a gradient ranging from min_color to max_color or to a hex color to create a divergent gradient.
- **max_color** (*str.*) – This is a hex color code ("rrggbb" where each pair ranges from 00-ff) specifying the color associated with the maximum plot value. This variable is used to create a color gradient for plotting along with min_color and mid_color.
- **returnscale** (*bool.*) – Indicates whether to return a list containing the bitmap, minimum score, and maximum score, or just the bitmap.

Returns `PIL` bitmap object and if requested, minimum and maximum scores.

hifive.plotting.**plot_hic_heatmap**(*filename*, *maxscore=None*, *minscore=None*, *symmetricscaling=True*, *logged=True*, *chroms=[]*, *min_color='0000ff'*, *mid_color='ffffff'*, *max_color='ff0000'*, *returnscale=False*, ***kwargs*)

Fill in and rescale bitmap from a HiC heatmap h5dict file.

Parameters

- **filename** (*str.*) – File name of heatmap h5dict containing binned data arrays.
- **maxscore** (*float*) – A ceiling value to cutoff scores at for plot color.
- **minscore** (*float*) – A floor value to cutoff scores at for plot color.
- **symmetricscaling** (*bool.*) – Indicates whether to recenter data for scaling or maintain scores about zero.
- **logged** (*bool.*) – Indicates whether to use log2 values of scores for color values.
- **chroms** (*list*) – A list of chromosome names to include in the plot. If left empty, all chromosomes present in the heatmap file will be plotted.
- **min_color** (*str.*) – This is a hex color code ("rrggbb" where each pair ranges from 00-ff) specifying the color associated with the minimum plot value. This variable is used to create a color gradient for plotting along with max_color and mid_color.
- **mid_color** (*str.*) – This is a hex color code ("rrggbb" where each pair ranges from 00-ff) specifying the color associated with the minimum plot value. This can be set to None to create a gradient ranging from min_color to max_color or to a hex color to create a divergent gradient.
- **max_color** (*str.*) – This is a hex color code ("rrggbb" where each pair ranges from 00-ff) specifying the color associated with the maximum plot value. This variable is used to create a color gradient for plotting along with min_color and mid_color.
- **returnscale** (*bool.*) – Indicates whether to return a list containing the bitmap, minimum score, and maximum score, or just the bitmap.

Returns `PIL` bitmap object and if requested, minimum and maximum scores.

hifive.plotting.**plot_key**(*min_score*, *max_score*, *height*, *width*, *labelformat='%0.2f'*, *orientation='left'*, *num_ticks=5*, *min_color='0000ff'*, *mid_color='ffffff'*, *max_color='ff0000'*, *labelattr=None*, *log_display=True*, ***kwargs*)

Create a key including color gradient and labels indicating associated values, returning a `pyx` canvas.

Parameters

- **min_score** (*float*) – The minimum value of the key scale.
- **max_score** (*float*) – The maximum value of the key scale.
- **height** (*float*) – The height of the gradient bar in whatever units `pyx` is using.
- **width** (*float*) – The width of the gradient bar in whatever units `pyx` is using.
- **labelformat** (*str.*) – A string denoting the format for displaying number labels using the string formatting style from Python <= 2.6.
- **orientation** – Indicates where labels are placed relative to gradient bar. This parameter will accept 'left', 'right', 'top', and 'bottom'.
- **num_ticks** (*int.*) – Indicates how many evenly-spaced tick marks and associated labels to insert. This can be zero for no labels or greater than one. Labels are inserted at the minimum and maximum values first with remaining ticks occuring evenly distributed between the extremes.

- **min_color** (*str.*) – This is a hex color code ("rrggbb" where each pair ranges from 00-ff) specifying the color associated with the minimum plot value. This variable is used to create a color gradient for plotting along with max_color and mid_color.
- **mid_color** (*str.*) – This is a hex color code ("rrggbb" where each pair ranges from 00-ff) specifying the color associated with the minimum plot value. This can be set to None to create a gradient ranging from min_color to max_color or to a hex color to create a divergent gradient.
- **max_color** (*str.*) – This is a hex color code ("rrggbb" where each pair ranges from 00-ff) specifying the color associated with the maximum plot value. This variable is used to create a color gradient for plotting along with min_color and mid_color.
- **labelattr** (*str.*) – A list of pyx attributes to be passed to the text function.
- **log_display** (*bool.*) – If True, min_score and max_score are taken to be logged values and so labels are evenly spaced in log space but converted to normal space for display.

Returns `Pxy` canvas object.

`hifive.plotting.`**`plot_upper_array`**(*data*, *maxscore=None*, *minscore=None*, *symmetricscaling=True*, *logged=True*, *min_color='0000ff'*, *mid_color='ffffff'*, *max_color='ff0000'*, *returnscale=False*, ***kwargs*)

Fill in and rescale bitmap from a 5C or HiC upper array.

Parameters

- **data** (*numpy array*) – A two-dimensional compact array of interaction data.
- **maxscore** (*float*) – A ceiling value to cutoff scores at for plot color.
- **minscore** (*float*) – A floor value to cutoff scores at for plot color.
- **symmetricscaling** (*bool.*) – Indicates whether to recenter data for scaling or maintain scores about zero.
- **logged** (*bool.*) – Indicates whether to use log2 values of scores for color values.
- **min_color** (*str.*) – This is a hex color code ("rrggbb" where each pair ranges from 00-ff) specifying the color associated with the minimum plot value. This variable is used to create a color gradient for plotting along with max_color and mid_color.
- **mid_color** (*str.*) – This is a hex color code ("rrggbb" where each pair ranges from 00-ff) specifying the color associated with the minimum plot value. This can be set to None to create a gradient ranging from min_color to max_color or to a hex color to create a divergent gradient.
- **max_color** (*str.*) – This is a hex color code ("rrggbb" where each pair ranges from 00-ff) specifying the color associated with the maximum plot value. This variable is used to create a color gradient for plotting along with min_color and mid_color.
- **returnscale** (*bool.*) – Indicates whether to return a list containing the bitmap, minimum score, and maximum score, or just the bitmap.

Returns `PIL` bitmap object and if requested, minimum and maximum scores.

CHAPTER 14

Release History

14.1 1.1 (2015-08-26)

- Added multi-resolution heatmaps (MRH).
- Added stand-alone script for plotting MRH data.
- Fixed requirements resolution for pip loading without requirements already installed.
- Re-implemented Poisson model for HiC probability algorithm.
- Lowered memory requirement for HiC data loading.
- Added dockerfile for building docker image.

14.2 1.0.3 (2015-04-23)

- Expanded functionality of Express to include support to Knight-Ruiz algorithm to both 5C and HiC algorthims.
- Added binary counts option to Express in HiC.
- Added non-logged counts functionality to Express in 5C.
- Added pseudo-count option to Binning in HiC.
- Dramatically sped up heatmap creation for ‘fend’-corrected heatmaps using probability or express corrections.
- Added binary distance-dependence calculation alongside standard.

14.3 1.0.2 (2015-04-16)

- Fixed Express algorithm stopping criteria.
- Updated Probability algorithm and documentation.
- Fixed bug introduced into HiC binning algorithm.
- Added binning documentation.
- Added Travis automated testing.

CHAPTER 15

Indices and tables

- genindex
- modindex
- search

h

hifive.fend, 58
hifive.fivec, 66
hifive.fivec_binning, 87
hifive.fivec_data, 60
hifive.fragment, 57
hifive.hic, 75
hifive.hic_binning, 93
hifive.hic_data, 62
hifive.plotting, 100

B

bin_cis_array() (in module hifive.fivec_binning), 87
bin_cis_array() (in module hifive.hic_binning), 93
bin_trans_array() (in module hifive.hic_binning), 94

C

cis_heatmap() (hifive.fivec.FiveC method), 66
cis_heatmap() (hifive.hic.HiC method), 76

D

dynamically_bin_cis_array() (in module hifive.fivec_binning), 88
dynamically_bin_cis_array() (in module hifive.hic_binning), 95
dynamically_bin_trans_array() (in module hifive.fivec_binning), 89
dynamically_bin_trans_array() (in module hifive.hic_binning), 96

E

export_to_mat() (hifive.hic_data.HiCData method), 63

F

Fend (class in hifive.fend), 58
filter_fends() (hifive.hic.HiC method), 78
filter_fragments() (hifive.fivec.FiveC method), 68
find_binning_fend_corrections() (hifive.hic.HiC method), 78
find_binning_fragment_corrections() (hifive.fivec.FiveC method), 68
find_cis_signal() (in module hifive.fivec_binning), 89
find_cis_signal() (in module hifive.hic_binning), 96
find_distance_parameters() (hifive.fivec.FiveC method), 69
find_distance_parameters() (hifive.hic.HiC method), 79
find_express_fend_corrections() (hifive.hic.HiC method), 80
find_express_fragment_corrections() (hifive.fivec.FiveC method), 69
find_multiresolution_heatmap() (in module hifive.hic_binning), 98
find_probability_fend_corrections() (hifive.hic.HiC method), 81
find_probability_fragment_corrections() (hifive.fivec.FiveC method), 70
find_trans_mean() (hifive.fivec.FiveC method), 71
find_trans_means() (hifive.hic.HiC method), 81
find_trans_signal() (in module hifive.fivec_binning), 90
find_trans_signal() (in module hifive.hic_binning), 98
FiveC (class in hifive.fivec), 66
FiveCData (class in hifive.fivec_data), 60
Fragment (class in hifive.fragment), 57

H

HiC (class in hifive.hic), 75
HiCData (class in hifive.hic_data), 62
hifive.fend (module), 58
hifive.fivec (module), 66
hifive.fivec_binning (module), 87
hifive.fivec_data (module), 60
hifive.fragment (module), 57
hifive.hic (module), 75
hifive.hic_binning (module), 93
hifive.hic_data (module), 62
hifive.plotting (module), 100

L

learn_fend_3D_model() (hifive.hic.HiC method), 82
load() (hifive.fend.Fend method), 59
load() (hifive.fivec.FiveC method), 71
load() (hifive.fivec_data.FiveCData method), 60
load() (hifive.fragment.Fragment method), 57
load() (hifive.hic.HiC method), 82
load() (hifive.hic_data.HiCData method), 63
load_data() (hifive.fivec.FiveC method), 71
load_data() (hifive.hic.HiC method), 82
load_data_from_bam() (hifive.fivec_data.FiveCData method), 60
load_data_from_bam() (hifive.hic_data.HiCData method), 63

load_data_from_counts() (hifive.fivec_data.FiveCData method), 61
load_data_from_mat() (hifive.hic_data.HiCData method), 64
load_data_from_raw() (hifive.hic_data.HiCData method), 65
load_fends() (hifive.fend.Fend method), 59
load_fragments() (hifive.fragment.Fragment method), 57

P

plot_compact_array() (in module hifive.plotting), 101
plot_diagonal_from_compact_array() (in module hifive.plotting), 101
plot_diagonal_from_upper_array() (in module hifive.plotting), 102
plot_fivec_compact_heatmap_dict() (in module hifive.plotting), 103
plot_fivec_heatmap() (in module hifive.plotting), 103
plot_full_array() (in module hifive.plotting), 104
plot_hic_heatmap() (in module hifive.plotting), 104
plot_key() (in module hifive.plotting), 105
plot_upper_array() (in module hifive.plotting), 106

R

reset_filter() (hifive.hic.HiC method), 82

S

save() (hifive.fend.Fend method), 60
save() (hifive.fivec.FiveC method), 71
save() (hifive.fivec_data.FiveCData method), 62
save() (hifive.fragment.Fragment method), 58
save() (hifive.hic.HiC method), 83
save() (hifive.hic_data.HiCData method), 66

T

trans_heatmap() (hifive.fivec.FiveC method), 72
trans_heatmap() (hifive.hic.HiC method), 83

W

write_heatmap() (hifive.fivec.FiveC method), 74
write_heatmap() (hifive.hic.HiC method), 84
write_heatmap_dict() (in module hifive.fivec_binning), 92
write_heatmap_dict() (in module hifive.hic_binning), 100
write_multiresolution_heatmap() (hifive.hic.HiC method), 86
