# Supplementary material for: HiFive: a tool suite for easy and efficient HiC and 5C data analysis
Source: Genome Biol. 2015 Oct 24;16:237. doi: 10.1186/s13059-015-0806-y (PMC5410870; doi:10.1186/s13059-015-0806-y)
Supplement: Supplementary file 3 — A tar archive containing all scripts using to generate the data, analyses, and figures presented in this paper. (BZ2 20009 kb) [file 13059_2015_806_MOESM3_ESM.bz2 › HiFive_analysis_package/Figures/figure1.pdf]

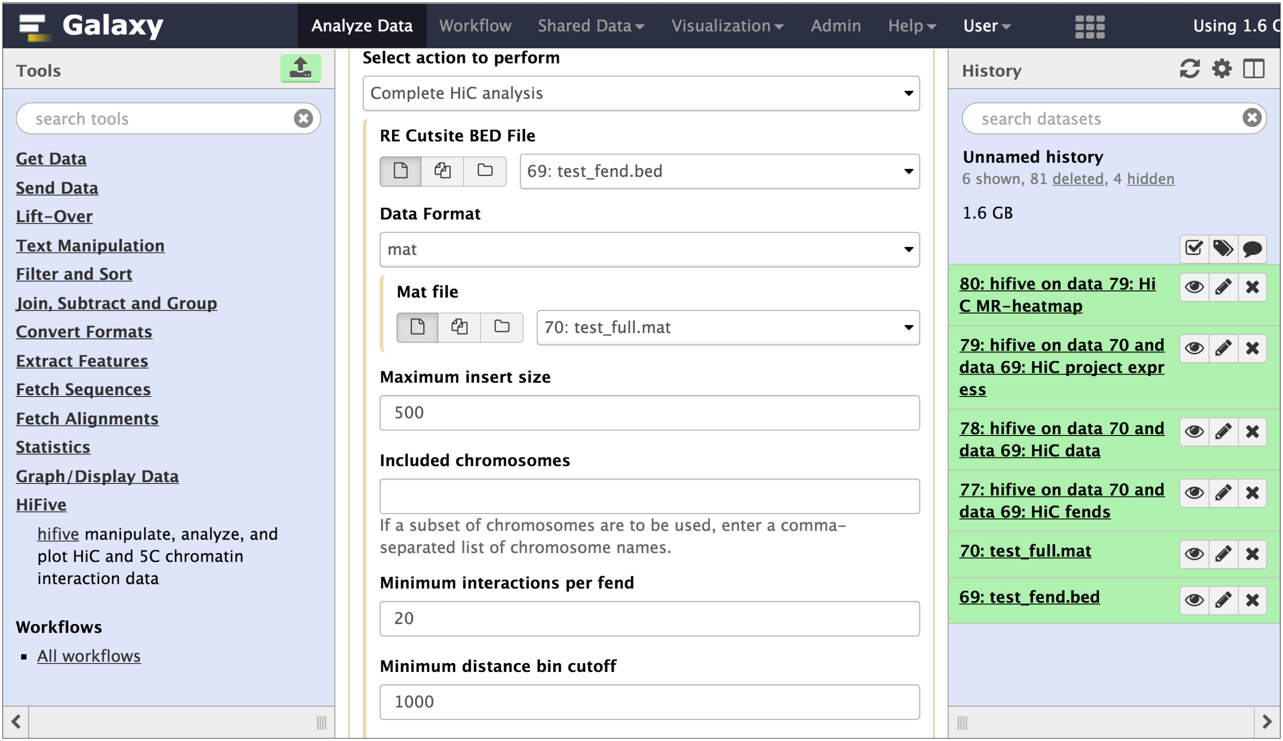
Galaxy
Analyze Data
Workflow
Shared Data
Visualization
Admin
Help
User
Using 1.6 G
Tools
search tools
Get Data
Send Data
Lift-Over
Text Manipulation
Filter and Sort
Join, Subtract and Group
Convert Formats
Extract Features
Fetch Sequences
Fetch Alignments
Statistics
Graph/Display Data
HiFive
hifive manipulate, analyze, and plot HiC and 5C chromatin interaction data
Workflows
All workflows
Select action to perform
Complete HiC analysis
RE Cutsite BED File
69: test_fend.bed
Data Format
mat
Mat file
70: test_full.mat
Maximum insert size
500
Included chromosomes
If a subset of chromosomes are to be used, enter a comma-separated list of chromosome names.
Minimum interactions per fend
20
Minimum distance bin cutoff
1000
History
search datasets
Unnamed history
6 shown, 81 deleted, 4 hidden
1.6 GB
80: hifive on data 79: HiC MR-heatmap
79: hifive on data 70 and data 69: HiC project express
78: hifive on data 70 and data 69: HiC data
77: hifive on data 70 and data 69: HiC fends
70: test_full.mat
69: test_fend.bed
